# Fluorocyclisation via I(I)/I(III) catalysis: a concise route to fluorinated oxazolines

**DOI:** 10.3762/bjoc.14.88

**Published:** 2018-05-09

**Authors:** Felix Scheidt, Christian Thiehoff, Gülay Yilmaz, Stephanie Meyer, Constantin G Daniliuc, Gerald Kehr, Ryan Gilmour

**Affiliations:** 1 Organisch-Chemisches Institut, Westfälische Wilhelms-Universität Münster, Corrensstraße 40, 48149 Münster, Germany

**Keywords:** catalysis, cyclisation, fluorination, *gauche* effect, hypervalent iodine, oxazolines

## Abstract

Herein, we describe a catalytic fluorooxygenation of readily accessible *N*-allylcarboxamides via an I(I)/I(III) manifold to generate 2-oxazolines containing a fluoromethyl group. Catalysis is conditional on the oxidation competence of Selectfluor^®^, whilst HF serves as both a fluoride source and Brønsted acid activator. The C(sp^3^)–F bond of the mono-fluoromethyl unit and the C(sp^3^)–O bond of the ring are aligned in a *synclinal* relationship thereby engaging in stabilising hyperconjugative interactions with *vicinal*, electron-rich σ-bonds (σ_C–C_→σ*_C–F_ and σ_C–H_→σ*_C–O_). This manifestation of the stereoelectronic *gauche* effect was established by X-ray crystallographic analysis of a representative example. Given the importance of fluorine in drug discovery, its ability to modulate conformation, and the prevalence of the 2-oxazoline scaffold in Nature, this strategy provides a rapid entry into an important bioisostere class.

## Introduction

**Figure 1:**
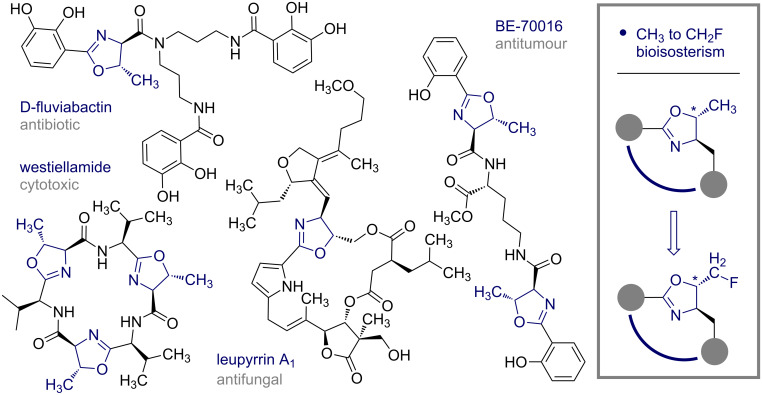
Selected examples of bioactive compounds containing the 2-oxazoline motif.

Marine and terrestrial natural product bioprospecting has established a broad spectrum of structurally complex, bioactive metabolites containing the venerable 2-oxazoline unit [1–2]. This diversity is exemplified by the siderophore antibiotic D-fluviabactin, the cytotoxic agent westiellamide, the antifungal macrodiolide leupyrrin A_1_ and the antitumour compound BE-70016 (Figure 1). In addition, synthetic polymers based on the 2-oxazoline building block constitute versatile platforms for a range of biomedical applications ranging from drug delivery through to tissue engineering [3–4]. Collectively, the importance of the 2-oxazoline scaffold for translational research, together with its strategic value in the design of chiral ligands and auxiliaries [5–7], has culminated in a rich and innovative arsenal of synthetic methods.

To contribute to the current catalysis ordnance for the preparation of 2-oxazolines, and provide a direct route to 5-fluoromethyl derivatives from simple unactivated alkenes, the fluorocyclisation of *N*-allylcarboxamides facilitated by the in situ generation of *p*-TolIF_2_ was envisaged [8–11]. Since hydrogen and hydroxy groups are often substituted by fluorine in molecular editing processes [12], this transformation would provide facile access to a bioisostere of the parent scaffold (Figure 1, right).

**Figure 2:**
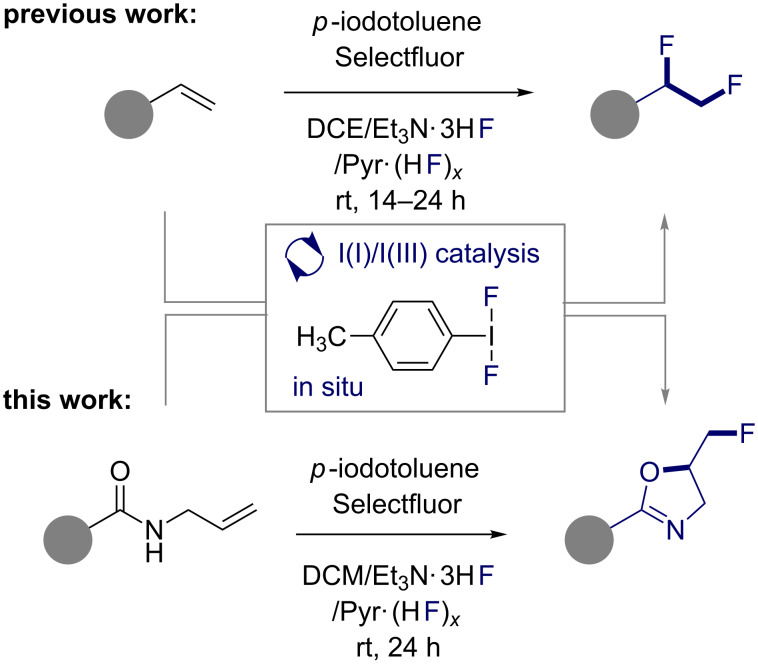
The catalytic difluorination of alkenes (top) and the proposed fluorocyclisation via the same I(I)/I(III) manifold (bottom).

In recent years, I(I)/I(III) catalysis has emerged as a powerful and expansive platform for the generation of structural complexity [13–24]. Motivated by the noticeable absence of mild, catalysis-based strategies to generate the *vicinal* difluoroethylene motif directly from simple alkenes [25–27], we recently exploited I(I)/I(III) catalysis to enable this transformation [28–29]. Employing Selectfluor^®^ as the terminal oxidant, it was possible to generate *p*-TolIF_2_ in situ from *p*-iodotoluene and an inexpensive HF source [30–35]. This strategy proved to be mild and general, smoothly converting terminal olefins to the corresponding 1,2-difluoroethylene unit; a substructure that may be considered a chiral, hybrid bioisostere of the Et and CF_3_ groups (Figure 2, top) [36].

Since the success of this process is contingent on the efficient generation of *p*-TolIF_2_ in situ, the platform lends itself to related oxidative transformations. To that end, it was envisaged that the protocol could be effectively translated to the fluorocyclisation of readily accessible *N*-allylcarboxamides (Figure 2, bottom). Whilst the initial phase of catalysis would resemble that of the catalytic difluorination, the presence of the amide would allow the original reaction path to be intercepted to generate a 2-oxazoline with an exocyclic fluoromethyl unit.

## Results and Discussion

**Optimisation:** As a starting point, the conversion of *N*-allylbenzamide (**1**) to the corresponding 2-phenyloxazoline **2** was investigated (Table 1). Reactions were performed in DCE (0.2 mol·L^−1^) with 20 mol % catalyst loading, and using Selectfluor^®^ as the oxidant. An initial reaction screen, based on the conditions reported for our *vicinal* difluorination study [9], began with an exploration of the effect of amine/HF ratio. This was deemed prudent due to the perceived likelihood that HF also functions as a Brønsted acid activator in catalysis. Employing an amine/HF ratio of 1:4.5, product formation was observed (Table 1, entry 1, 46%). Reducing this ratio to 1:3 had a detrimental effect on catalysis efficiency, generating the product in <5% yield (Table 1, entry 2). Increasing the ratio to 1:7.5 and 1:9.23 (Olah’s reagent) restored catalysis efficiency but did not surpass previous observations (44 and 46% yields, Table 1, entries 3 and 4, respectively). For comparison, the reaction was attempted using Pyr·HF (6 equiv) but this alteration had an adverse effect on yield (27%, Table 1, entry 5). Based on these findings, the remainder of the study was performed with an amine/HF ratio of 1:4.5. Reducing the concentration from 0.2 mol·L^−1^ to 0.1 mol·L^−1^ led to a large increase in yield (81%, Table 1, entry 6).

**Table 1:** Optimisation of reaction conditions for a benchmark transformation (**1**→**2**).^a^

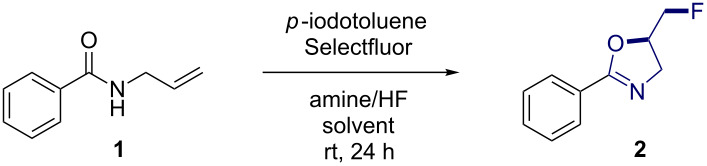						

Entry	Concentration [mol·L^−1^]	Solvent	Catalyst loading [mol %]	Amine/HF ratio	Conversion^b^ [%]	Yield [%]^c^

1	0.2	DCE	20	1:4.5	>95	46
2	0.2	DCE	20	1:3	50	<5
3	0.2	DCE	20	1:7.5	>95	44
4	0.2	DCE	20	1:9.23	>95	46
5	0.2	DCE	20	Pyr·HF (6 equiv)^d^	>95	27
6	0.1	DCE	20	1:4.5	>95	81
7	0.1	toluene	20	1:4.5	>95	72
8	0.1	MeCN	20	1:4.5	78	47
9	0.1	THF	20	1:4.5	34	<5
10	0.1	DCM	20	1:4.5	>95	>95
**11**	**0.1**	**DCM**	**10**	**1:4.5**	**>95**	**>95 (67)^e^**
12	0.1	DCM	2.5	1:4.5	40	30
13	0.1	DCM	0	1:4.5	<5	<5

^a^Standard reaction conditions: *N*-allylbenzamide (200 µmol), catalyst *p*-iodotoluene, solvent, amine/HF source 1:1 (v/v), Selectfluor^®^ (1.5 equiv), ambient temperature, 24 h; ^b^Determined from the ^1^H NMR spectrum using ethyl fluoroacetate (1.0 equiv) as internal standard; ^c^Determined from the ^19^F NMR spectrum using ethyl fluoroacetate (1.0 equiv) as an internal standard; ^d^Reaction conducted with 1 mL of solvent; ^e^Yield after column chromatography on silica gel. Reduction in yield is due to hydrolysis. DCE: 1,2-dichloroethane.

Whilst solvents such as toluene, acetonitrile and THF were less effective than DCE (Table 1, entries 7–9), switching to DCM led to full consumption of the starting material and a quantitative NMR yield (Table 1, entry 10). In a final optimisation round, the catalyst loading was reduced to 10 mol % with no discernable effect on performance (Table 1, entry 11). However, further decreasing the loading to 2.5 mol % demonstrates the limits of the system (30% yield, Table 1, entry 12). Finally, for completeness, the control experiment in the absence of *p-*TolI was performed and confirms the role of this species in catalysis.

**Figure 3:**
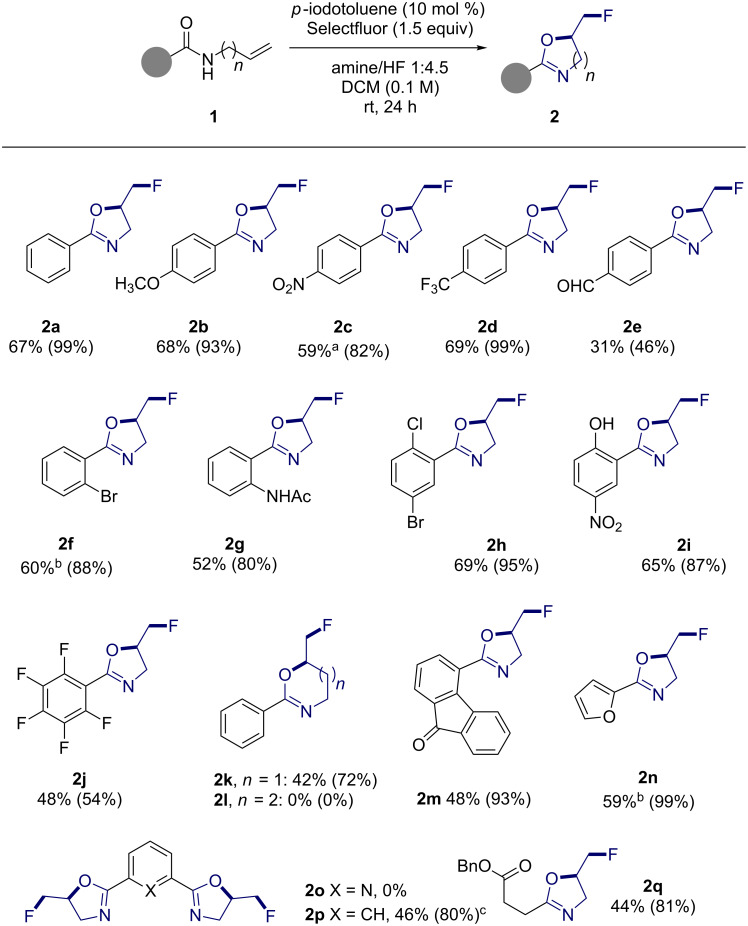
Substrate scope. ^a^Reaction conducted on 1 mmol scale. ^b^Reaction time increased to 40 hours. ^c^Reaction time increased to 32 hours. Yields refer to isolated values whilst NMR yields are given in parentheses (^19^F NMR using ethyl fluoroacetate as an internal standard).

**Establishing scope:** With a general procedure having been developed, attention was then focused on establishing the scope of the transformation (Figure 3). To explore the effect of changes to the aryl ring, compared to the parent scaffold **2a**, representative *N*-allylcarboxamides containing the *p*-OCH_3_, *p*-NO_2_ and *p*-CF_3_ substituents were exposed to the general conditions (to generate **2b**, **2c** and **2d**, respectively). These transformations proceeded smoothly to deliver the target 2-oxazolines in good yields (up to 69%) and in the case of compound **2c**, the fluorocyclisation was performed on a 1 mmol scale with no impact on the yield. However, the aldehyde derivative **2e** proved to be more challenging and was isolated in a modest 31% yield. Systems containing *ortho*-substituents (**2f** and **2g**) were also well tolerated but in the case of **2f** it was necessary to extend the reaction time to 40 h. Disubstitution patterns such as in **2h** and **2i**, the latter of which contains a free phenol moiety, were also tolerated (69% and 65% yield, respectively), as was the highly deactivated pentafluorophenyl analogue **2j** (48%). To briefly explore the effect of chain length on efficiency, the cyclisation of *N*-(but-3-en-1-yl)benzamide and *N*-(pent-4-en-1-yl)benzamide was explored (to generate **2k** and **2l**, respectively). Unsurprisingly, whilst the 6-membered ring formed in 42% yield, cyclisation to form the analogous 7-membered ring failed. It was, however, possible to generate heterocyclic species such as the 9-fluorenonyl-substituted oxazoline **2m** (48%) and the furan **2n** (59%). Whilst it was not possible to generate the bisoxazoline **2o** (X = N), the analogous carbogenic scaffold **2p** (X = CH) formed in 46% yield. Finally, although more challenging, it was also possible to generate an aliphatic 2-oxazoline (**2q**) in a modest 44% yield.

**Scheme 1:**
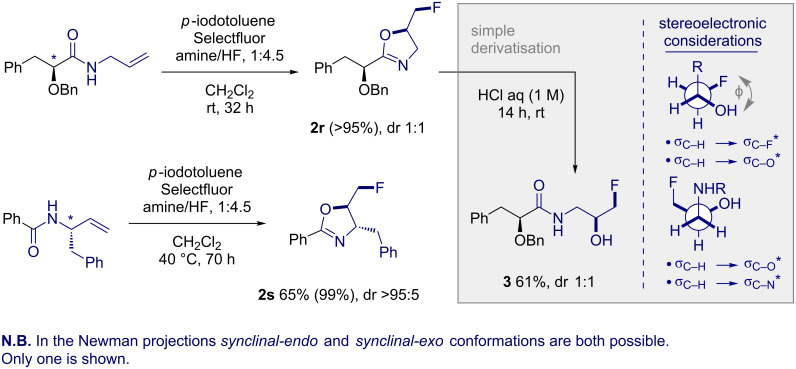
Exploring diastereocontrol and the synthesis of the fluorohydrin **3**. Yields in parentheses were determined by ^19^F NMR using ethyl fluoroacetate as an internal standard. Unless otherwise stated, yields refer to isolated values.

Finally, to explore possible diastereocontrol in the cyclisation event, oxazoline **2r** was generated from the corresponding α-chiral amide under standard conditions. Analysis of the crude reaction mixture by ^19^F NMR allowed a yield of >95% to be determined and a 1:1 dr. This is to be expected given the remote nature of the stereocentre. It is important to note that attempts to separate this compound by column chromatography resulted in significant hydrolysis. Consequently, the oxazoline was exposed to acidic media and quantitatively hydrolysed to the fluorohydrin **3** in 61% yield (Scheme 1). In contrast, the cyclisation to form **2s** was highly diastereoselective on account of the proximal nature of the stereocentre (65%, dr >95:5).

**Figure 4:**
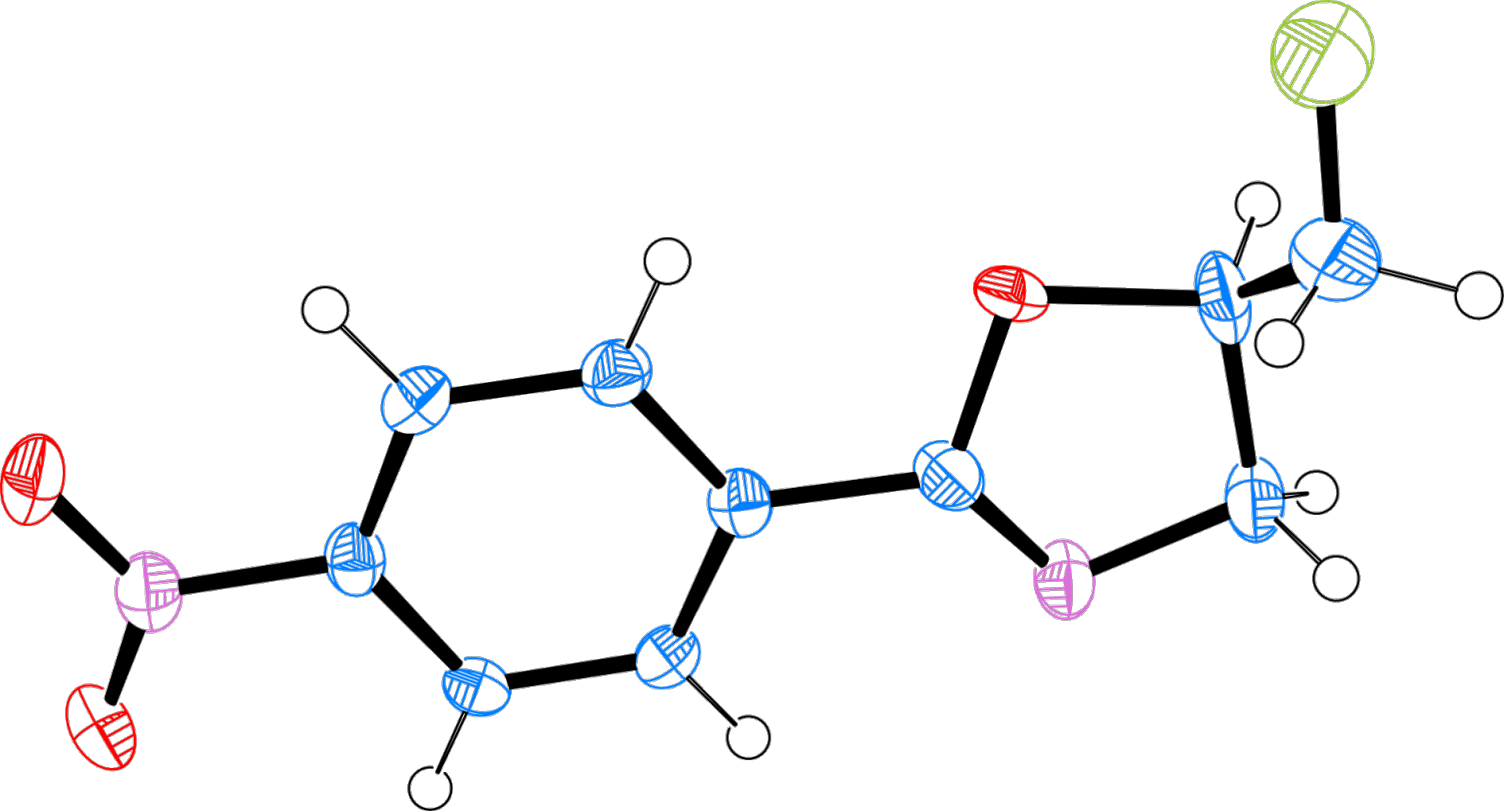
X-ray molecular structure of compound **2c**. Thermal ellipsoids shown at the 50% propability level. Torsion angle (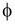
_F1–C10–C9–O1_ −73.4°) consistent with the fluorine *gauche* effect. CCDC number 1815371.

Compound **3** is noteworthy on account of the β-amino alcohol and β-fluoro alcohol motifs that collectively preorganised the propyl chain. Stabilising hyperconjugative interactions manifest themselves in the characteristic *gauche* conformations around the two respective torsion angles [37–38]. In this case, it is also highly probable that hydrogen bonding will reinforce these conformational preferences. Whilst it was not possible to isolate crystals of **3** that were suitable for X-ray analysis, it was possible to unambiguously establish the structure of oxazoline **2c** bearing a *p*-NO_2_ group (Figure 4 and Table 2) [39]. The molecular structure reveals the expected *gauche* arrangement with a dihedral angle 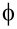
_FCCO_ ≈ −73.4° due to σ_C–C_→σ_C–F_* and σ_C–H_→σ_C–O_* interactions. This observation is in agreement with the fluorine *gauche* effect.

**Table 2:** Crystallographic data for compound **2c**.

Entry	Data

formula	C_10_H_9_FN_2_O_3_
*M*_r_	224.19
crystal size, mm^3^	0.032 × 0.162 × 0.247
crystal system	orthorhombic
space group	*P*na2_1_
cell constants	
*a*, Å	10.0315(3)
*b*, Å	15.4164(5)
*c*, Å	6.5161(2)
*V*, Å^3^	1007.71(5)
*Z*	4
*D*_x_, Mg m^−3^	1.48
μ, mm^−1^	1.06
*F*(000), e	464
*T*, K	100(2)
λ, Å	1.54178
2θ_max_, deg	137
transmissions	0.78–0.97
refl. meas./indep./*R*_int_	10003/1813/0.034
ref. parameters	182
restraints	118
*R* [*F* ≥ 4 σ(*F*)]	0.032
*w*R (*F*^2^, all refl.)	0.086
*S*	1.05
Δρ_max_, e Å^−3^	0.145/−0.194

## Conclusion

An operationally simple route to 5-fluoromethyl-2-oxazolines from readily accessible *N*-allylcarboxamides is disclosed based on an I(I)/I(III) catalysis manifold. This metal-free fluorocyclisation employs *p*-iodotoluene (10 mol %) as an inexpensive organocatalyst and Selectfluor^®^ as oxidant. The optimal amine/HF ratio (1:4.5) is easily obtained by combining commercially available triethylamine tris(hydrogenfluoride) (Et_3_N·3HF) and Olah’s reagent (Pyr·HF). Broad functional group tolerance is observed in the products, the structures of which display the stereoelectronic fluorine *gauche* effect.

## Supporting Information

File 1 Experimental part.
