# Supplementary material for: Fluorocyclisation via I(I)/I(III) catalysis: a concise route to fluorinated oxazolines
Source: Beilstein J Org Chem. 2018 May 9;14:1021–7. doi: 10.3762/bjoc.14.88 (PMC6009682; doi:10.3762/bjoc.14.88)
Supplement: File 1 — Experimental part. [file Beilstein_J_Org_Chem-14-1021-s001.pdf]

Supporting information

for

Fluorocyclisation via I(I)/I(III) catalysis: a concise route to fluorinated oxazolines

Felix Scheidt[‡], Christian Thiehoff[‡], Gülay Yilmaz, Stephanie Meyer, Constantin G. Daniliuc[§], Gerald Kehr and Ryan Gilmour*[¶]

Address: Organisch-Chemisches Institut, Westfälische Wilhelms-Universität Münster

Correnssstraße 40, 48149 Münster, Germany

Email: Ryan Gilmour - ryan.gilmour@uni-muenster.de

[‡]These authors contributed equally

*Corresponding author

[§]X-ray crystallographer

[¶]Homepage: http://www.uni-muenster.de/Chemie.oc/gilmour

Experimental part

Table of Contents

General information S3

X-ray crystal structure analysis of compound **2c** S4

Synthesis of substrates S7

Fluorocyclisations of (homo-)allyl amides S28

Selected NMR spectra S46

References S91

General information

All commercially available reagents were purchased as reagent grade from *Sigma Aldrich, Merck*, *Alfa Aesar*, or *abcr* and were used without further purification. Where indicated tetrahydrofuran and dichloromethane were dried over activated alumina prior to use. Solvents for extractions or chromatographic purifications were bought as technical grade and distilled on a rotary evaporator prior to use. For analytical thin-layer chromatography, glass plates coated with SiO_2-60 F_{254} were used from *Merck*. They were visualized with UV-light or with $KMnO_4$ or CAM solution. Flash column chromatography was performed on SiO_2-60 (230–400 mesh ASTM, *Fluka*). The NMR measurements were performed at the given temperature on a *Bruker AV300, AV400, Agilent DD2 500* or an *Agilent DD2 600* by the NMR department of the Organisch-Chemisches Institut, Westfälische Wilhelms-Universität Münster. The chemical shifts are referenced to the residual solvent peak as the internal standard. The multiplicity is abbreviated as follows: s (singlet), d (doublet), t (triplet), m (multiplet) and br (broad). The coupling constant *J* is given in Hz (Hertz). The given assignments are supported by additional 1D and 2D NMR experiments. The melting points were determined on a *Büchi* B-545 melting point apparatus with open glass capillaries. The IR measurements were performed on a *Perkin-Elmer 100 FT-IR* spectrometer and the intensities of the bands are assigned as follows: w (weak), m (medium), s (strong). The optical rotations were measured on a *JASCO P2000* polarimeter. High-resolution mass spectrometry was performed by the MS service of the Organisch-Chemisches Institut, Westfälische Wilhelms-Universität Münster.

X-ray diffraction: Data sets for compound **2c** were collected with a D8 Venture Dual Source 100 CMOS diffractometer. Programs used: data collection: APEX3 V2016.1-0[1]; cell refinement: SAINT V8.37A[1]; data reduction: SAINT V8.37A[1]; absorption correction, SADABS V2014/7[1]; structure solution SHELXT-2015[2]; structure refinement SHELXL-2015[2]. *R*-values are given for observed reflections, and wR^2 values are given for all reflections.

Exceptions and special features: For compound **2c** the fluoromethyl oxazoline unit was found disordered over two positions. Several restraints (SADI, ISOR, EADP, EXYZ and SIMU) were used in order to improve refinement stability.

X-ray crystal structure analysis of compound 2c

X-ray crystal structure analysis of **2c** (**CCDC: 1815371**): A colorless plate-like specimen of $C_{10}H_9FN_2O_3$, approximate dimensions 0.032 mm × 0.162 mm × 0.247 mm, was used for the X-ray crystallographic analysis. The X-ray intensity data were measured. A total of 1356 frames were collected. The total exposure time was 22.60 hours. The frames were integrated with the Bruker SAINT software package using a wide-frame algorithm. The integration of the data using an orthorhombic unit cell yielded a total of 10003 reflections to a maximum θ angle of 68.28° (0.83 Å resolution), of which 1813 were independent (average redundancy 5.517, completeness = 98.6%, R_{int} = 3.42%, R_{sig} = 2.81%) and 1671 (92.17%) were greater than $2\sigma(F^2)$. The final cell constants of a = 10.0315(3) Å, b = 15.4164(5) Å, c = 6.5161(2) Å, volume = 1007.71(5) $Å^3$, are based upon the refinement of the XYZ-centroids of 7404 reflections above 20 σ(I) with 14.75° < 2θ < 136.5°. Data were corrected for absorption effects using the multi-scan method (SADABS). The ratio of minimum to maximum apparent transmission was 0.836. The calculated minimum and maximum transmission coefficients (based on crystal size) are 0.7800 and 0.9670. The structure was solved and refined using the Bruker SHELXTL Software Package, using the space group $Pna2_1$, with Z = 4 for the formula unit, $C_{10}H_9FN_2O_3$. The final anisotropic full-matrix least-squares refinement on F^2 with 182 variables converged at R1 = 3.17%, for the observed data and wR2 = 8.63% for all data. The goodness-of-fit was 1.049. The largest peak in the final difference electron density synthesis was 0.145 $e^-/Å^3$ and the largest hole was −0.194 $e^-/Å^3$ with an RMS deviation of 0.037 $e^-/Å^3$. On the basis of the final model, the calculated density was 1.478 g/cm^3 and F(000), 464 e^-.

Table S1: Crystallographic data for compound **2c**.

Compound	**2c**
Formula	$C_{10}H_9FN_2O_3$
M_r	224.19
Crystal size, mm^3	0.032 x 0.162 x 0.247
Crystal system	orthorhombic
Space group	$Pna2_1$
Cell constants	
a, Å	10.0315(3)
b, Å	15.4164(5)
c, Å	6.5161(2)
V, $Å^3$	1007.71(5)
Z	4
D_x, Mg m^{-3}	1.48
μ, mm^{-1}	1.06
F(000), e	464
T, K	100(2)
λ, Å	1.54178
$2\theta_{max}$, deg	137
Transmissions	0.78 – 0.97
Refl. meas. / indep. / R_{int}	10003 / 1813 / 0.034
Ref. parameters	182
Restraints	118
R [$F \geq 4\ \sigma(F)$]	0.032
wR (F^2, all refl.)	0.086
S	1.05
$\Delta\rho_{max}$, e $Å^{-3}$	0.145 / -0.194

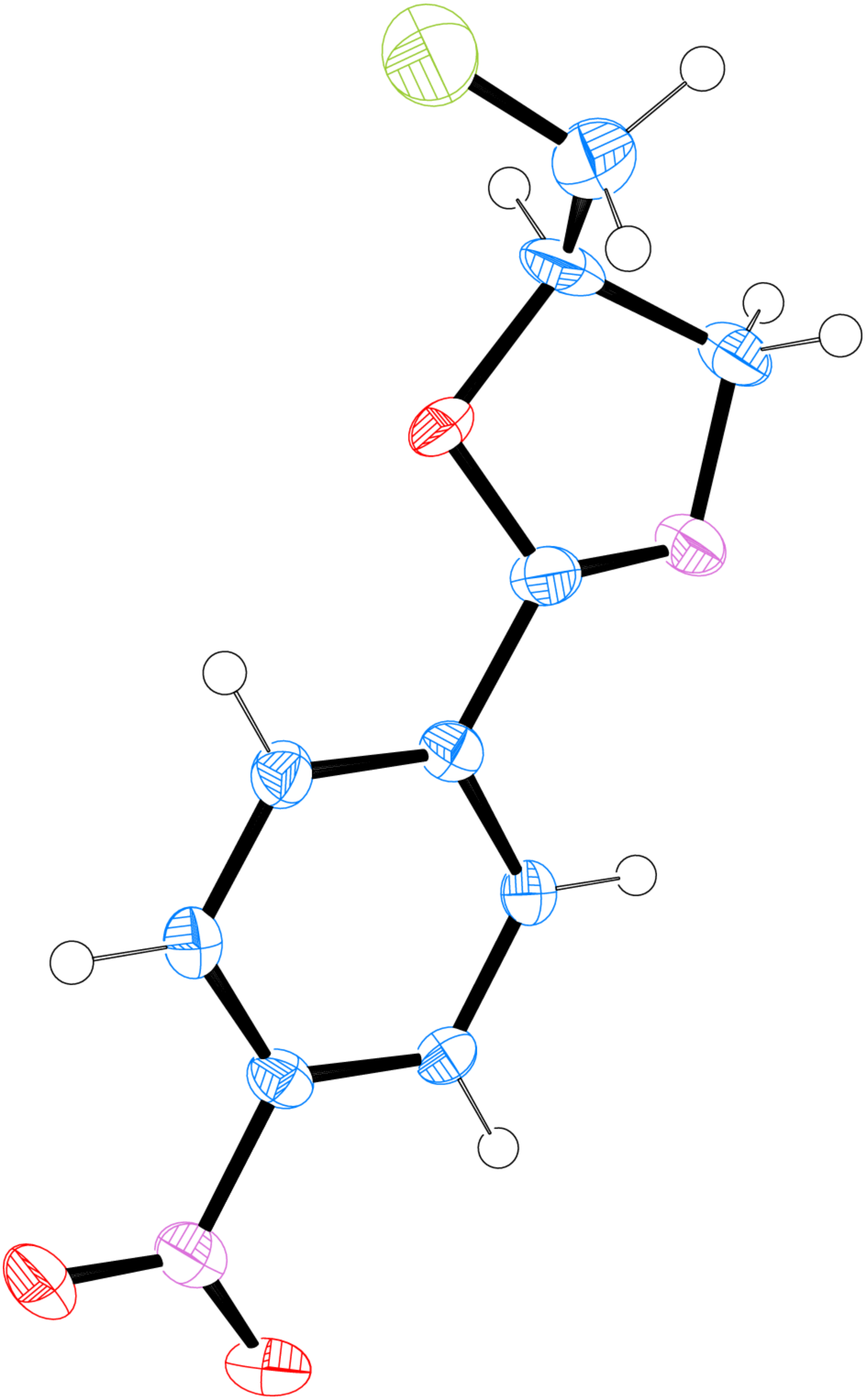

Figure S1: X-ray molecular structure of compound **2c**. Thermal ellipsoids shown at the 50% probability level.

Synthesis of substrates

N-Allylbenzamide (1a)[3]

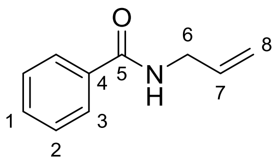

This compound was prepared according to a modified procedure by Prediger et al.[3]: To a solution of triethylamine (1.66 mL, 12.0 mmol, 1.2 equiv) and allylamine (0.83 mL, 11.0 mmol, 1.2 equiv) in dichloromethane (25 mL) was slowly added benzoyl chloride (1.16 mL, 10 mmol, 1.0 equiv). After stirring the mixture for 16 h at ambient temperature, dichloromethane (30 mL) and aqueous HCl (40 mL, 1 M) were added. The organic phase was separated, dried over $MgSO_4$ and all volatiles were removed in vacuo. The remaining oily residue was purified by column chromatography (cyclohexane : ethyl acetate, 2:1) to give the product (1.49 g, 9.3 mmol, 93%) as a pale yellow oil.

R*f*: 0.50 (cyclohexane : ethyl acetate, 1:1).

^{1}H NMR (400 MHz, $CDCl_3$, 298 K): δ [ppm] = 7.79 (m, 2H, H-C3), 7.49 (m, 1H, H-C1), 7.42 (m, 2H, H-C2), 6.32 (br-s, 1H, NH), 5.93 (ddt, $^3J_{HH}$ = 17.1 Hz, 10.2 Hz, 5.7 Hz, 1H, H-C7), 5.26 (dq, $^3J_{HH}$ = 17.1 Hz, $^2J_{HH}$ = $^4J_{HH}$ = 1.5 Hz, 1H, H^Z-C8), 5.18 (dq, $^3J_{HH}$ = 10.2 Hz, $^2J_{HH}$ = $^4J_{HH}$ = 1.5 Hz, 1H, H^E-C8), 4.08 (tt, $^3J_{HH}$ = 5.7 Hz, $^4J_{HH}$ = 1.5 Hz, 1H, H-C6).

The analytical data are in agreement with literature values[3].

N-Allyl-4-methoxybenzamide (1b)[4]

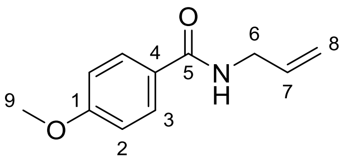

The compound was prepared according to the procedure for **1c**[5]. To a stirred solution of 1-(3-dimethylaminopropyl)-3-ethylcarbodiimid hydrochloride (1.12 g, 5.8 mmol, 1.2 equiv) and 4-(dimethylamino)-pyridine (855 mg, 7.0 mmol, 1.4 equiv) in dichloromethane (12 mL) was added triethylamine (0.9 mL, 6.5 mmol, 1.3 equiv), allylamine (0.45 mL, 6.0 mmol, 1.2 equiv) and 4-methoxybenzoic acid (760 mg, 5.0 mmol, 1.0 equiv) at 0 °C. After slowly warming to ambient temperature the reaction mixture was stirred for 23 h. After aqueous workup, the crude product was purified by column chromatography (cyclohexane : ethyl

acetate, 2:1). The product (835 mg, 4.4 mmol, 85%) was obtained as a colourless solid.

R*f*: 0.32 (cyclohexane : ethyl acetate, 1:2).

M.p.: 47 °C.

^{1}H NMR (400 MHz, $CDCl_3$, 298 K): δ [ppm] = 7.75 (m, 2H, H-C3), 6.91 (m, 2H, H-C2), 6.20 (br-s, 1H, NH), 5.93 (ddt, $^3J_{HH}$ = 17.1, 10.2, 5.7 Hz, 1H, H-C7), 5.25 (dq, $^3J_{HH}$ = 17.0 Hz, $^2J_{HH}$ = $^4J_{HH}$ = 1.5 Hz, 1H, H^Z-C8), 5.17 (dq, $^3J_{HH}$ = 10.2 Hz, $^2J_{HH}$ = $^4J_{HH}$ = 1.5 Hz, 1H, H^E-C8), 4.07 (td, $^3J_{HH}$ = 5.6 Hz, $^4J_{HH}$ = 1.5 Hz, 2H, H-C6), 3.84 (s, 3H, H-C9).

The analytical data are in agreement with literature values[4].

N-Allyl-4-nitrobenzamide (1c)[6]

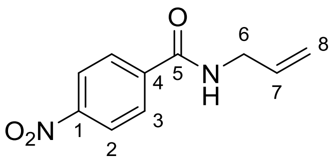

The compound was prepared according to a modified procedure by Niu and Song et al.[5]: 1-(3-Dimethylaminopropyl)-3-ethylcarbodiimide (1.1 mL, 6.5 mmol, 1.3 equiv) and 4-(dimethylamino)pyridine (855 mg, 7.0 mmol, 1.4 equiv) were dissolved in dichloromethane (12 mL) and cooled to 0 °C. 4-Nitrobenzoic acid (835 mg, 5.0 mmol, 1.0 equiv) and allylamine (0.5 mL, 6.0 mmol, 1.2 equiv) were added sequentially. The reaction mixture was stirred for 23 h after slowly warming to ambient temperature. The solution was diluted with ethyl acetate (10 mL), the organic phase was washed with aqueous HCl (10 mL, 1 M) and separated. The aqueous phase was extracted with ethyl acetate (3 × 10 mL) and the combined organic phases were dried over $MgSO_4$. The solvent was removed in vacuo and the residue was purified by column chromatography (cyclohexane : ethyl acetate, 2:1). The product (706 mg, 3.4 mmol, 68%) was obtained as a colourless solid.

M.p.: 114 °C.

^{1}H NMR (400 MHz, $CDCl_3$, CD_3OD-spike [3 drops], 298 K): δ [ppm] = 8.24 (m, 2H, H-C2), 7.94 (m, 2H, H-C3), 5.89 (ddt, $^3J_{HH}$ = 17.1 Hz, 10.2 Hz, 5.7 Hz, 1H, H-C7), 6.74 (br-s, 1H, NH), 5.23 (dq, $^3J_{HH}$ = 17.2 Hz, $^2J_{HH}$ = $^4J_{HH}$ = 1.5 Hz, 1H, H^Z-C8), 5.16

(dq, $^3J_{HH}$ = 10.2 Hz, $^2J_{HH}$ = $^4J_{HH}$ = 1.5 Hz, 1H, H^E-C8), 4.03 (dt, $^3J_{HH}$ = 5.8 Hz, $^4J_{HH}$ = 1.5 Hz, 2H, H-C6).

The analytical data are in agreement with literature values[6].

N-Allyl-4-(trifluoromethyl)benzamide (1d)

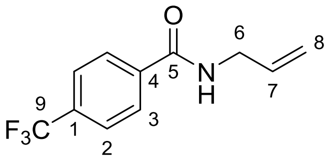

The compound was prepared according to the procedure for **1c**[5]. To a solution of 1-(3-dimethylaminopropyl)-3-ethylcarbodiimid hydrochloride (1.25 g, 6.5 mmol, 1.3 equiv) and 4-(dimethylamino)-pyridine (855 mg, 7.0 mmol, 1.4 equiv) in dichloromethane (12 mL) was added triethylamine (0.9 mL, 6.5 mmol, 1.3 equiv), allylamine (0.45 mL, 6.0 mmol, 1.2 equiv) and 4-(trifluoromethyl)benzoic acid (951 mg, 5.0 mmol, 1.0 equiv) at 0 °C. After slowly warming to ambient temperature the reaction mixture was stirred for 22 h. After aqueous workup, the crude product was purified by column chromatography (cyclohexane : ethyl acetate, 2:1). The product (1.04 g, 4.5 mmol, 91%) was obtained as a colourless crystalline solid.

R_f: 0.44 (cyclohexane : ethyl acetate, 2:1).

M.p.: 103 °C.

FT-IR ($\tilde{\nu}$ = cm^{-1}): 3284 (m), 2927 (w), 1652 (m), 1639 (s), 1578 (m), 1543 (w), 1509 (m), 1426 (m), 1408 (m), 1362 (m), 1326 (s), 1309 (s), 1266 (m), 1152 (s), 1110 (s), 1082 (s), 1066 (s), 1017 (s), 1031 (w), 999 (m), 983 (m), 964 (m), 921 (m), 858 (s), 771 (m), 698 (m), 674 (s).

^{1}H NMR (600 MHz, $CDCl_3$, 298 K): δ [ppm] = 7.88 (m, 2H, H-C3), 7.67 (m, 2H, H-C2), 6.44 (br-s, 1H, NH), 5.92 (ddt, $^3J_{HH}$ = 17.1, 10.2, 5.7 Hz, 1H, H-C7), 5.26 (dq, $^3J_{HH}$ = 17.1 Hz, $^2J_{HH}$ = $^4J_{HH}$ = 1.5 Hz, 1H, H^Z-C8), 5.20 (dq, $^3J_{HH}$ = 10.2 Hz, $^2J_{HH}$ = $^4J_{HH}$ = 1.5 Hz, 1H, H^E-C8), 4.08 (tt, $^3J_{HH}$ = 5.7 Hz, $^4J_{HH}$ = 1.5 Hz, 2H, H-C6).

^{13}C{^{1}H} NMR (151 MHz, $CDCl_3$, 298 K): δ [ppm] = 166.2 (C5), 137.9 (C4), 133.8 (C7), 133.4 (q, $^2J_{FC}$ = 32.6 Hz, C1), 127.6 (C3), 125.8 (q, $^4J_{FC}$ = 3.8 Hz, C2), 123.8 (q, $^1J_{FC}$ = 272.5 Hz, C9), 117.2 (C8), 42.7 (C6).

^{19}**F{^{1}H} NMR** (564 MHz, $CDCl_3$, 298 K): δ [ppm] = 166.2 (F-C9).

ESI-MS: (*m/z*) required $[C_{11}H_{10}NOF_3)Na]^+$ = 252.0607, (*m/z*) found: $[C_{11}H_{10}NOF_3)Na]^+$ 252.0606.

N-allyl-4-formylbenzamide (1e)[7]

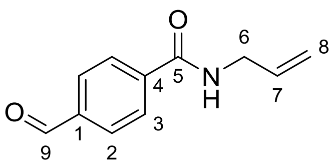

The compound was prepared according to the procedure for **1c**[5]. To a solution of 1-(3-dimethylaminopropyl)-3-ethylcarbodiimide hydrochloride (1.25 g, 6.5 mmol, 1.3 equiv) and 4-(dimethylamino)pyridine (855 mg, 7.0 mmol, 1.4 equiv) in dichloromethane was added triethylamine (0.9 mL, 6.5 mmol, 1.3 equiv), allylamine (0.45 mL, 6.0 mmol, 1.2 equiv) and 4-formylbenzoic acid (751 mg, 5.0 mmol, 1.0 equiv) at 0 °C. After slowly warming to ambient temperature the reaction mixture was stirred for 18 h. After aqueous workup, the crude product was purified by column chromatography (cyclohexane : ethyl acetate, 2:1). The product (715 mg, 3.8 mmol, 76%) was obtained as a colourless solid.

R$_f$: 0.40 (cyclohexane : ethyl acetate, 2:1).

M.p.: 81 °C.

^{1}**H NMR** (600 MHz, $CDCl_3$, 298 K): δ [ppm] = 10.05 (s, 1H, H-C9), 7.93 (m, 2H, H-C3), 7.91 (m, 2H, H-C2), 6.57 (br-s, 1H, NH), 5.92 (ddt, $^3J_{HH}$ = 17.2, 10.2, 5.7 Hz, 1H, H-C7), 5.25 (dq, $^3J_{HH}$ = 17.2 Hz, $^2J_{HH}$ = $^4J_{HH}$ = 1.6 Hz, 1H, H^Z-C8), 5.18 (dq, $^3J_{HH}$ = 10.2 Hz, $^2J_{HH}$ = $^4J_{HH}$ = 1.5 Hz, 1H, H^E-C8), 4.08 (tt, $^3J_{HH}$ = 5.7 Hz, $^4J_{HH}$ = 1.6 Hz, 2H, H-C6).

The analytical data are in agreement with literature values[7].

N-allyl-2-bromobenzamide (1f)[8]

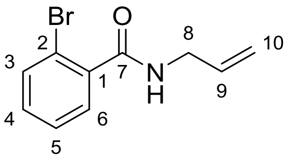

The compound was prepared according to the procedure for **1c**[5]. To a solution of 1-(3-dimethylaminopropyl)-3-ethylcarbodiimide hydrochloride (1.25 g, 6.5 mmol, 1.3 equiv) and 4-(dimethylamino)pyridine (855 mg, 7.0 mmol, 1.4 equiv) in dichloromethane (12 mL) was added triethylamine (0.9 mL, 6.5 mmol, 1.3 equiv),

allylamine (0.45 mL, 6.0 mmol, 1.2 equiv) and 2-bromobenzoic acid (1.01 g, 5.0 mmol, 1.0 equiv) at 0 °C. After slowly warming to ambient temperature the reaction mixture was stirred for 21 h. After aqueous workup, the crude product was purified by column chromatography (cyclohexane : ethyl acetate, 2:1). The product (902 mg, 3.8 mmol, 75%) was obtained as a colourless solid.

R$_f$: 0.28 (cyclohexane : ethyl acetate, 2:1).

M.p.: 90 °C.

^{1}H NMR (600 MHz, $CDCl_3$, 298 K): δ [ppm] = 7.57 (dd, $^3J_{HH}$ = 8.0 Hz, $^4J_{HH}$ = 1.1 Hz, 1H, H-C3), 7.52 (dd, $^3J_{HH}$ = 7.6 Hz, $^4J_{HH}$ = 1.8 Hz, 1H, H-C6), 7.34 (td, $^3J_{HH}$ = 7.6 Hz, $^4J_{HH}$ = 1.6 Hz, 1H, H-C5), 7.26 (ddd, $^3J_{HH}$ = 8.3, 7.4 Hz, $^4J_{HH}$ = 1.7 Hz, 1H, H-C4), 6.11 (br-s, 1H, NH), 5.94 (ddt, $^3J_{HH}$ = 17.1, 10.2, 5.7 Hz, 1H, H-C9), 5.31 (dq, $^3J_{HH}$ = 17.1 Hz, $^2J_{HH}$ = $^4J_{HH}$ = 1.6 Hz, 1H, H^Z-C10), 5.19 (dq, $^3J_{HH}$ = 10.3 Hz, $^2J_{HH}$ = $^4J_{HH}$ = 1.6 Hz, 1H, H^E-C10), 4.08 (tt, $^3J_{HH}$ = 5.7 Hz, $^4J_{HH}$ = 1.6 Hz, 2H, H-C8).

The analytical data are in agreement with literature values[8].

***N*-Allyl-2-aminobenzamide (S1)[9]**

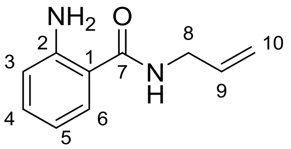

The compound was prepared according to the procedure for **1c**[5]. To a solution of 1-(3-dimethylaminopropyl)-3-ethylcarbodiimide hydrochloride (1.25 g, 6.5 mmol, 1.3 equiv) and 4-(dimethylamino)-pyridine (855 mg, 7.0 mmol, 1.4 equiv) in dichloromethane (12 mL) was added triethylamine (0.9 mL, 7.0 mmol, 1.3 equiv), allylamine (0.75 mL, 10.0 mmol, 2.0 equiv) and anthranilic acid (686 mg, 5.0 mmol, 1.0 equiv) in dichloromethane (12 mL) at 0 °C. After slowly warming to ambient temperature the reaction mixture was stirred for 23 h. After aqueous workup, the crude product was purified by column chromatography (cyclohexane : ethyl acetate, 4:1 to 2:1). The product (786 mg, 4.46 mmol, 89%) was obtained as a colourless solid.

R$_f$: 0.12 (cyclohexane : ethyl acetate, 4:1).

M.p.: 84 °C.

^{1}H NMR (600 MHz, $CDCl_3$, 298 K): δ [ppm] = 7.33 (dd, $^3J_{HH}$ = 7.9 Hz, $^4J_{HH}$ = 1.5 Hz, 1H, H-C6), 7.20 (ddd, $^3J_{HH}$ = 8.2, 7.2 Hz, $^4J_{HH}$ = 1.5 Hz, 1H, H-C4), 6.67 (dd, $^3J_{HH}$ =

8.2 Hz, ${}^{4}J_{HH}$ = 1.2 Hz, 1H, H-C3), 6.63 (ddd, ${}^{3}J_{HH}$ = 8.2, 7.9 Hz, ${}^{4}J_{HH}$ = 1.1 Hz, 1H, H-C5), 6.20 (br-s, 1H, NH), 5.92 (ddt, ${}^{3}J_{HH}$ = 17.2, 10.2, 5.6 Hz, 1H, H-C9), 5.49 (br-s, 2H, NH_2), 5.25 (dq, ${}^{3}J_{HH}$ = 17.2 Hz, ${}^{2}J_{HH}$ = ${}^{4}J_{HH}$ = 1.6 Hz, 1H, H^{Z}-C10), 5.17 (dq, ${}^{3}J_{HH}$ = 10.2 Hz, ${}^{2}J_{HH}$ = ${}^{4}J_{HH}$ = 1.6 Hz, 1H, H^{E}-C10), 4.03 (tt, ${}^{3}J_{HH}$ = 5.7 Hz, ${}^{4}J_{HH}$ = 1.6 Hz, 2H, H-C8).

The analytical data is in agreement with literature values[9].

2-acetamido-*N*-allylbenzamide (1g)

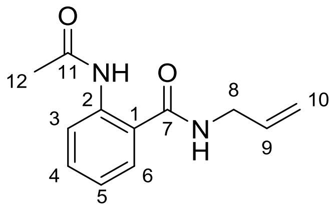

N-Allyl-2-aminobenzamide (**S1**) (176 mg, 1.0 mmol, 1.0 equiv) was dissolved in dichloromethane (1 mL) and acetic anhydride (1 mL, 10.5 mmol, 10.5 equiv) was added via syringe. The mixture was stirred at ambient temperature for 3 h before being poured into aqueous saturated $NaHCO_3$ solution (10 mL). The aqueous phase was extracted with dichloromethane (3 x 10 mL) and the combined organic phases were dried over $MgSO_4$. The solvent was removed in vacuo and the residue was purified by column chromatography (cyclohexane : ethyl acetate, 1:1). The product (206 mg, 0.94 mmol, 94%) was obtained as a colourless solid.

R_f: 0.40 (cyclohexane : ethyl acetate, 1:1).

M.p.: 126 °C.

FT-IR ($\tilde{\nu}$ = cm^{-1}): 3249 (m), 3037 (w), 1657 (s), 1630 (s), 1600 (s), 1532 (s), 1487 (s), 1434 (m), 1421 (m), 1366 (m), 1345 (m), 1303 (s), 1250 (s), 1161 (m), 1141 (w), 1115 (w), 1036 (m), 1016 (w), 992 (m), 974 (m), 955 (w), 924 (s), 883 (w), 802 (m), 758 (s), 652 (s).

^{1}H NMR (400 MHz, $CDCl_3$, 298 K): δ [ppm] = 11.00 (br-s, 1H, AcNH), 8.52 (dd, ${}^{3}J_{HH}$ = 8.4 Hz, ${}^{4}J_{HH}$ = 1.2 Hz, 1H, H-C3), 7.47 (dd, ${}^{3}J_{HH}$ = 7.8 Hz, ${}^{4}J_{HH}$ = 1.5 Hz, 1H, H-C6), 7.43 (ddd, ${}^{3}J_{HH}$ = 8.7, 7.4 Hz, ${}^{4}J_{HH}$ = 1.5 Hz, 1H, H-C4), 7.04 (ddd, ${}^{3}J_{HH}$ = 7.8, 7.4 Hz, ${}^{4}J_{HH}$ = 1.2 Hz, 1H, H-C5), 6.62 (br, 1H, NH), 5.93 (ddt, ${}^{3}J_{HH}$ = 17.1, 10.2, 5.6 Hz, 1H, H-C9), 5.28 (dq, ${}^{3}J_{HH}$ = 17.1 Hz, ${}^{2}J_{HH}$ = ${}^{4}J_{HH}$ = 1.6 Hz, 1H, H^{Z}-C10), 5.21 (dq, ${}^{3}J_{HH}$ = 10.2 Hz, ${}^{2}J_{HH}$ = ${}^{4}J_{HH}$ = 1.6 Hz, 1H, H^{E}-C10), 4.04 (tt, ${}^{3}J_{HH}$ = 5.7 Hz, ${}^{4}J_{HH}$ = 1.6 Hz, 2H, H-C8), 2.17 (s, 3H, H-C12).

$^{13}C\{^{1}H\}$ **NMR** (101 MHz, $CDCl_3$, 298 K): δ [ppm] = 169.2 (C11), 169.0 (C7), 139.6 (C2), 133.6 (C9), 132.6 (C4), 126.6 (C6), 122.8 (C5), 121.6 (C3), 120.4 (C1), 117.2 (C10), 42.4 (C8), 25.4 (C12).

ESI-MS: (*m/z*) required $[C_{12}H_{14}N_2O_2)Na]^+$ = 241.0947, (*m/z*) found: $[C_{12}H_{14}N_2O_2)Na]^+$ 241.0954.

N-allyl-5-bromo-2-chlorobenzamide (1h)[6]

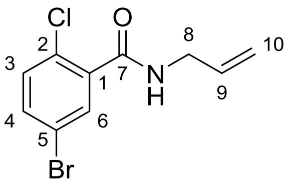

This compound was prepared according to the procedure for **1m**[12]. 5-Bromo-2-chlorobenzoic acid (471 mg, 2 mmol, 1.0 equiv) was dissolved in thionyl chloride (7.3 mL, 100 mmol, 50 equiv). After stirring at reflux, the volatiles were removed in vacuo. The residue was dissolved in dichloromethane (4 mL), allylamine (0.75 mL, 10 mmol, 5 equiv) was added and the mixture was stirred for 2 h at ambient temperature. After aqueous workup, the crude product was purified by recrystallization from dichloromethane and *n*-pentane. The product (455 mg, 1.7 mmol, 83%) was obtained as a white solid.

M.p.: 121 °C.

^{1}H **NMR** (300 MHz, $CDCl_3$, 298 K): δ [ppm] = 7.80 (d, $^4J_{HH}$ = 2.4 Hz, 1H, H-C6), 7.48 (dd, $^3J_{HH}$ = 8.6 Hz, $^4J_{HH}$ = 2.4 Hz, 1H, H-C4), 7.27 (d, $^3J_{HH}$ = 7.9 Hz, 1H, H-C3), 6.27 (br-s, 1H, NH), 5.93 (ddt, $^3J_{HH}$ = 17.2, 10.2, 5.6 Hz, 1H, H-C9), 5.30 (dq, $^3J_{HH}$ = 17.2 Hz, $^2J_{HH}$ = $^4J_{HH}$ = 1.6 Hz, 1H, H^Z-C10), 5.21 (dq, $^3J_{HH}$ = 10.3 Hz, $^2J_{HH}$ = $^4J_{HH}$ = 1.4 Hz, 1H, H^E-C10), 4.09 (tt, $^3J_{HH}$ = 5.7 Hz, $^4J_{HH}$ = 1.6 Hz, 2H, H-C8).

The analytical data are in agreement with literature values[6].

N-allyl-2-hydroxy-5-nitrobenzamide (1i)

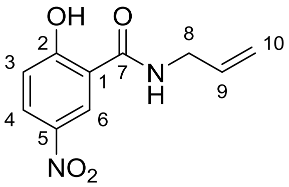

This compound was prepared according to the procedure for **1m**[12]. 2-Hydroxy-5-nitrobenzoic acid (367 mg, 2 mmol, 1.0 equiv) was dissolved in thionyl chloride (7.3 mL, 100 mmol, 50 equiv). After stirring at reflux, the volatiles were removed in vacuo. The residue was dissolved in dichloromethane (4 mL), allylamine (0.75 mL,

10 mmol, 5 equiv) was added and the mixture was stirred for 2 h at ambient temperature. After aqueous workup, the crude product was purified by recrystallization from dichloromethane and *n*-pentane. The product (323 mg, 1.5 mmol, 73%) was obtained as a white solid.

M.p.: 109 °C.

FT-IR ($\tilde{\nu}$ = cm^{-1}): 3383 (m), 3094 (w), 1641 (m), 1587 (m), 1547 (m), 1512 (m), 1477 (m), 1418 (m), 1376 (m), 1332 (s), 1297 (s), 1271 (s), 1228 (m), 1151 (s), 1090 (m), 995 (m), 923 (m), 915 (m), 848 (s), 810 (m), 747 (s), 711 (m), 697 (m).

^{1}H NMR (600 MHz, $CDCl_3$, 298 K): δ [ppm] = 8.44 (d, $^4J_{HH}$ = 2.6 Hz, 1H, H-C6), 8.28 (dd, $^3J_{HH}$ = 9.2 Hz, $^4J_{HH}$ = 2.6 Hz, 1H, H-C4), 7.08 (d, $^3J_{HH}$ = 9.2 Hz, 1H, H-C3), 6.71 (br-s, 1H, NH), 5.95 (ddt, $^3J_{HH}$ = 17.1, 10.2, 5.9 Hz, 1H, H-C9), 5.33 (dq, $^3J_{HH}$ = 17.1 Hz, $^2J_{HH}$ = $^4J_{HH}$ = 1.5 Hz, 1H, H^Z-C10), 5.28 (dq, $^3J_{HH}$ = 10.2 Hz, $^2J_{HH}$ = $^4J_{HH}$ = 1.3 Hz, 1H, H^E-C10), 4.13 (tt, $^3J_{HH}$ = 5.8 Hz, $^2J_{HH}$ =1.5 Hz, 2H, H-C8).

^{13}C{^{1}H} NMR (151 MHz, $CDCl_3$, 298 K): δ [ppm] = 168.5 (C7), 167.4 (C2), 139.4 (C5), 132.8 (C9), 129.5 (C4), 122.4 (C6), 119.7 (C3), 118.3 (C10), 113.7 (C1), 42.5 (C8).

ESI-MS: (*m/z*) required: $[C_{10}H_{10}N_2O_4)Na]^+$ = 245.0533, (*m/z*) found: $[C_{10}H_{10}N_2O_4)Na]^+$ 245.0533.

***N*-allyl-2,3,4,5,6-pentafluorobenzamide (1j)**

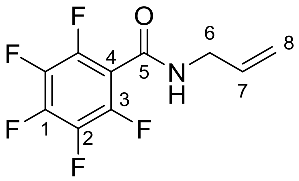

The compound was prepared according to the procedure for **1c**[5]. To a solution of 1-(3-dimethylaminopropyl)-3-ethylcarbodiimide hydrochloride (623 mg, 3.3 mmol, 1.3 equiv) and 4-(dimethylamino)-pyridine (428 mg, 3.5 mmol, 1.4 equiv) in dichloromethane (12 mL) was added triethylamine (0.45 mL, 3.3 mmol, 1.3 equiv), allylamine (0.23 mL, 3.0 mmol, 1.2 equiv) and 2,3,4,5,6-pentafluorobenzoic acid (530 mg, 2.5 mmol, 1.0 equiv) at 0 °C. After slowly warming to ambient temperature the reaction mixture was stirred for 23 h. After aqueous workup, the crude product was purified by column chromatography (cyclohexane : ethyl acetate, 7:1). The product (104 mg, 0.4 mmol, 17%) was obtained as a colourless solid.

R_f: 0.33 (cyclohexane : ethyl acetate, 4:1).

M.p.: 69 °C.

FT-IR ($\tilde{\nu}$ = cm^{-1}): 3240 (w), 3069 (w), 2913 (w), 2869 (w), 1656 (s), 1637 (s), 1571 (m), 1495 (s), 1446 (m), 1425 (m), 1410 (m), 1359 (m), 1328 (s), 1295 (w), 1270 (m), 1244 (s), 1131 (w), 1095 (s), 1026 (m), 999 (s), 985 (s), 938 (s), 905 (s), 757 (s), 738 (s), 686 (m).

^{1}H NMR (400 MHz, $CDCl_3$, 298 K): δ [ppm] = 6.15 (br-s, 1H, NH), 5.90 (ddt, $^3J_{HH}$ = 17.2, 10.3, 5.5 Hz, 1H, H-C7), 5.29 (dq, $^3J_{HH}$ = 17.2 Hz, $^2J_{HH}$ = $^4J_{HH}$ = 1.7 Hz, 1H, H^Z-C8), 5.22 (dq, $^3J_{HH}$ = 10.3 Hz, $^2J_{HH}$ = $^4J_{HH}$ = 1.4 Hz, 1H, H^E-C8), 4.08 (tt, $^3J_{HH}$ = 5.5 Hz, $^4J_{HH}$ = 1.6 Hz, 2H, H-C6).

^{13}C{^{1}H} NMR (101 MHz, $CDCl_3$, 298 K): δ [ppm] = 157.4 (C5), 144.4 (m, C1), 142.6 (m, C3), 137.9 (m, C2), 132.9 (C7), 117.4 (C8), 111.4 (C4), 42.7 (C6).

^{19}F NMR (282 MHz, $CDCl_3$, 298 K): δ [ppm] = -140.3 (m, 2F, F-C3), -159.6 (tt, $^3J_{FF}$ = 20.8 Hz, $^4J_{FF}$ = 3.0 Hz, 1F, F-C1), -160.0 (td, $^3J_{FF}$ = 20.8 Hz, $^4J_{FF}$ = 6.6 Hz, 2F, F-C2).

ESI-MS: (*m/z*) required: $[(C_{10}H_6F_5NO)Na]^+$ = 274.0262; (*m/z*) found: $[(C_{10}H_6F_5NO)Na]^+$ = 274.0262.

2-(But-3-en-1-yl)isoindoline-1,3-dione (S2)[10]

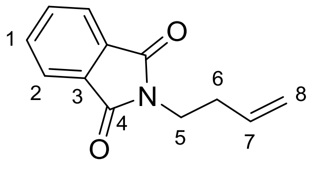

The compound was prepared according to a modified procedure by *A. P.* Dobbs et al.[10]. 3-Buten-1-ol (0.6 mL, 6.9 mmol, 1.0 equiv) was dissolved in THF (11 mL) and triphenylphosphine (1.99 g, 7.6 mmol, 1.1 equiv) and phthalimide (1.12 g, 7.6 mmol, 1.1 equiv) were added. The mixture was cooled to 0 °C before diisopropyl azodicarboxylate (1.5 mL, 7.6 mmol, 1.1 equiv) was added dropwise *via* syringe. After slowly warming to ambient temperature the reaction mixture was stirred over 22 h. The solvent was removed in vacuo and the solid residue was purified by column chromatography (cyclohexane : ethyl acetate, 10:1). The product (1.18 g, 5.9 mmol, 85%) was obtained as a colourless solid.

R_f: 0.21 (cyclohexane : ethyl acetate, 10:1).

M.p.: 53 °C.

^{1}H NMR (400 MHz, $CDCl_3$, 298 K): δ[ppm] = 7.83 (m, 2H, H-C2), 7.71 (m, 2H, H-C1), 5.79 (ddt, $^3J_{HH}$ = 17.1, 10.2, 6.9 Hz, 1H, H-C7), 5.07 (dq, $^3J_{HH}$ = 17.1 Hz, $^2J_{HH}$ = $^4J_{HH}$ = 1.7 Hz, 1H, H^Z-C8), 5.02 (dq, $^3J_{HH}$ = 10.2 Hz, $^2J_{HH}$ = $^4J_{HH}$ = 1.7 Hz, 1H, H^E-C8), 3.77 (t, $^3J_{HH}$ = 7.1 Hz, 2H, H-C5), 2.45 (qt, $^3J_{HH}$ = 7.0 Hz, $^4J_{HH}$ = 1.7 Hz, 2H, H-C6).

The analytical data are in agreement with literature values[10].

***N*-(but-3-en-1-yl)benzamide (1k)[10]**

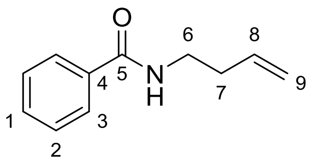

The compound was prepared according to a modified procedure by *P. A.* Dobbs et al.[10]. 2-(But-3-en-1-yl)isoindolin-1,3-dion (**S2**) (1.10 g, 5.0 mmol, 1.0 equiv) was dissolved in ethanol (25 mL) and a solution of hydrazine (10.0 mL, 10.0 mmol, 2.0 equiv 1 M in acetonitrile) was added dropwise. The reaction mixture was heated to 50 °C and stirred for 2 h before being allowed to cool to ambient temperature. HCl (5 mL, conc.) was added and the mixture was stirred for 10 min. The resulting precipitate was filtered off and the solvent was removed in vacuo. The residue was taken up in water (10 mL), the suspension was once more filtered and the solvent was removed *in vacuo*. The crude product but-3-en-1-amine (538 mg, 5.0 mmol, 1.2 equiv) was dissolved in dichloromethane (6 mL) and triethylamine (0.7 mL, 5.0 mmol, 1.2 equiv). This solution was added to a solution of 1-(3-dimethlyaminopropyl)-3-ethylcarbodiimide (1.0 mL, 5.4 mmol, 1.3 equiv) and 4-(dimethylamino)pyridine (718 mg, 5.9 mmol, 1.4 equiv) in dichloromethane (6 mL) at 0 °C. After stirring for 10 min, benzoic acid (513 mg, 4.2 mmol, 1.0 equiv) was added and after slowly warming to ambient temperature the reaction mixture stirred for 2 days. The reaction mixture was subsequently diluted with ethyl acetate (15 mL) and the organic phase was washed with aqueous HCl (10 mL, 1 M) and separated. The aqueous phase was extracted with ethyl acetate (3 × 10 mL) and the combined organic phases were dried over $MgSO_4$. The solvent was removed in vacuo and the residue was purified by column chromatography (cyclohexane : ethyl acetate, 4:1). The product (532 mg, 3.0 mmol, 72%) was obtained over two steps as a colourless oil.

R_f: 0.38 (cyclohexane : ethyl acetate, 4:1).

^{1}H NMR (400 MHz, $CDCl_3$, 298 K): δ [ppm] = 7.77–7.72 (m, 2H, H-C3), 7.52–7.46 (m, 1H, H-C1), 7.46–7.40 (m, 2H, H-C2), 6.18 (br-s, 1H, NH), 5.84 (ddt, $^3J_{HH}$ = 17.1, 10.2, 6.9 Hz, 1H, H-C8), 5.19–5.11 (m, 2H, H-C9), 3.54 (td, $^3J_{HH}$ = 6.7, 5.2 Hz, 2H, H-C6), 2.39 (qt, $^3J_{HH}$ = 6.8 Hz, $^4J_{HH}$ = 1.5 Hz, 2H, H-C7).

The analytical data are in agreement with literature values[10].

2-(Pent-4-en-1-yl)isoindoline-1,3-dione (S3)[11]

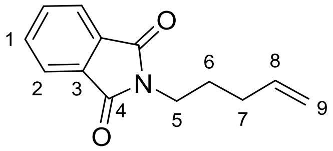

The compound was prepared according to the procedure for **S2**[10]. To a solution of 4-penten-1-ol (0.3 mL, 2.8 mmol, 1.0 equiv) in THF (6 mL) was added triphenylphosphine (808 mg, 3.1 mmol, 1.1 equiv), phthalimide (453 mg, 3.1 mmol, 1.1 equiv) and diisopropyl azodicarboxylate (0.6 mL, 3.1 mmol, 1.1 equiv) in at 0 °C. The reaction mixture was stirred over 22 h after slowly warming to ambient temperature. The solvent was removed in vacuo and the solid residue was purified by column chromatography (cyclohexane : ethyl acetate, 10:1). The product (486 mg, 2.3 mmol, 80%) was obtained as a colourless solid.

R_f: 0.23 (cyclohexane : ethyl acetate, 10:1).

M.p.: 34 °C.

^{1}H NMR (400 MHz, $CDCl_3$, 298 K): δ [ppm] = 7.83 (m, 2H, H-C2), 7.71 (m, 2H, H-C1), 5.81 (ddt, $^3J_{HH}$ = 16.9, 10.2, 6.6 Hz, 1H, H-C8), 5.05 (dq, $^3J_{HH}$ = 17.1 Hz, $^2J_{HH}$ = $^4J_{HH}$ = 1.7 Hz, 1H, H^Z-C9), 4.97 (dq, $^3J_{HH}$ = 10.2 Hz, $^2J_{HH}$ = $^4J_{HH}$ = 1.5 Hz, 1H, H^E-C9), 3.71 (t, $^3J_{HH}$ = 7.3 Hz, 2H, H-C5), 2.17–2.06 (m, 2H, H-C7), 1.78 (p, $^3J_{HH}$ = 7.5 Hz, 2H, H-C6).

The analytical data are in agreement with literature values[11].

N-(pent-4-en-1yl)benzamide (1l)[12]

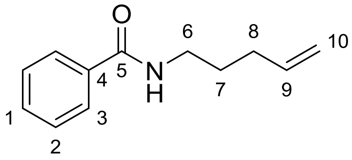

The compound was prepared according to the procedure for **1k**[10]. To a solution of 2-(pent-4-en-1-yl)isoindoline-1,3-dione (**S3**) (430 mg, 2.0 mmol, 1.0 equiv) in ethanol (25

mL) was added hydrazine (4.0 mL, 4.0 mmol, 2.0 equiv 1 M in acetonitrile) and the mixture was stirred at 50 °C for 2 h. The crude intermediate but-3-en-1-amine (244 mg, 2.0 mmol, 1.0 equiv) was added to a solution of triethylamine (0.6 mL, 4.0 mmol, 2.0 equiv) and benzoylchloride (0.2 mL, 2.1 mmol, 1.05 equiv) in dichloromethane (3 mL) at 0 °C. After slowly warming to ambient temperature the mixture was stirred for 16 h. After aqueous workup, the crude product was purified by column chromatography (cyclohexane : ethyl acetate, 4:1). The product (246 mg, 1.3 mmol, 65%) was obtained as a colourless oil.

R$_f$: 0.19 (cyclohexane : ethyl acetate, 4:1).

^{1}H NMR (300 MHz, $CDCl_3$, 298 K): δ [ppm] = 8.18–7.31 (m, 5H, H-C1, H-C2, H-C3), 6.13 (br-s, 1H, NH), 5.84 (ddt, $^3J_{HH}$ = 16.9, 10.0, 6.6 Hz, 1H, H-C9), 5.07 (dd, $^2J_{HH}$ = 1.8 Hz, $^3J_{HH}$ = 17.0 Hz, 1H, H^Z-C10), 5.01 (dd, $^2J_{HH}$ = 1.5 Hz, $^3J_{HH}$ = 10.0 Hz, 1H, H^E-C10), 3.55–3.38 (m, 2H, H-C6), 2.17 (td, $^3J_{HH}$ = 7.3, 6.6 Hz, 2H, H-C8), 1.73 (p, $^3J_{HH}$ = 7.3 Hz, 2H, H-C7).

The analytical data are in agreement with literature values[12].

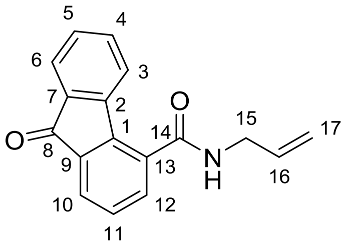

_N_-Allyl-9-oxo-9_H_-fluorene-4-carboxamide (1m)

This compound was prepared according to a modified procedure by Cui et al.[12] A 25 mL round bottom flask was charged with 9-oxofluorene-4-carboxylic acid (673 mg, 3 mmol, 1.0 equiv) and thionyl chloride (11 mL, 150 mmol, 50.0 equiv). The stirred mixture was heated to reflux for 1 h after which the stirring was continued for 14 h at ambient temperature. The solvent was removed in vacuo and the residue was dissolved in dichloromethane (6 mL). The solution was cooled to 0° C, allylamine (1.1 mL, 15 mmol, 5.0 equiv) was added dropwise and the mixture was stirred for 2 h at 0° C. The mixture was then poured into an aqueous solution of HCl (15 mL, 1M) and extracted with dichloromethane (3 × 10 mL). The combined organic phases were dried over $MgSO_4$ and the solvent was removed in vacuo. The residue was then purified by column chromatography on silica gel (cyclohexane : ethyl acetate, 1:2). The product (432 mg, 1.6 mmol, 53%) was obtained as a yellow solid.

R_f: 0.1 (cyclohexane : ethyl acetate, 1:2).

M.p.: 87 °C.

FT-IR ($\tilde{\nu}$ = cm^{-1}): 3237 (m), 3072 (m), 2923 (w), 2863 (w), 1693 (s), 1641 (m), 1604 (m) 1567 (s), 1472 (m), 1448 (m), 1418 (m), 1366 (m), 1339 (m), 1297 (m), 1283 (m), 1261 (m), 1193 (m), 1182 (m), 1156 (m), 1134 (m), 1100 (m), 1074 (m), 1004 (m), 920 (s), 849 (m), 830 (m), 808 (m), 772 (m), 744 (m), 737 (m), 676 (s), 660 (m).

^{1}H NMR (400 MHz, $CDCl_3$, 298 K): δ [ppm] = 10.21 (br-s, 1H, NH), 8.23 (dd, $^3J_{HH}$ = 6.6 Hz, $^4J_{HH}$ = 2.5 Hz, 1H, H-C12), 7.61–7.58 (m, 1H, H-C6), 7.57–7.53 (m, 2H, H-C10, H-C11), 7.49 (td, $^3J_{HH}$ = 7.3 Hz, $^4J_{HH}$ = 1.1 Hz, 1H, H-C4), 7.44 (dt, $^3J_{HH}$ = 7.5 Hz, $^4J_{HH}$ = 1.1 Hz, 1H, H-C3), 7.27 (td, $^3J_{HH}$ = 7.3 Hz, $^4J_{HH}$ = 1.3 Hz, 1H, H-C5), 6.03 (ddt, $^3J_{HH}$ = 17.2, 10.3, 5.6 Hz, 1H, H-C16), 5.36 (dq, $^3J_{HH}$ = 17.1 Hz, $^2J_{HH}$ = $^4J_{HH}$ = 1.6 Hz, 1H, H^Z-C17), 5.22 (dq, $^3J_{HH}$ = 10.3 Hz, $^2J_{HH}$ = $^4J_{HH}$ = 1.5 Hz, 1H, H^E-C17), 4.15 (tt, $^3J_{HH}$ = 5.5 Hz, $^4J_{HH}$ = 1.6 Hz, 2H, H-C15).

^{13}C{^{1}H} NMR (101 MHz, $CDCl_3$, 298 K): δ [ppm] = 195.9 (C8), 163.9 (C14), 145.7 (C9), 143.6 (C7), 135.9 (C4), 135.3 (C10), 134.2 (C16), 134.1 (C1), 132.9 (C2), 132.8 (C12), 130.2 (C13), 129.7 (C5), 125.2 (C6), 123.1 (C11), 120.2 (C3), 116.5 (C17), 42.8 (C15).

ESI-MS: (*m/z*) required $[C_{17}H_{13}NO_2)Na]^+$ = 286.0838, (*m/z*) found: $[C_{17}H_{13}NO_2)Na]^+$ 286.0839.

***N*-allylfuran-2-carboxamide (1n)[13]**

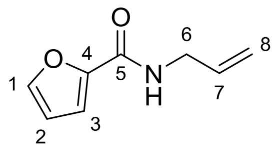

This compound was prepared according to a modified procedure by Fürstner et al.[13]. Furoyl chloride (0.25 mL, 2.50 mmol, 1.00 equiv) was added dropwise at 0 °C to a solution of allylamine (0.23 mL, 3.00 mmol, 1.20 equiv), triethylamine (0.52 mL, 3.75 mmol, 1.50 equiv) and 4-(dimethylamino)pyridine (16 mg, 0.13 mmol, 0.05 equiv) in dichloromethane (7.5 mL) and the reaction mixture was stirred for 3 h at ambient temperature. The mixture was diluted with dichloromethane (15 mL) and was then washed with an aqueous solution of HCl (10 mL, 1 M). The organic phase was dried over $MgSO_4$ and the solvent was removed in vacuo. The residue was separated via column chromatography on silica gel (cyclohexane : ethyl acetate, 4:1

to 2:1) affording the desired product as slightly yellow liquid (333 mg, 2.20 mmol, 88%).

R$_f$: 0.26 (cyclohexane : ethyl acetate, 2:1).

^{1}H NMR (400 MHz, $CDCl_3$, 298 K): δ [ppm] = 7.46 (dd, $^3J_{HH}$ = 1.8, 0.9 Hz, 1H, H-C1), 7.14 (dd, $^3J_{HH}$ = 3.4, 0.9 Hz, 1H, H-C3), 6.52 (dd, $^3J_{HH}$ = 3.5, 1.8 Hz, 1H, H-C2), 6.48 (br-s, 1H, NH), 5.93 (ddt, $^3J_{HH}$ = 17.3, 10.3, 5.6 Hz, 1H, H-C7), 5.28 (dq, $^3J_{HH}$ = 17.3, $^2J_{HH}$ = $^4J_{HH}$ = 1.6 Hz, 1H, H^Z-C8), 5.20 (dq, $^3J_{HH}$ = 10.2, $^2J_{HH}$ = $^4J_{HH}$ = 1.4 Hz, 1H, H^E-C8), 4.08 (tt, $^3J_{HH}$ = 5.8 Hz, $^4J_{HH}$ = 1.6 Hz, 2H, H-C6).

The analytical data are in agreement with literature values[13].

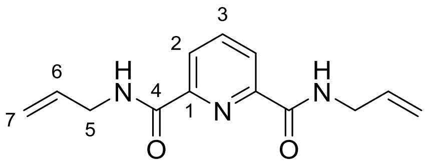

_N,N_-Ddiallylpyridine-2,6-dicarboxamide (1o)[14]

The compound was prepared according to the procedure for **1c**[5]. To a solution of 1-(3-dimethylaminopropyl)-3-ethylcarbodiimide hydro-chloride (1.25 g, 6.5 mmol, 2.6 equiv) and 4-(dimethylamino)pyridine (855 mg, 7.0 mmol, 2.8 equiv) in dichloromethane (12 mL) was added triethylamine (0.9 mL, 6.5 mmol, 2.6 equiv), allylamine (0.45 mL, 6.0 mmol, 2.4 equiv) and 2,6-pyridinedicarboxylic acid (418 mg, 2.5 mmol, 1.0 equiv) at 0 °C. After slowly warming to ambient temperature the reaction mixture was stirred for 3 d. After aqueous workup, the crude product was purified by column chromatography (cyclohexane : ethyl acetate, 1:1). The product (352 mg, 1.4 mmol, 57%) was obtained as a colourless solid.

R$_f$: 0.23 (cyclohexane : ethyl acetate, 1:1).

M.p.: 118 °C.

^{1}H NMR (300 MHz, $CDCl_3$, 298 K): δ [ppm] = 8.37 (d, $^3J_{HH}$ = 7.8 Hz, 2H, H-C2), 8.08 (t, $^3J_{HH}$ = 5.8 Hz, 2H, NH), 8.03 (t, $^3J_{HH}$ = 7.8 Hz, 1H, H-C3), 5.91 (ddt, $^3J_{HH}$ = 17.1, 10.2, 5.6 Hz, 2H, H-C6), 5.23 (dq, $^3J_{HH}$ = 17.1 Hz, $^2J_{HH}$ = $^4J_{HH}$ = 1.6 Hz, 2H, H^Z-C7), 5.13 (dq, $^3J_{HH}$ = 10.2 Hz, $^2J_{HH}$ = $^4J_{HH}$ = 1.6 Hz, 2H, H^E-C7), 4.10 (tt, $^3J_{HH}$ = 5.8 Hz, $^4J_{HH}$ = 1.6 Hz, 4H, H-C5).

The analytical data are in agreement with literature values[14].

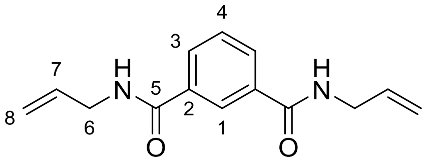

***N*,*N*-Diallylisophthalamide (1p)[15]**

This compound was prepared according to the procedure for **1c**[5]. To a solution of 1-(3-dimethylaminopropyl)-3-ethylcarbodiimide hydrochloride (1.50 g, 7.8 mmol, 2.6 equiv) and 4-(dimethylamino)-pyridine (1.03 g, 8.4 mmol, 2.8 equiv) in dichloromethane (18 mL) was added triethylamine (1.1 mL, 7.8 mmol, 2.6 equiv), allylamine (0.9 mL, 12.0 mmol, 4.0 equiv) and 1,3-benzenedicarboxylic acid (498 mg, 3.0 mmol, 1.0 equiv) at 0 °C. After slowly warming to ambient temperature the reaction mixture was stirred for 3 d. After aqueous workup, the crude product was purified by column chromatography (cyclohexane : ethyl acetate, 1:1). The resulting solid was recrystallized from dichloromethane and *n*-pentane and the product (198 mg, 0.8 mmol, 27%) was obtained as a colourless crystalline solid.

R_f: 0.22 (cyclohexane : ethyl acetate, 1:1).

M.p.: 114 °C.

^{1}H NMR (300 MHz, $CDCl_3$, 298 K): δ [ppm] = 8.23 (t, $^4J_{HH}$ = 1.9 Hz, 1H, H-C1), 7.93 (dd, $^3J_{HH}$ = 7.7 Hz, $^4J_{HH}$ = 1.9 Hz, 2H, H-C3), 7.50 (t, $^3J_{HH}$ = 7.8 Hz, 1H, H-C4), 6.53 (t, $^3J_{HH}$ = 5.6 Hz, 2H, NH), 5.91 (ddt, $^3J_{HH}$ = 17.2, 10.2, 5.7 Hz, 2H, H-C7), 5.25 (dq, $^3J_{HH}$ = 17.2 Hz, $^2J_{HH}$ = $^4J_{HH}$ = 1.6 Hz, 2H, H^Z-C8), 5.18 (dq, $^3J_{HH}$ = 10.2 Hz, $^2J_{HH}$ = $^4J_{HH}$ = 1.6 Hz, 2H, H^E-C8), 4.07 (tt, $^3J_{HH}$ = 5.8 Hz, $^4J_{HH}$ = 1.6 Hz, 4H, H-C6).

The analytical data are in agreement with literature values[15].

Benzyl 4-(allylamino)-4-oxobutanoate (1q)

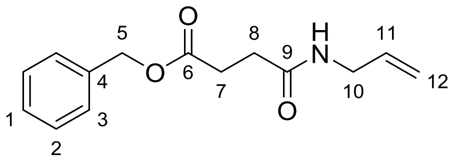

To a solution of allylamine (0.8 mL, 10.5 mmol, 2.1 equiv) in THF (5 mL) at 0 °C was added succinic anhydride (500 mg, 5.0 mmol, 1.0 equiv) in three portions. The reaction mixture was stirred for 3 h after slowly warming to ambient temperature. The volatile substances were

evaporated in vacuo and the residue was dissolved in THF (5 mL). Benzyl bromide (1.5 mL, 12.5 mmol, 2.5 equiv) and triethylamine (0.8 mL, 6.0 mmol, 1.2 equiv) were added to the solution and the reaction mixture was refluxed for 8 h before being allowed to cool to ambient temperature. The mixture was poured into aqueous HCl (1 M) and the aqueous phase was extracted with dichloromethane (3 × 20 mL). The combined organic phases were dried over $MgSO_4$ and the solvent was removed in vacuo. The residue was purified by column chromatography (cyclohexane : ethyl acetate, 1:1). The product (430 mg, 1.7 mmol, 34%) was obtained as a colourless solid.

R_f: 0.29 (cyclohexane : ethyl acetate, 1:1).

M.p.: 43 °C.

FT-IR ($\tilde{\nu}$ = cm^{-1}): 3246 (w), 3066 (w), 2931 (w), 1729 (s), 1626 (m), 1558 (s), 1656 (m), 1496 (w), 1456 (m), 1427 (m), 1404 (m), 1385 (m), 1355 (s), 1267 (m), 1247 (m), 1219 (w), 1155 (s), 1038 (m), 992 (m), 953 (m), 922 (s), 874 (w), 841 (w), 747 (s), 697 (s), 673 (m).

^{1}H NMR (600 MHz, $CDCl_3$, 298 K): δ [ppm] = 7.37–7.30 (m, 5H, H-C1, H-C2, H-C3), 5.82 (t, $^3J_{HH}$ = 5.6 Hz, 1H, NH), 5.80 (ddt, $^3J_{HH}$ = 17.1, 10.2, 5.6 Hz, 1H, H-C11), 5.17 (dq, $^3J_{HH}$ = 17.1 Hz, $^2J_{HH}$ = $^4J_{HH}$ = 1.6 Hz, 1H, H^Z-C12), 5.12 (s, 2H, H-C5), 3.11 (dq, $^3J_{HH}$ = 10.4 Hz, $^2J_{HH}$ = $^4J_{HH}$ = 1.4 Hz, 1H, H^E-C12), 3.86 (tt, $^3J_{HH}$ = 5.7 Hz, $^4J_{HH}$ = 1.6 Hz, 2H, H-C10), 2.73 (t, $^3J_{HH}$ = 6.8 Hz, 2H, H-C7), 2.50 (t, $^3J_{HH}$ = 6.8 Hz, 2H, H-C8).

$^{13}C\{^1H\}$ NMR (101 MHz, $CDCl_3$, 298 K): δ [ppm] = 172.9 (C9), 171.2 (C6), 135.8 (C4), 134.7 (C11), 128.7 (C2), 128.4 (C1), 128.3 (C3), 116.5 (C12), 66.7 (C5), 42.7 (C10), 31.1 (C8), 29.7 (C7).

ESI-MS: (*m/z*) required: $[(C_{14}H_{17}FNO_3)Na]^+$ = 270.1101; (*m/z*) found: $[(C_{14}H_{17}FNO_3)Na]^+$ = 270.1106.

(*S*)-*N*-Allyl-2-hydroxy-3-phenylpropanamide (S4)

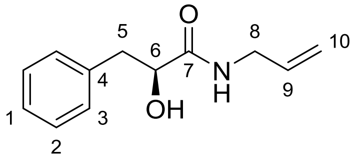

(*S*)-2-Hydroxy-3-phenylpropanoate (361 mg, 2.0 mmol, 1.0 equiv) was dissolved in ethanol (7.5 mL). Allylamine (4.5 mL, 60 mmol, 30 equiv) was added dropwise and the reaction mixture was stirred for 8 h at ambient temperature. The volatile substances were removed in vacuo and the residue was purified by column chromatography (cyclohexane : ethyl acetate, 1:1). The product (245 mg, 1.2 mmol, 60%) was obtained as a colourless oil.

R_f: 0.30 (cyclohexane : ethyl acetate, 1:1).

ORD (CH_2Cl_2, c 1.00): $[\boldsymbol{\alpha}]_{\boldsymbol{D}}^{\mathbf{25}}$ = -63.5°.

FT-IR ($\tilde{\nu}$ = cm^{-1}): 3315 (w), 3086 (w), 3030 (w), 2923 (w), 1640 (m), 1527 (m), 1497 (m), 1455 (w), 1419 (w), 1334 (w), 1274 (w) 1188 (w), 1085 (m), 1031 (w), 989 (w), 922 (m), 864 (w), 814 (w), 743 (m), 698 (s).

^{1}H NMR (400 MHz, $CDCl_3$, 298 K): δ [ppm] = 7.35–7.22 (m, 5H, H-C1, H-C2, H-C3), 6.75 (br-t, $^3J_{HH}$ = 6.0 Hz, 1H, NH), 5.77 (ddt, $^3J_{HH}$ = 17.4, 10.0, 5.6 Hz, 1H, H-C9), 5.15–5.12 (m, 1H, H^a-C10), 5.11–5.08 (m, 1H, H^b-C10), 4.29 (ddd, $^3J_{HH}$ = 8.5, 4.9, 3.9 Hz, 1H, H-C6), 3.92–3.77 (m, 2H, H-C8), 3.30 (d, $^3J_{HH}$ = 4.9 Hz, 1H, OH), 3.22 (dd, $^2J_{HH}$ = 13.9, $^3J_{HH}$ = 3.9 Hz, 1H, H^a-C5), 2.88 (dd, $^2J_{HH}$ = 13.9 Hz, $^3J_{HH}$ = 8.3 Hz, 1H, H^b-C5).

^{13}C{^{1}H} NMR (101 MHz, $CDCl_3$, 298 K): δ [ppm] = 172.9 (C7), 137.1 (C4), 133.8 (C9), 129.7 (C3), 128.7 (C2), 127.0 (C1), 116.5 (C10), 72.9 (C6), 41.5 (C8), 41.0 (C5).

ESI-MS: (*m/z*) required: $[C_{12}H_{15}NO_2)Na]^+$ = 228.0995, (*m/z*) found: $[C_{12}H_{15}NO_2)Na]^+$ 228.1006.

(*S*)-*N*-Allyl-2-(benzyloxy)-3-phenylpropanamide (1r)

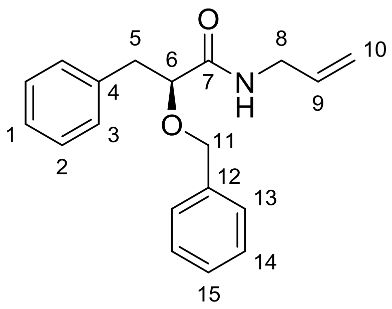

A schlenk flask filled with argon was charged with sodium hydride (56 mg, 1.4 mmol, 1.2 equiv, 60% in mineral oil) and cooled to 0 °C. Dry THF (6 mL) and (*S*)-*N*-allyl-2-hydroxy-3-phenylpropanamide (**S4**) (245 mg, 1.2 mmol, 1.0 equiv) were added. The

reaction mixture was stirred for 1 h before benzyl bromide (0.17 mL, 1.4 mmol, 1.2 equiv) and tetrabutylammonium iodide (37 mg, 0.1 mmol, 0.1 equiv) were added at 0 °C. After slowly warming to ambient temperature the mixture was stirred for 20 h. The reaction mixture was subsequently poured into an aqueous saturated NH_4Cl solution (20 mL) and extracted with dichloromethane (3 × 10 mL). The combined organic phases were dried over $MgSO_4$ and the solvent was removed in vacuo. The residue was purified by column chromatography (cyclohexane : ethyl acetate 4:1). The product (219 mg, 0.74 mmol, 62%) was obtained as a white solid.

R_f: 0.24 (cyclohexane : ethyl acetate, 4:1).

M.p.: 60 °C.

ORD (CH_2Cl_2, c 1.00): $[\alpha]_D^{25}$ = -52.9°.

FT-IR ($\tilde{\nu}$ = cm^{-1}): 3284 (w), 3032 (w), 2917 (w), 2868 (w), 1646 (m), 1524 (m), 1497 (m), 1455 (m), 1432 (m), 1415 (m), 1344 (w), 1334 (w) 1293 (w), 1255 (w), 1205 (w), 1147 (w), 1096 (m), 1083 (m), 1027 (m), 999 (m), 923 (m), 727 (m), 737 (m), 695 (s).

^{1}H NMR (400 MHz, $CDCl_3$, 298 K): δ [ppm] = 7.39–7.31 (m, 4H, H-C^{Ar}), 7.34–7.24 (m, 4H, H-C^{Ar}), 7.24–7.18 (m, 2H, H-C^{Ar}), 6.59 (br-s, 1H, NH), 5.72 (ddt, $^3J_{HH}$ = 16.3, 10.7, 5.5 Hz, 1H, H-C9), 5.15–5.02 (m, 2H, H-C10), 4.53–4.43 (m, 2H, H-C11), 4.13 (dd, $^3J_{HH}$ = 7.7 Hz, 3.6 Hz, 1H, H-C6), 3.95–3.81 (m, 2H, H-C8), 3.22 (dd, $^2J_{HH}$ = 14.0 Hz, $^3J_{HH}$ = 3.6 Hz, 1H, H^a-C5), 2.97 (dd, $^2J_{HH}$ = 14.0 Hz, $^3J_{HH}$ = 7.7 Hz, 1H, H^b-C5).

^{13}C{^{1}H} NMR (101 MHz, $CDCl_3$, 298 K): δ [ppm] = 171.7 (C7), 137.4 (C4), 137.1 (C12), 134.0 (C9), 129.9 (C3), 128.7 (C^{Ar}), 128.4 (C^{Ar}), 128.2 (C^{Ar}), 128.0 (C^{Ar}), 126.7 (C^{Ar}), 116.4 (C10), 81.1 (C6), 73.2 (C11), 41.3 (C8), 39.2 (C5).

ESI-MS: (*m/z*) required: $[C_{19}H_{21}NO_2)Na]^+$ = 318.1465, (*m/z*) found: $[C_{19}H_{21}NO_2)Na]^+$ 318.1466.

***tert*-Butyl (*S*)-(1-hydroxy-3-phenylpropan-2-yl)carbamate (S5)[17]**

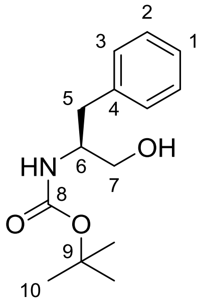

This compound was prepared according to a modified procedure by Sani et al.[16]. *N*-Boc-L-phenylalanine (795 mg, 3.0 mmol, 1.0 equiv) was dissolved in THF (4 mL) and triethylamine (0.52 mL, 3.8 mmol, 1.3 equiv) was added. The mixture was cooled to 0 °C and methyl chloroformate (0.23 mL, 3.0 mmol, 1.0 equiv) was added dropwise via syringe. The reaction mixture was stirred for 30 min at 0 °C before the resulting precipitate was filtered off and washed with THF (5 mL). The filtrate was added dropwise *via* syringe to a solution of sodium borohydride (283 mg, 7.5 mmol, 2.5 equiv) in water (4 mL) at 0 °C. The reaction mixture was stirred for 1.5 h after slowly warming to ambient temperature. The resulting suspension was poured into water (20 mL) and extracted with diethyl ether (1 × 20 mL) and dichloromethane (3 × 20 mL). The combined organic phases were dried over $MgSO_4$ and the solvent was removed in vacuo. The residue was purified by column chromatography (cyclohexane : ethyl acetate, 2:1) and the product (454 mg, 1.8 mmol, 60%) was obtained as a white solid.

R_f: 0.20 (cyclohexane : ethyl acetate, 2:1).

M.p.: 96 °C.

ORD (CH_2Cl_2, c 1.00): $[\alpha]_D^{25}$ = -22.6°.

^{1}H NMR (300 MHz, $CDCl_3$, 298 K): δ [ppm] = 7.38–7.16 (m, 5H, H-C^{Ar}), 4.75 (br-s, 1H, NH), 3.86 (br-s, 1H, H-C6), 3.67 (dd, $^2J_{HH}$ = 11.0 Hz, $^3J_{HH}$ = 3.7 Hz, 1H, H^a-C7), 3.55 (dd, $^2J_{HH}$ = 11.0 Hz, $^3J_{HH}$ = 5.3 Hz, 1H, H^b-C7), 2.84 (d, $^3J_{HH}$ = 7.1 Hz, 2H, H-C5), 2.19 (s, 1H, OH), 1.41 (s, 9H, H-C10).

The analytical data is in agreement with literature values[17].

tert-Butyl (_S_)-(1-oxo-3-phenylpropan-2-yl)carbamate (S6)[18]

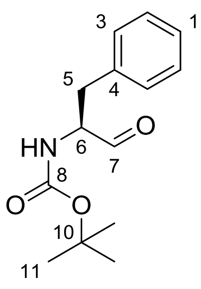

This compound was prepared according to a modified procedure by Sani et al.[16]. *tert*-Butyl (*S*)-(1-hydroxy-3-phenylpropan-2-yl)carbamate (**S5**) (377 mg, 1.5 mmol, 1.0 equiv) was dissolved in dichloromethane (13 mL) and cooled to 0 °C. Sodium bicarbonate (2.52 g, 30.0 mmol, 20 equiv) and freshly prepared Dess–Martin periodinane (700 mg, 1.65 mmol, 1.1 equiv) were added sequentially. The mixture was stirred at 0 °C for 10 min before the ice bath was

removed. The mixture was stirred at ambient temperature for 5 h. Diethyl ether (15 mL) was added and a saturated aqueous solution of sodium sulphate and sodium bicarb (10 mL) was added slowly. The mixture was extracted with dichloromethane (3 × 30 mL). The combined organic phases were dried over $MgSO_4$ and the solvent was removed in vacuo. The residue was purified by column chromatography (cyclohexane : ethyl acetate, 4:1) and the product (304 mg, 1.2 mmol, 81%) was obtained as a white solid.

R_f: 0.33 (cyclohexane : ethyl acetate, 4:1).

M.p.: 85 °C.

ORD (CH_2Cl_2, c 1.00): $[\alpha]_D^{25}$ = 41.6°.

^{1}H NMR (300 MHz, $CDCl_3$, 298 K): δ [ppm] = 9.63 (s, 1H, H-C7), 7.37–7.21 (m, 3H, H-C^{Ar}), 7.21–7.14 (m, 2H, H-C^{Ar}), 5.05 (br-d, $^3J_{HH}$ = 6.9 Hz, 1H, NH), 4.43 (q, $^3J_{HH}$ = 6.8 Hz, 1H, H-C6), 3.12 (d, $^3J_{HH}$ = 6.8 Hz, 2H, H-C5), 1.44 (s, 9H, H-C11).

The analytical data is in agreement with literature values[18].

tert-Butyl (*S*)-(1-phenylbut-3-en-2-yl)carbamate (S7)[19]

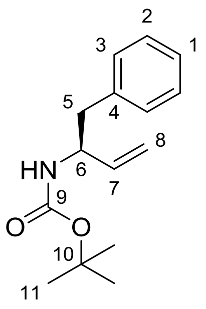

This compound was prepared according to a modified procedure by Luly et al.[19]. An oven dried schlenk flask was charged with triphenylmethylphosphonium bromide (536 mg, 1.5 mmol, 1.5 equiv) and dry toluene (10 mL) under argon. The suspension was cooled to 0 °C and a solution of KHMDS (3 mL, 1.5 mmol, 1.5 equiv, 0.5 M in toluene) was added dropwise *via* syringe. The mixture was stirred for 1.5 h at 0 °C before being cooled to −78 °C. *tert*-Butyl (*S*)-(1-oxo-3-phenylpropan-2-yl)carbamate (**S6**) (249 mg, 1.0 mmol, 1.0 equiv) was added in one portion and the resulting yellow solution was stirred for 10 min at −78 °C before the ice bath was removed. The reaction mixture was stirred at ambient temperature for 1.5 h before a saturated aqueous NH_4Cl solution was added. The biphasic mixture was extracted with ethyl acetate (3 × 20 mL) and the combined organic phases were dried over $MgSO_4$. The solvent was removed in vacuo and the residue was purified by column chromatography (cyclohexane : ethyl acetate, 20:1) and the product (198 mg, 0.8 mmol, 80%) was obtained as a colourless solid.

R$_f$: 0.23 (cyclohexane : ethyl acetate, 20:1).

M.p.: 67 °C.

ORD (CH_2Cl_2, c 1.00): $[\alpha]_D^{25}$ = 30.0°.

^{1}H NMR (300 MHz, $CDCl_3$, 298 K): δ [ppm] = 7.32–7.14 (m, 5H, H-C^{Ar}), 5.79 (ddd, $^3J_{HH}$ = 17.2, 10.4, 5.2 Hz, 1H, H-C7), 5.11 (dt, $^3J_{HH}$ = 17.2 Hz, $^2J_{HH}$ = $^4J_{HH}$ = 1.4 Hz, 1H, H^Z-C8), 5.08 (dt, $^3J_{HH}$ = 10.4 Hz, $^2J_{HH}$ = $^4J_{HH}$ = 1.3 Hz, 1H, H^E-C8), 4.52–4.20 (m, 2H, H-C6, NH), 2.83 (d, $^3J_{HH}$ = 6.5 Hz, 2H, H-C5), 1.40 (s, 9H, H-C11).

The analytical data is in agreement with literature values[19].

(S)-*N*-(1-Phenylbut-3-en-2-yl)benzamide (1s)

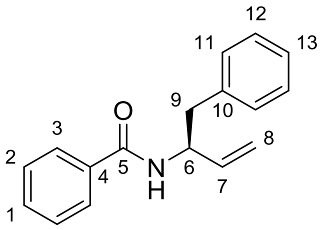

tert-Butyl (S)-(1-phenylbut-3-en-2-yl)carbamate (**S7**) (151 mg, 0.60 mmol, 1.0 equiv) was dissolved in a solution of HCl (1.8 mL, 3 M in ethyl acetate) and the mixture was stirred for 1 h at ambient temperature. The volatiles were removed in vacuo and the residue was dissolved in dichloromethane (1.8 mL). The solution was cooled to 0 °C and triethylamine (0.2 mL, 1.46 mmol, 2.4 equiv) was added via syringe. To the resulting suspension benzoyl chloride (90 µL, 0.79 mmol, 1.3 equiv) was added dropwise and the mixture was stirred for 15 min at 0 °C. The ice bath was removed and the stirring was continued for 1 h before the mixture was poured into an aqueous solution of HCl (2 mL, 1 M). The organic phase was separated and the aqueous phase was extracted with dichloromethane (2 × 5 mL). The combined organic phases were dried over $MgSO_4$ and the solvent was removed in vacuo. The residue was purified by column chromatography (cyclohexane : ethyl acetate, 7:1) and the product (143 mg, 0.57 mmol, 95%) was obtained as a colourless solid.

R$_f$: 0.16 (cyclohexane : ethyl acetate, 7:1).

M.p.: 120 °C.

ORD (CH_2Cl_2, c 1.00): $[\alpha]_D^{25}$ = 31.8°.

FT-IR ($\tilde{\nu}$ = cm^{-1}): 3293 (m), 3062 (w), 3030 (w), 2919 (w), 2855 (w), 1633 (s), 1603 (m), 1579 (m), 1538 (s), 1487 (m), 1446 (m), 1455 (m), 1337 (s), 1291 (m), 1276 (m), 1192 (m), 1154 (w), 1083 (m), 1056 (m), 1026 (m), 1001 (w), 990 (m), 926 (s), 857 (m), 799 (m), 751 (m), 692 (s), 665 (s).

^{1}H NMR (600 MHz, $CDCl_3$, 298 K): δ [ppm] = 7.72–7.67 (m, 2H, H-C3), 7.51–7.46 (m, 1H, H-C1), 7.43–7.39 (m, 2H, H-C2), 7.33–7.29 (m, 2H, H-C12), 7.26–7.22 (m, 3H, H-C11, H-C13), 6.06 (d, $^{3}J_{HH}$ = 8.3 Hz, 1H, NH), 5.92 (ddd, $^{3}J_{HH}$ = 17.2, 10.4, 5.5 Hz, 1H, H-C7), 5.21–5.12 (m, 2H, H-C8), 5.00 (dtdt, $^{3}J_{HH}$ = 8.3, 6.8, 5.5 Hz, $^{4}J_{HH}$ = 1.6 Hz, 1H, H-C6), 3.01 (dd, $^{3}J_{HH}$ = 6.8 Hz, $^{4}J_{HH}$ = 1.5 Hz, 2H, H-C9).

^{13}C{^{1}H} NMR (151 MHz, $CDCl_3$, 298 K): δ [ppm] = 166.9 (C5), 137.6 (C7), 137.2 (C10), 134.8 (C4), 131.6 (C1), 129.7 (C11), 128.7 (C2), 128.6 (C12), 127.0 (C3), 126.8 (C13), 115.6 (C8), 52.5 (C6), 41.1 (C9).

ESI-MS: (*m/z*) requires: $[(C_{17}H_{17}NO)Na]^+$ = 274.1202, (*m/z*) found: $[(C_{17}H_{17}NO)Na]^+$ = 274.1213.

Fluorocyclisations of (homo-)allyl amides

General procedure for the fluorocyclisation of amides

Unless stated otherwise, a Teflon® screw cap vial was charged with amide (0.2 mmol, 1.0 equiv), *p*-iodotoluene (4.4 mg, 0.02 mmol, 10 mol %) and dichloromethane (1 mL). To this solution was added the previously prepared amine:HF, 1:4.5 source (1 mL) via syringe. The mixture was stirred for 2 min. before Selectfluor® (106 mg, 0.3 mmol, 1.5 equiv) was added. The reaction mixture was stirred at ambient temperature for the time indicated before being diluted with dichloromethane (2 mL). To the diluted solution was slowly added a saturated aqueous solution of $NaHCO_3$ (2 mL) under vigorous stirring. The biphasic mixture was subsequently poured into a saturated aqueous solution of $NaHCO_3$ (20 mL) in a separation funnel. The mixture was extracted with dichloromethane (3 × 10 mL) and the combined organic phases were dried over $MgSO_4$. The solvent was removed in vacuo and the NMR yield was determined from the crude material using ethyl fluoroacetate (19.2 µl, 0.2 mmol, 1.0 equiv) as the internal standard. The residue was purified by column chromatography.

HF source

A mixture of amine:HF, 1:4.5 (1 mL) was obtained by mixing 0.32 mL of Pyr·$(HF)_x$ and 0.68 mL of NEt_3·3HF.

A stock solution was prepared and stored at −30 °C in a PE vial.

5-(Fluoromethyl)-2-phenyl-4,5-dihydrooxazole (2a)

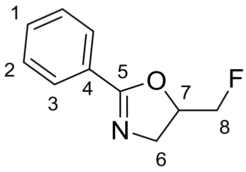

Prepared according to the general procedure from *N*-allylbenzamide (**1a**) (32 mg, 200 µmol) over 26 h. The product was purified by column chromatography (cyclohexane : ethyl acetate, 2:1) and obtained as a colourless oil (24 mg, 134 µmol, 67%).

R*f*: 0.21 (cyclohexane : ethyl acetate, 2:1).

FT-IR ($\tilde{\nu}$ = cm^{-1}): 3111 (w), 2948 (w), 2879 (w), 1722 (w), 1651 (s), 1580 (w), 1496 (w), 1450 (m), 1365 (m), 1337 (m), 1319 (w), 1262 (s), 1177 (w), 1079 (s), 1062 (s), 1025 (s), 1003 (m), 987 (s), 952 (m), 926 (w), 888 (w), 833 (w), 779 (m), 692 (s), 674 (s).

^{1}H NMR (400 MHz, $CDCl_3$, 298 K): δ [ppm] = 8.00–7.93 (m, 2H, H-C3), 7.53–7.45 (m, 1H, H-C1), 7.45–7.38 (m, 2H, H-C2), 5.03–4.83 (m, 1H, H-C7), 4.60 (ddd, $^2J_{HF}$ = 47.2 Hz, $^2J_{HH}$ = 10.4 Hz, $^3J_{HH}$ = 3.1 Hz, 1H, H^a-C8), 4.51 (ddd, $^2J_{HF}$ = 47.1 Hz, $^2J_{HH}$ = 10.4 Hz, $^3J_{HH}$ = 5.5 Hz, 1H, H^b-C8), 4.16 (ddd, $^2J_{HH}$ = 14.9 Hz, $^3J_{HH}$ = 10.2 Hz, $^4J_{HF}$ = 1.5 Hz, 1H, H^a-C6), 3.88 (dd, $^2J_{HH}$ = 14.8 Hz, $^3J_{HH}$ = 7.5 Hz, 1H, H^b-C6).

^{13}C{^{1}H} NMR (101 MHz, $CDCl_3$, 298 K): δ [ppm] = 164.3 (C5), 131.7 (C2), 128.5 (C1), 128.4 (C4), 127.4 (C3), 83.5 (d, $^1J_{CF}$ = 175.3 Hz, C8), 77.8 (d, $^2J_{CF}$ = 19.6 Hz, C7), 55.9 (d, $^3J_{CF}$ = 5.9 Hz, C6).

^{19}F NMR (282 MHz, $CDCl_3$, 298 K): δ [ppm] = -229.0 (tdd, $^2J_{HF}$ = 47.2 Hz, $^3J_{HF}$ = 19.6 Hz, $^4J_{HF}$ = 1.6 Hz, F-C8).

ESI-MS: (*m/z*) requires: $[(C_{10}H_{10}FNO)H]^+$ = 180.0819, (*m/z*) found: $[(C_{10}H_{10}FNO)H]^+$ = 180.0817.

5-(Fluoromethyl)-2-(4-methoxyphenyl)-4,5-dihydrooxazole (2b)

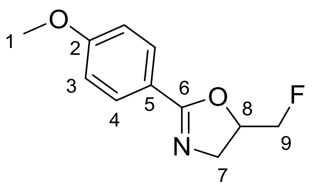

Prepared according to the general procedure from *N*-allyl-4-methoxybenzamide (**1b**) (38 mg, 200 µmol) over 26 h. The product was purified by column chromatography (cyclohexane : ethyl acetate, 2:1) and obtained as a colourless solid (28 mg, 136 µmol, 68%).

R*f*: 0.13 (cyclohexane : ethyl acetate, 2:1).

M.p.: 80 °C.

FT-IR ($\tilde{\nu}$ = cm^{-1}): 3068 (w), 2938 (w), 2876 (w), 2842 (w), 1912 (w), 1716 (w), 1644 (s), 1607 (s), 1515 (s), 1460 (m), 1443 (m), 1419 (w), 1367 (s), 1339 (s), 1301 (m), 1250 (s), 1175 (s), 1119 (m), 1075 (s), 1023 (s), 993 (s), 944 (s), 929 (s), 891 (s), 848 (s), 833 (s), 794 (s), 758 (m), 737 (s), 673 (s).

^{1}H NMR (400 MHz, $CDCl_3$, 298 K) δ [ppm] = 7.90 (m, 2H, H-C4), 6.91 (m, 2H, H-C3), 4.91 (ddddd, $^3J_{HF}$ = 19.0 Hz, $^3J_{HH}$ = 10.4, 7.5, 5.7, 3.1 Hz, 1H, H-C8), 4.58 (ddd, $^2J_{HF}$ = 47.2 Hz, $^2J_{HH}$ = 10.4 Hz, $^3J_{HH}$ =, 3.1 Hz, 1H, H^a-C9), 4.50 (ddd, $^2J_{HF}$ = 47.1 Hz, $^2J_{HH}$ = 10.4 Hz, $^3J_{HH}$ = 5.7 Hz, 1H, H^b-C9), 4.41 (ddd, $^2J_{HH}$ = 14.7 Hz, $^3J_{HH}$ = 10.2 Hz, $^4J_{HF}$ = 1.5 Hz, 1H, H^a-C7), 3.85 (s, 3H, H-C1), 3.83 (dd, $^2J_{HH}$ = 14.7 Hz, $^3J_{HH}$ = 7.4 Hz, 1H, H^b-C7).

^{13}C{^{1}H} NMR (101 MHz, $CDCl_3$, 298 K): δ [ppm] = 164.1 (C6), 162.4 (C2), 130.2 (C4), 119.9 (C5), 113.9 (C3), 83.6 (d, $^1J_{CF}$ = 175.1 Hz, C9), 77.6 (d, $^2J_{CF}$ = 19.6 Hz, C8), 55.8 (d, $^3J_{CF}$ = 5.9 Hz, C7), 55.5 (C1).

^{19}F NMR (282 MHz, $CDCl_3$, 298 K): δ [ppm] = -228.6 (tdd, $^2J_{HF}$ = 47.2 Hz, $^3J_{HF}$ = 19.1 Hz, $^4J_{HF}$ = 1.5 Hz, F-C9).

ESI-MS: (*m/z*) requires: $[(C_{11}H_{12}FNO_2)H]^+$ = 210.0925, (*m/z*) found: $[(C_{11}H_{12}FNO_2)H]^+$ = 210.0923.

5-(Fluoromethyl)-2-(4-nitrophenyl)-4,5-dihydrooxazole (2c)

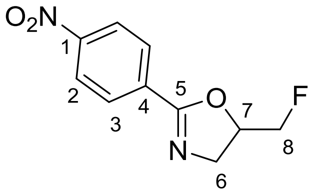

Prepared according to the general procedure from *N*-allyl-4-nitrobenzamide (**1c**) (206 mg, 1.0 mmol, 1.0 equiv) with

p-Iodotoluene (22 mg, 0.1 mmol, 10 mol %) and Selectfluor® (530 mg, 1.5 mmol, 1.5 equiv) in dichloromethane (5 mL) and amine:HF, 1:4.5 (5 mL) over 25 h. The product was purified by column chromatography (cyclohexane : ethyl acetate, 2:1) and obtained as a colourless solid (133 mg, 0.59 mmol, 59%). Single crystals suitable for X-ray analysis could be obtained by slow evaporation of a dichloromethane / *n*-heptane solution.

R_f: 0.18 (cyclohexane : ethyl acetate, 2:1).

M.p.: 115 °C.

FT-IR ($\tilde{\nu}$ = cm^{-1}): 3112 (w), 3085 (w), 2963 (w), 2925 (w), 1717 (w), 1647 (m), 1597 (m), 1516 (s), 1494 (w), 1454 (w), 1412 (m), 1400 (s), 1316 (m), 1263 (m), 1193 (w), 1108 (m), 1085 (s), 1064 (m), 1042 (s), 1005 (s), 986 (s), 916 (w), 897 (m), 869 (s), 854 (s), 768 (m), 737 (w), 713 (m), 699 (s), 661 (s).

^{1}H NMR (400 MHz, $CDCl_3$, 298 K): δ [ppm] = 8.27 (m, 2H, H-C3), 8.13 (m, 2H, H-C2), 4.99 (ddddd, $^3J_{HF}$ = 20.8 Hz, $^3J_{HH}$ = 10.4, 7.8, 5.1, 2.8 Hz, 1H, H-C7), 4.65 (ddd, $^2J_{HF}$ = 47.4 Hz, $^3J_{HH}$ = 10.6, 2.8 Hz, 1H, H^a-C8), 4.52 (ddd, $^2J_{HF}$ = 46.9 Hz, $^2J_{HH}$ = 10.6 Hz $^3J_{HH}$ = 5.1 Hz, 1H, H^b-C8), 4.21 (ddd, $^2J_{HH}$ = 15.4 Hz, $^3J_{HH}$ = 10.3 Hz, $^4J_{HF}$ = 1.5 Hz, 1H, H-C6), 3.95 (dd, $^2J_{HH}$ = 15.3 Hz, $^3J_{HH}$ = 7.7 Hz,1H, H-C6).

^{13}C{^{1}H} NMR (101 MHz, $CDCl_3$, 298 K): δ [ppm] = 162.4 (C5), 149.75 (C1), 133.2 (C4), 129.4 (C2), 123.7 (C3), 83.3 (d, $^1J_{CF}$ = 175.9 Hz, C7), 78.5 (d, $^2J_{CF}$ = 19.5 Hz, C8), 56.1 (d, $^3J_{CF}$ = 6.1 Hz, C6).

^{19}F NMR (282 MHz, $CDCl_3$, 298 K): δ [ppm] = -230.4 (tdd, $^2J_{HF}$ = 47.1 Hz, $^3J_{HF}$ = 20.9 Hz, $^4J_{HF}$ = 1.5 Hz, F-C8).

ESI-MS: (*m/z*) requires: $[(C_{10}H_9FN_2O_3)H]^+$ = 225.0670, (*m/z*) found: $[(C_{10}H_9FN_2O_3)H]^+$ = 225.0675.

5-(Fluoromethyl)-2-(4-(trifluoromethyl)phenyl)-4,5-dihydrooxazole (2d)

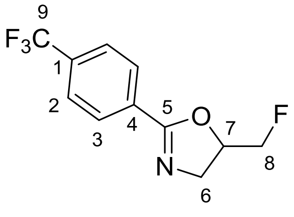

Prepared according to the general procedure from *N*-allyl-4-(trifluoromethyl)benzamide (**1d**) (46 mg, 200 µmol) over 23 h.

The product was purified by column chromatography (cyclohexane : ethyl acetate, 2:1) and obtained as a colourless oil (34 mg, 138 µmol, 69%).

R$_f$: 0.17 (cyclohexane : ethyl acetate, 2:1).

FT-IR ($\tilde{\nu}$ = cm^{-1}): 2951 (w), 2887 (w), 1816 (w), 1651 (m), 1620 (w), 1582 (w), 1515 (w), 1461 (w), 1413 (m), 1337 (w), 1322 (s), 1260 (m), 1242 (w), 1185 (w), 1154 (m), 1116 (s), 1104 (s), 1075 (s), 1062 (s), 1017 (s), 991 (m), 924 (s), 928 (m), 889 (m), 851 (s), 834 (s), 753 (m), 727 (w), 695 (m), 670 (s), 635 (w), 609 (m).

^{1}H NMR (600 MHz, $CDCl_3$, 298 K): δ [ppm] = 8.07 (m, 2H, H-C3), 7.67 (m, 2H, H-C2), 4.95 (ddddd, $^3J_{HF}$ = 20.3 Hz, $^3J_{HH}$ = 10.4, 7.6, 5.4, 2.9 Hz, 1H, H-C7), 4.62 (ddd, $^2J_{HF}$ = 47.3 Hz, $^2J_{HH}$ = 10.6 Hz, $^3J_{HH}$ = 2.9 Hz, 1H, H^a-C8), 4.51 (ddd, $^2J_{HF}$ = 47.3 Hz, $^2J_{HH}$ = 10.6 Hz, $^3J_{HH}$ = 5.3 Hz, 1H, H^b-C8), 4.18 (ddd, $^2J_{HH}$ = 15.0 Hz, $^3J_{HH}$ = 10.4 Hz, $^4J_{HH}$ = 1.5 Hz, 1H, H^a-C6), 3.91 (dd, $^2J_{HH}$ = 15.0 Hz, $^3J_{HH}$ = 7.6 Hz, 1H, H^b-C6).

^{13}C{^{1}H} NMR (151 MHz, $CDCl_3$, 298 K): δ [ppm] = 163.0 (C5), 133.3 (q, $^2J_{CF}$ = 32.6 Hz, C1), 130.8 (C4), 128.8 (C3), 125.5 (q, $^3J_{CF}$ = 3.8 Hz, C2), 123.9 (q, $^1J_{CF}$ = 272.5 Hz, C9), 83.4 (d, $^1J_{CF}$ = 175.6 Hz, C8), 78.1 (d, $^2J_{CF}$ = 19.5 Hz, C7), 56.0 (d, $^3J_{CF}$ = 6.0 Hz, C6).

^{19}F{^{1}H} NMR (564 MHz, $CDCl_3$, 298 K): δ [ppm] = -63.0 (s, 3F, CF_3), -229.9 (s, 1F, F-C8).

^{19}F NMR (564 MHz, $CDCl_3$, 298 K): δ [ppm] = -63.0 (s, 3F, CF_3), -229.9 (dt, $^2J_{FH}$ = 47.3 Hz, $^3J_{FH}$ = 20.3 Hz, 1F, F-C8).

ESI-MS: (*m/z*) requires: $[(C_{11}H_9NOF_4)H]^+$ = 248.0693, (*m/z*) found: $[(C_{11}H_9NOF_4)H]^+$ = 248.0691.

4-(5-(Fluoromethyl)-4,5-dihydrooxazol-2-yl)benzaldehyde (2e)

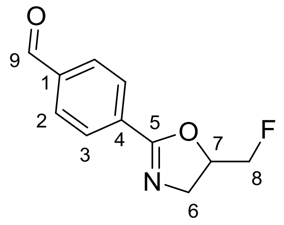

Prepared according to the general procedure from *N*-allyl-4-formylbenzamide (**1e**) (38 mg, 200 µmol) over 24 h. The product was purified by column chromatography (cyclohexane :

ethyl acetate, 1:1) and obtained as a colourless solid (13 mg, 63 µmol, 31%).

R$_f$: 0.14 (cyclohexane : ethyl acetate, 1:1).

M.p.: 65–67 °C.

FT-IR ($\tilde{\nu}$ = cm^{-1}): 2961 (w), 2848 (w), 2738 (w), 1697 (s), 1648 (s), 1573 (m), 1508 (w), 1455 (w), 1421 (m), 1389 (m), 1369 (m), 1335 (m), 1300 (m), 1263 (s), 1202 (s), 1173 (m), 1068 (s), 1016 (s), 944 (s), 923 (m), 883 (m), 830 (s), 731 (s), 687 (s), 663 (s), 630 (m), 604 (s).

^{1}H NMR (600 MHz, $CDCl_3$, 298 K): δ [ppm] = 10.07 (s, 1H, H-C9), 8.15–8.10 (m, 2H, H-C3), 7.96–7.91 (m, 2H, H-C2), 4.97 (ddddd, $^3J_{HF}$ = 20.4 Hz, $^3J_{HH}$ = 10.4, 7.7, 5.3, 2.9 Hz, 1H, H-C7), 4.63 (ddd, $^2J_{HF}$ = 47.2 Hz, $^2J_{HH}$ = 10.5 Hz, $^3J_{HH}$ = 2.9 Hz, 1H, H^a-C8), 4.53 (ddd, $^2J_{HF}$ = 47.2 Hz, $^2J_{HH}$ = 10.5 Hz, $^3J_{HH}$ = 5.3 Hz, 1H, H^b-C8), 4.20 (ddd, $^2J_{HH}$ = 15.3 Hz, $^3J_{HH}$ = 10.4 Hz, $^4J_{HF}$ = 1.5 Hz, 1H, H^a-C6), 3.94 (dd, $^2J_{HH}$ = 15.3 Hz, $^3J_{HH}$ = 7.7 Hz, 1H, H^b-C6).

^{13}C{^{1}H} NMR (151 MHz, $CDCl_3$, 298 K): δ [ppm] = 191.8 (C9), 163.3 (C5), 138.3 (C1), 132.7 (C4), 129.7 (C2), 129.0 (C3), 83.3 (d, $^1J_{CF}$ = 175.8 Hz, C8), 78.2 (d, $^2J_{CF}$ = 19.6 Hz, C7), 56.0 ($^3J_{CF}$ = 5.9 Hz, C6).

^{19}F NMR (564 MHz, $CDCl_3$, 298 K): δ [ppm] = -229.9 (tdd, $^2J_{FH}$ = 47.2 Hz, $^3J_{FH}$ = 20.4 Hz, $^4J_{FH}$ = 1.5 Hz, F-C8).

ESI-MS: (*m/z*) requires: $[(C_{11}H_{10}NO_2F)H]^+$ = 208.0768, (*m/z*) found: $[(C_{11}H_{10}NO_2F)H]^+$ = 208.0773.

2-(2-Bromophenyl)-5-(fluoromethyl)-4,5-dihydrooxazole (2f)

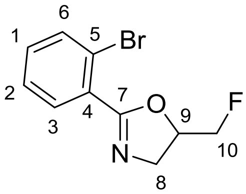

Prepared according to the general procedure from *N*-allyl-2-bromobenzamide (**1f**) (48 mg, 200 µmol) over 40 h. The product was purified by column chromatography (cyclohexane : ethyl acetate, 2:1) and obtained as a colourless oil (31 mg, 120 µmol, 60%).

R_f: 0.14 (cyclohexane : ethyl acetate, 1:1).

FT-IR ($\tilde{\nu}$ = cm^{-1}): 2949 (w), 2879 (w), 1734 (w), 1652 (m), 1590 (w), 1565 (w), 1474 (w), 1455 (w), 1431 (m), 1360 (w), 1333 (m), 1315 (w), 1270 (m), 1242 (m), 1194 (w), 1081 (s), 1027 (s), 1004 (s), 982 (m), 951 (m), 925 (w), 888 (m), 848 (m), 831 (m), 761 (s), 727 (s), 706 (m), 672 (m), 643 (m).

^{1}H NMR (600 MHz, $CDCl_3$, 298 K): δ [ppm] = 7.72 (ddd, $^3J_{HH}$ = 7.7 Hz, $^4J_{HH}$ = 1.8 Hz, $^5J_{HH}$ = 0.4 Hz, 1H, H-C3), 7.65 (ddd, $^3J_{HH}$ = 7.9 Hz, $^4J_{HH}$ = 1.3 Hz, $^5J_{HH}$ = 0.4 Hz, 1H, H-C6), 7.35 (td, $^3J_{HH}$ = 7.6 Hz, $^4J_{HH}$ = 1.4 Hz, 1H, H-C2), 7.29 (ddd, $^3J_{HH}$ = 7.9, 7.4 Hz, $^4J_{HH}$ = 1.8 Hz, 1H, H-C1), 4.93 (ddddd, $^3J_{HF}$ = 19.4 Hz, $^3J_{HH}$ = 10.3, 7.4, 5.4, 3.2 Hz, 1H, H-C9), 4.60 (ddd, $^2J_{HF}$ = 47.1 Hz, $^2J_{HH}$ = 10.4 Hz, $^3J_{HH}$ = 3.2 Hz, 1H, H^a-C10), 4.53 (ddd, $^2J_{HF}$ = 47.1 Hz, $^2J_{HH}$ = 10.4 Hz, $^3J_{HH}$ = 5.4 Hz, 1H, H^b-C10), 4.19 (ddd, $^2J_{HH}$ = 14.9 Hz, $^3J_{HH}$ = 10.3 Hz, $^4J_{HF}$ = 1.5 Hz, 1H, H^a-C8), 3.93 (dd, $^2J_{HH}$ = 14.9 Hz, $^3J_{HH}$ = 7.4 Hz, 1H, H^b-C8).

^{13}C{^{1}H} NMR (151 MHz, $CDCl_3$, 298 K): δ [ppm] = 163.3 (C7), 134.1 (C6), 131.9 (C1), 131.5 (C3), 129.3 (C4), 127.3 (C2), 122.0 (C5), 83.3 (d, $^1J_{CF}$ = 175.4 Hz, C10), 77.7 (d, $^2J_{CF}$ = 19.8 Hz, C9), 56.3 (d, $^3J_{CF}$ = 5.8 Hz, C8).

^{19}F NMR (564 MHz, $CDCl_3$, 298 K): δ [ppm] = -229.5 (tdd, $^2J_{FH}$ = 47.1 Hz, $^3J_{FH}$ = 19.4 Hz, $^4J_{FH}$ = 1.5 Hz, F-C10).

ESI-MS: (*m/z*) requires: $[(C_{10}H_9NOBrF)H]^+$ = 257.9924, (*m/z*) found: $[(C_{10}H_9NOBrF)H]^+$ = 257.9931.

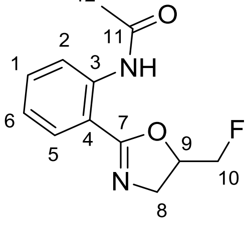

***N*-(2-(5-(Fluoromethyl)-4,5-dihydrooxazol-2-yl)phenyl)acetamide (2g)**

Prepared according to the general procedure from 2-acetamido-*N*-allylbenzamide (**1g**) (44 mg, 200 µmol) over 28 h. The product was purified by column chromatography (cyclohexane : ethyl acetate, 1:1) and obtained as a colourless solid (24 mg, 108 µmol, 52%).

R_f: 0.17 (cyclohexane : ethyl acetate, 1:1).

M.p.:160 °C.

FT-IR ($\tilde{\nu}$ = cm^{-1}): 3177 (w), 2924 (w), 1677 (s), 1606 (m), 1590 (s), 1568 (s), 1476 (s), 1443 (m), 1401 (s), 1378 (m), 1363 (m), 1335 (m), 1319 (m), 1283 (m), 1246 (m), 1224 (m), 1179 (m), 1149 (w), 1123 (s), 1061 (s), 1031 (s), 929 (s), 891 (m), 878 (m), 838 (m), 775 (s), 741 (m), 693 (s).

^{1}H NMR (600 MHz, $CDCl_3$, 298 K): δ [ppm] = 8.05 (dd, $^3J_{HH}$ = 8.0, $^4J_{HH}$ = 1.7 Hz, 1H, H-C5), 7,67 (ddd, $^3J_{HH}$ = 8.5, 7.1 Hz, $^4J_{HH}$ = 1.6 Hz, 1H, H-C1), 7.50 (dd, $^3J_{HH}$ = 8.3 Hz, $^4J_{HH}$ = 1.0 Hz, 1H, H-C2), 7.36 (ddd, $^3J_{HH}$ = 7.9, 7.2 Hz, $^4J_{HH}$ = 1.1 Hz, 1H, H-C6), 4.55 (ddd, $^2J_{HF}$ = 47.1 Hz, $^2J_{HH}$ = 9.7 Hz, $^3J_{HH}$ = 4.7 Hz, 1H, H^a-C10), 4.52 (ddd, $^2J_{HF}$ = 47.1 Hz, $^2J_{HH}$ = 9.7 Hz, $^3J_{HH}$ = 4.8 Hz, 1H, H^b-C10), 4.35 (ddtd, $^3J_{HF}$ = 18.3 Hz, $^3J_{HH}$ = 8.8, 4.8, 3.0 Hz, 1H, H-C9), 4.32 (dd, $^2J_{HH}$ = 14.5 Hz, $^3J_{HH}$ = 3.0 Hz, 1H, H^a-C8), 4.18 (br-s, 1H, NH), 4.14 (dd, $^2J_{HH}$ = 14.5 Hz, $^3J_{HH}$ = 8.8 Hz, 1H, H^b-C8), 2.68 (s, 3H, H-C12).

^{13}C{^{1}H} NMR (151 MHz, $CDCl_3$, 298 K): δ [ppm] = 163.2 (C7), 155.1 (C11), 147.1 (C3), 134.7 (C1), 126.8 (C6), 126.7 (C5), 126.5 (C2), 120.0 (C4), 84.7 (d, $^1J_{CF}$ = 170.8 Hz, C10), 69.1 (d, $^2J_{CF}$ = 20.2 Hz, C9), 47.5 (d, $^3J_{CF}$ = 7.1 Hz, C8), 23.7 (C12).

^{19}F NMR (564 MHz, $CDCl_3$, 298 K): δ [ppm] = -230.9 (td, $^2J_{FH}$ = 47.1 Hz, $^3J_{FH}$ = 18.3 Hz, F-C10).

ESI-MS: (*m/z*) requires: $[(C_{12}H_{13}FN_2O_2)H]^+$ = 237.1034, (*m/z*) found: $[(C_{12}H_{13}FN_2O_2)H]^+$ = 237.1039.

2-(5-Bromo-2-chlorophenyl)-5-(fluoromethyl)-4,5-dihydrooxazole (2h)

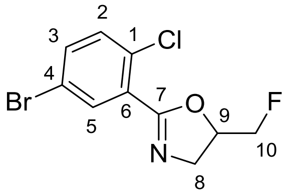

Prepared according to the general procedure from *N*-allyl-5-bromo-2-chlorobenzamide (**1h**) (55 mg, 200 µmol) over 24 h. The product was purified by column chromatography (cyclohexane : ethyl acetate, 5:1) and obtained as a yellow oil (40 mg, 137 µmol, 69%).

R_f: 0.2 (cyclohexane : ethyl acetate, 5:1).

FT-IR ($\tilde{\nu}$ = cm^{-1}): 2948 (w), 2879 (w), 1652 (m), 1584 (w), 1553 (w), 1470 (m), 1403 (w), 1355 (w), 1325 (m), 1312 (w), 1258 (m), 1235 (m), 1193 (w), 1144 (w), 1108 (m), 1088 (m), 1030 (m), 1007 (m), 986 (m), 952 (m), 928 (w), 894 (m), 850 (w), 816 (m), 769 (w), 733 (m), 663 (w).

^{1}H NMR (600 MHz, $CDCl_3$, 298 K): δ [ppm] = 7.94 (d, $^4J_{HH}$ = 2.5 Hz, 1H, H-C2), 7.49 (dd, $^3J_{HH}$ = 8.6 Hz, $^4J_{HH}$ = 2.5 Hz, 1H, H-C3), 7.32 (d, $^3J_{HH}$ = 8.6 Hz, 1H, H-C5), 4.92 (ddddd, $^3J_{HF}$ = 20.4 Hz, $^3J_{HH}$ = 10.4, 7.6, 5.2, 3.0 Hz, 1H, H-C9), 4.60 (ddd, $^2J_{HF}$ = 47.4 Hz, $^2J_{HH}$ = 10.4 Hz, $^3J_{HH}$ = 3.1 Hz, 1H, H^a-C10), 4.47 (ddd, $^2J_{HF}$ = 46.9 Hz, $^2J_{HH}$ = 10.6 Hz, $^3J_{HH}$ = 5.2 Hz, 1H, H^b-C10), 4.21 (ddd, $^2J_{HH}$ = 15.1 Hz, $^3J_{HH}$ 10.3 Hz, $^4J_{HF}$ = 1.5 Hz, 1H, H^a-C8), 3.96 (dd, $^2J_{HH}$ = 15.1 Hz, $^3J_{HH}$ = 7.5 Hz, 1H, H^b-C8).

^{13}C{^{1}H} NMR (151 MHz, $CDCl_3$, 298 K): δ [ppm] = 161.3 (C7), 134.8 (C3), 134.2 (C5), 132.7 (C1), 132.3 (C2), 128.6 (C6), 120.3 (C4), 83.2 (d, $^2J_{CF}$ = 178.8 Hz, C10), 77.8 (d, $^3J_{CF}$ = 19.7 Hz, C9), 56.4 (d, $^4J_{CF}$ = 5.9 Hz, C8).

^{19}F NMR (564 MHz, $CDCl_3$, 298 K): δ [ppm] = -230.1 (tdd, $^2J_{HF}$ = 47.1 Hz, $^3J_{HF}$ = 20.4, $^4J_{HF}$ =1.5 Hz, F-C10).

ESI-MS: (*m/z*) required: $[(C_{10}H_9BrClFNO)H]^+$ = 291.9535, (*m/z*) found: $[(C_{10}H_9BrClFNO)H]^+$ = 291.9554.

2-(5-(Fluoromethyl)-4,5-dihydrooxazol-2-yl)-4-nitrophenol (2i)

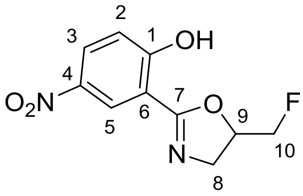

Prepared according to the general procedure from *N*-allyl-5-amino-2-hydroxybenzamide (**1i**) (44 mg, 200 µmol) over 24 h. The product was purified by column chromatography (cyclohexane : ethyl acetate, 1:2, 5% AcOH) and obtained as an orange solid (31 mg, 129 µmol, 65%).

R$_f$: 0.3 (cyclohexane : ethyl acetate, 1:2).

M.p.: 132 °C.

FT-IR ($\tilde{\nu}$ = cm^{-1}): 3094 (w), 2963 (w), 2918 (w), 2853 (w), 1939 (w), 1841 (w), 1650 (m), 1617 (m), 1579 (m), 1518 (m), 1479 (m), 1463 (m), 1428 (m), 1382 (m), 1342 (s), 1306 (s), 1290 (s), 1265 (s), 1227 (m), 1206 (m), 1190 (m), 1121 (s), 1095 (m),

1062 (m), 1009 (m), 941 (s), 922 (m), 909 (m), 860 (m), 832 (s), 815 (s), 758 (m), 744 (m), 706 (m), 678 (m), 639 (m), 614 (m), 567 (m).

^{1}H NMR (600 MHz, $CDCl_3$, 298 K): δ [ppm] = 8.61 (d, $^4J_{HH}$ = 2.7 Hz, 1H, H-C5), 8.26 (dd, $^3J_{HH}$ = 9.2 Hz, $^4J_{HH}$ = 2.8 Hz, 1H, H-C3), 7.07 (d, $^3J_{HH}$ = 9.1 Hz, 1H, H-C2), 5.00 (ddddd, $^3J_{HF}$ = 22.2 Hz, $^3J_{HH}$ = 10.2, 7.3, 4.3, 2.6 Hz, 1H, H-C9), 4.73 (ddd, $^2J_{HF}$ = 47.6 Hz, $^2J_{HH}$ = 10.8 Hz, $^3J_{HH}$ = 2.6 Hz, 1H, H^a-C10), 4.55 (ddd, $^2J_{HF}$ = 46.5 Hz, $^2J_{HH}$ = 10.8 Hz, $^3J_{HH}$ = 4.3 Hz, 1H, H^b-C10), 4.26 (ddd, $^2J_{HH}$ = 14.7 Hz, $^3J_{HH}$ = 10.3 Hz, $^4J_{HF}$ = 1.4 Hz, 1H, H^a-C8), 4.05 (dd, $^2J_{HH}$ = 14.6 Hz, $^3J_{HH}$ = 7.5 Hz, 1H, H^b-C8).

^{13}C{^{1}H} NMR (151 MHz, $CDCl_3$, 298 K): δ [ppm] = 165.1 (C1), 164.6 (C7), 139.8 (C4), 128.9 (C3), 124.9 (C5), 117.8 (C2), 110.3 (C6), 82.6 (d, $^1J_{CF}$ = 176.6 Hz, C10), 77.9 (d, $^2J_{CF}$ = 19.8 Hz, C9), 54.3 (d, $^3J_{CF}$ = 6.4 Hz, C8).

^{19}F NMR (564 MHz, $CDCl_3$, 298 K): δ [ppm] = -232.7 (dddd, $^2J_{HF}$ = 47.7 Hz, 46.4 Hz, $^3J_{HF}$ = 22.5, $^4J_{HF}$ = 1.5 Hz, F-C10).

ESI-MS: (*m/z*) required: $[C_{10}H_9FN_2O_4)Na]^+$ = 263.0439, (*m/z*) found: $[C_{17}H_{12}FNO_2)Na]^+$ 263.0436.

5-(Fluoromethyl)-2-(perfluorophenyl)-4,5-dihydrooxazole (2j)

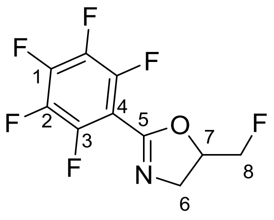

Prepared according to the general procedure from *N*-allyl-2,3,4,5,6-pentafluorobenzamide (**1j**) (47 mg, 188 µmol) with *p*-iodotoluene (4.1 mg, 19 µmol, 10 mol %) and Selectfluor® (100 mg, 282 µmol, 1.5 equiv) in dichloromethane (1 mL) and amine:HF, 1:4.5 (1 mL) over 43 h. The product was purified by column chromatography (cyclohexane : ethyl acetate, 4:1) and obtained as a colourless oil (24 mg, 91 µmol, 48%).

R_f: 0.16 (cyclohexane : ethyl acetate, 4:1).

FT-IR ($\tilde{\nu}$ = cm^{-1}): 2955 (w), 1652 (w), 1670 (w), 1524 (s), 1496 (s), 1459 (w), 1438 (w), 1363 (w), 1345 (m), 1330 (w), 1294 (w), 1256 (w), 1212 (s), 1083 (m), 1048 (w), 980 (s), 955 (m), 940 (m), 906 (m), 851 (m), 831 (w), 802 (s), 744 (w), 681 (w), 619 (w).

^{1}H NMR (400 MHz, $CDCl_3$, 298 K): δ [ppm] = 4.96 (ddddd, $^{3}J_{HF}$ = 21.0 Hz, $^{3}J_{HH}$ = 10.3, 7.6, 4.8, 2.8 Hz, 1H, H-C7), 4.63 (ddd, $^{2}J_{HF}$ = 47.4 Hz, $^{2}J_{HH}$ = 10.6 Hz, $^{3}J_{HH}$ = 2.8 Hz, 1H, H^{a}-C8), 4.50 (ddd, $^{2}J_{HF}$ = 47.4 Hz, $^{2}J_{HH}$ = 10.6 Hz, $^{3}J_{HH}$ = 4.8 Hz, 1H, H^{b}-C8), 4.22 (ddd, $^{2}J_{HH}$ = 15.2 Hz, $^{3}J_{HH}$ = 10.4 Hz, $^{4}J_{HF}$ = 1.4 Hz, 1H, H^{a}-C6), 3.99 (dd, $^{2}J_{HH}$ = 15.2 Hz, $^{3}J_{HH}$ = 7.5 Hz, 1H, H^{b}-C6).

^{13}C{^{1}H} NMR (101 MHz, $CDCl_3$, 298 K): δ [ppm] = 154.7 (C5), 145.9 (m, C^{Ar}), 140.5 (m, C^{Ar}), 137.9 (m, C^{Ar}), 104.6 (C4), 83.0 (d, $^{1}J_{CF}$ = 176.1 Hz, C8), 78.2 (d, $^{2}J_{CF}$ = 19.7 Hz, C7), 56.1 (d, $^{3}J_{CF}$ = 6.0 Hz, C6).

^{19}F NMR (282 MHz, $CDCl_3$, 298 K): δ [ppm] = -136.6 – -136.8 (m, 2F, F-C3), -149.4 (tt, $^{3}J_{FF}$ = 21.0 Hz, $^{4}J_{FF}$ = 4.1 Hz, 1F, F-C1), -160.6 – -160.9 (m, 2F, F-C2), -231.7 (tdd, $^{2}J_{FH}$ = 47.4 Hz, $^{3}J_{FH}$ = 21.0 Hz, $^{4}J_{FH}$ = 1.4 Hz, 1F, F-C8).

ESI-MS: (*m/z*) requires: $[(C_{10}H_6F_6NO)H]^+$ = 270.0348, (*m/z*) found: $[(C_{10}H_6F_6NO)H]^+$ = 270.0352.

6-(Fluoromethyl)-2-phenyl-5,6-dihydro-4H-1,3-oxazine (2k)

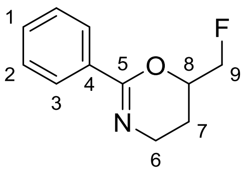

Prepared according to the general procedure from *N*-(but-3-en-1-yl)benzamide (**1k**) (35 mg, 200 µmol) over 26 h. The product was purified by column chromatography (cyclohexane : ethyl acetate, 1:2) and obtained as a colourless solid (16 mg, 84 µmol, 42%).

R_f: 0.19 (cyclohexane : ethyl acetate, 2:1).

M.p.: 45 °C.

FT-IR ($\tilde{\nu}$ = cm^{-1}): 3065 (w), 2986 (w), 2941 (w), 2867 (w), 1654 (s), 1578 (w), 1494 (w), 1450 (m), 1413 (w), 1363 (m), 1354 (m), 1314 (w), 1279 (s), 1256 (m), 1234 (w), 1203 (w), 1145 (s), 1106 (s), 1082 (m), 1066 (m), 1039 (s), 1023 (m), 991 (s), 918 (w), 850 (s), 816 (m), 785 (s), 698 (s), 683 (s), 669 (s).

^{1}H NMR (400 MHz, $CDCl_3$, 298 K): δ [ppm] = 7.97–7.90 (m, 2H, H-C3), 7.48–7.34 (m, 3H, H-C2, H-C1), 4.60 (ddt, $^{2}J_{HF}$ = 47.0 Hz, $^{3}J_{HH}$ =10.1, 4.5 Hz, 2H, H-C9), 4.58–4.44 (m, 1H, H-C8), 3.74 (ddd, $^{2}J_{HH}$ = 16.7 Hz, $^{3}J_{HH}$ = 5.5 Hz, $^{4}J_{HH}$ = 2.8 Hz,1H, H^{a}-C6), 3.63 (ddd, $^{2}J_{HH}$ = 16.5 Hz, $^{3}J_{HH}$ = 10.6, 5.2 Hz, $^{4}J_{HH}$ = 1.5 Hz, 1H, H^{b}-C6), 1.98 (ddt,

$^{2}J_{HH}$ = 13.5 Hz, $^{3}J_{HH}$ = 5.2, 3.0 Hz, 1H, H^{a}-C7), 1.87 (dtd, $^{2}J_{HH}$ = 13.5 Hz, $^{3}J_{HH}$ = 10.4, 5.4 Hz, 1H, H^{b}-C7).

^{13}C{^{1}H} NMR (101 MHz, $CDCl_3$, 298 K): δ [ppm] = 155.5 (C5), 133.5 (C4), 130.7 (C1), 128.2 (C2), 127.1 (C3), 84.4 (d, $^{1}J_{CF}$ = 174.3 Hz, C9), 73.3 (d, $^{2}J_{CF}$ = 20.5 Hz, C8), 42.4 (C6), 22.5 (d, $^{3}J_{CF}$ = 5.7 Hz, C7).

^{19}F NMR (282 MHz, $CDCl_3$, 298 K): δ [ppm] = -230.6 (td, $^{2}J_{HF}$ = 47.0 Hz, $^{3}J_{HF}$ = 19.8 Hz, F-C9).

ESI-MS: (*m/z*) requires: $[(C_{11}H_{12}FNO)H]^+$ = 194.0976, (*m/z*) found: $[(C_{11}H_{12}FNO)H]^+$ = 194.0987.

4-(5-(Fluoromethyl)-4,5-dihydrooxazol-2-yl)-9*H*-fluoren-9-one (2m)

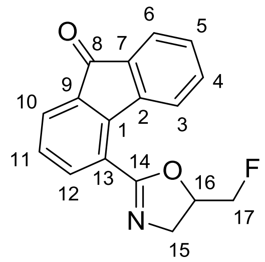

Prepared according to the general procedure from *N*-allyl-9-oxo-9*H*-fluorene-4-carboxamide (**1m**) (53 mg, 200 µmol) over 24 h. The product was purified by column chromatography (cyclohexane : ethyl acetate, 1:2) and obtained as a yellow oil (27 mg, 96 µmol, 48%).

R*f*: 0.2 (cyclohexane : ethyl acetate, 1:2).

FT-IR ($\tilde{\nu}$ = cm^{-1}): 3057 (w), 2950 (w), 2879 (w), 1713 (m), 1669 (m), 1606 (m), 1588 (m), 1468 (w), 1453 (m), 1371 (w), 1287 (m), 1263 (m), 1189 (m), 1090 (m), 1035 (m), 982 (m), 951 (w), 914 (m), 894 (m), 851 (m), 754 (s), 670 (m), 707 (m), 656 (m).

^{1}H NMR (600 MHz, $CDCl_3$, 298 K): δ [ppm] = 7.64–7.60 (m, 2H, H-C6, H-C10), 7.54–7.47 (m, 4H, H-C3, H-C4, H-C11, H-C12), 7.31 (td, $^{3}J_{HH}$ = 7.3 Hz, 1.1 Hz, 1H, H-C5), 5.05 (ddddd, $^{3}J_{HF}$ = 17.6 Hz, $^{3}J_{HH}$ = 13.4, 9.6, 5.9, 3.6 Hz, 1H, H-C16), 4.73 (ddd, $^{2}J_{HF}$ = 47.0 Hz, $^{2}J_{HH}$ = 10.3 Hz, $^{3}J_{HH}$ = 5.9 Hz, 1H; H^{a}-C17), 4.65 (ddd, $^{2}J_{HF}$ = 47.0 Hz, $^{2}J_{HH}$ = 10.3 Hz, $^{3}J_{HH}$ = 3.6 Hz, 1H, H^{b}-C17), 4.22 (ddd, $^{2}J_{HH}$ = 14.7 Hz, $^{3}J_{HH}$ = 10.2 Hz, $^{4}J_{HF}$ = 1.4 Hz, 1H, H^{a}-C15), 3.94 (dd, $^{2}J_{HH}$ = 14.8 Hz, $^{3}J_{HH}$ = 7.3 Hz, 1H, H^{b}-C15).

^{13}C{^{1}H} NMR (151 MHz, $CDCl_3$, 298 K): δ [ppm] = 191.6 (C8), 163.1 (C14), 145.2 (C13), 143.4 (C2), 134.6 (C12), 134.4 (C11), 134.0 (C7), 132.2 (C1), 130.1 (C4),

129.7 (C5), 126.9 (C9), 124.6 (C6), 122.3 (C10), 120.4 (C3), 83.4 (d, $^{1}J_{CF}$ = 174.3 Hz, C17), 78.4 (d, $^{2}J_{CF}$ = 20.0 Hz, C16), 56.4 (d, $^{3}J_{CF}$ = 5.8 Hz, C15).

^{19}F NMR (564 MHz, $CDCl_3$, 298 K): δ [ppm] = -228.3 (tdd, $^{2}J_{HF}$ = 47.0 Hz, $^{3}J_{HF}$ = 17.8, $^{4}J_{HF}$ = 1.3 Hz, F-C17).

ESI-MS: (*m/z*) required: $[C_{17}H_{12}FNO_2)Na]^+$ = 282.0925, (*m/z*) found: $[C_{17}H_{12}FNO_2)Na]^+$ 282.0927.

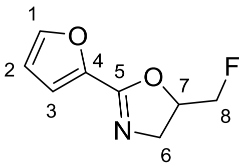

5-(Fluoromethyl)-2-(furan-2-yl)-4,5-dihydrooxazole (2n)

Prepared according to the general procedure from *N*-allylfuran-2-carboxamide (**1n**) (30 mg, 200 µmol) over 40 h. The product was purified by column chromatography (cyclohexane : ethyl acetate 2:1 to 1:1, dry load) and obtained as a colourless solid (20 mg, 59%).

R*f*: 0.21 (cyclohexane : ethyl acetate, 1:2).

M.p.: 73 °C.

FT-IR ($\tilde{\nu}$ = cm^{-1}): 3147 (w), 3129 (w), 3102 (w), 2973 (w), 2884 (w), 1665 (s), 1584 (w), 1561 (m), 1514 (w), 1479 (s), 1408 (m), 1358 (w), 1317 (m), 1271 (w), 1257 (w), 1241 (w), 1175 (s), 1103 (s), 1075 (w), 1060 (w), 1042 (w), 1019 (s), 1005 (s), 998 (s), 987 (s), 937 (m), 915 (w), 892 (w), 881 (m), 873 (m), 848 (s), 776 (s), 709 (s).

^{1}H NMR (600 MHz, $CDCl_3$, 298 K): δ [ppm] = 7.54 (d, $^{3}J_{HH}$ = 1.8 Hz, 1H, H-C3), 6.98 (d, $^{3}J_{HH}$ = 3.5 Hz, .1H, H-C1), 6.49 (dd, $^{3}J_{HH}$ = 3.5, 1.8 Hz, 1H, H-C2), 4.90 (ddddd, $^{3}J_{HF}$ = 19.6 Hz, $^{3}J_{HH}$ = 10.5, 7.5, 5.4, 3.0 Hz, 1H, H-C7), 4.59 (ddd, $^{2}J_{HF}$ = 47.0 Hz, $^{2}J_{HH}$ = 10.5 Hz, $^{3}J_{HH}$ = 3.0 Hz, 1H, H^a-C8), 4.49 (ddd, $^{2}J_{HF}$ = 47.0 Hz, $^{2}J_{HH}$ = 10.5 Hz, $^{3}J_{HH}$ = 5.5 Hz, 1H, H^b-C8), 4.15 (ddd, $^{2}J_{HH}$ = 14.8 Hz, $^{3}J_{HH}$ = 10.5 Hz, $^{4}J_{HF}$ = 1.5 Hz, 1H, H^a-C6), 3.87 (dd, $^{2}J_{HH}$ = 14.8 Hz, $^{3}J_{HH}$ = 7.5 Hz, 1H, H^b-C6).

^{13}C{^{1}H} NMR (151 MHz, $CDCl_3$, 298 K): δ [ppm] = 156.5 (C5), 145.5 (C3), 142.7 (C4), 114.8 (C1), 111.7 (C2), 83.2 (d, $^{1}J_{CF}$ = 175.7 Hz, C8), 78.0 (d, $^{2}J_{CF}$ = 19.8 Hz, C7), 55.8 (d, $^{3}J_{CF}$ = 6.0 Hz, C6).

^{19}F NMR (564 MHz, $CDCl_3$, 298 K): δ [ppm] = -229.4 (tdd, $^2J_{FH}$ = 47.0 Hz, $^3J_{FH}$ = 19.6 Hz, $^4J_{FH}$ = 1.5 Hz, F-C8).

ESI-MS: (*m/z*) required: $[(C_8H_8NO_2F)Na]^+$ = 192.0431, (*m/z*) found: $[(C_8H_8NO_2F)Na]^+$ = 192.0431.

1,3-Bis(5-(fluoromethyl)-4,5-dihydrooxazol-2-yl)benzene (2p)

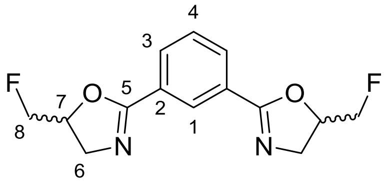

Prepared according to the general procedure from *N*,*N*-diallylisophthalimide (**1p**) (24 mg, 100 µmol) with *p*-iodotoluene (4.4 mg, 20 µmol, 20 mol %) and Selectfluor® (106 mg, 300 µmol, 3.0 equiv) in dichloromethane (1 mL) and amine:HF, 1:4.5 (1 mL) over 32 h. The product was purified by column chromatography (cyclohexane : ethyl acetate, 1:5). The product was obtained as a colourless solid (13 mg, 46 µmol, 46%). The diastereoisomeric ratio was determined by ^{19}F NMR to be 1:1. When possible the diastereoisomers are marked as follows: **2p** and **2p'**.

R*f*: 0.21 (cyclohexane : ethyl acetate, 1:5).

M.p.: 95 °C.

FT-IR ($\tilde{\nu}$ = cm^{-1}): 3050 (w), 2954 (w), 2871 (w), 1727 (w), 1650 (s), 1598 (w), 1492 (w), 1461 (w), 1407 (w), 1378 (m), 1341 (m), 1308 (m), 1271 (m), 1235 (m), 1191 (w), 1108 (m), 1078 (m), 1059 (s), 999 (s), 982 (s), 944 (m), 910 (m), 885 (m), 855 (s), 830 (s), 738 (m), 698 (s), 664 (s).

^{1}H NMR (600 MHz, $CDCl_3$, 298 K): δ [ppm] = 8.51 (t, $^4J_{HH}$ = 1.7 Hz, 2H, H-C1), 8.09 (dd, $^3J_{HH}$ = 7.8 Hz, $^4J_{HH}$ = 1.7 Hz, 4H, H-C3), 7.48 (t, $^3J_{HH}$ = 7.8 Hz, 2H, H-C4), 4.98–4.90 (m, 4H, H-C7), 4.62 (dd, $^2J_{HF}$ = 47.3 Hz, $^2J_{HH}$ = 10.5 Hz, $^3J_{HH}$ = 2.9 Hz, 2H, H^a-C8), 4.61 (dd, $^2J_{HF}$ = 47.1 Hz, $^2J_{HH}$ = 10.4 Hz, $^3J_{HH}$ = 2.8 Hz, 2H, H^a-C8'), 4.52 (ddd, $^2J_{HF}$ = 47.1 Hz, $^2J_{HH}$ = 10.5, $^3J_{HH}$ = 2.8 Hz, 2H, H^b-C8), 4.51 (ddd, $^2J_{HF}$ = 47.1 Hz, $^2J_{HH}$ = 10.4 Hz, $^3J_{HH}$ = 2.7 Hz, 2H, H^b-C8'), 4.18 (d, $^2J_{HH}$ = 14.9 Hz, $^4J_{HF}$ = 1.4 Hz, 2H, H^a-C6), 4.16 (d, $^2J_{HH}$ = 14.9 Hz, $^4J_{HF}$ = 1.5 Hz, 2H, H^a-C6'), 3.92 (dd, $^2J_{HH}$ = 14.9 Hz, $^3J_{HH}$ = 7.7 Hz, 2H, H^b-C6), 3.91 (dd, $^2J_{HH}$ = 14.9 Hz, $^3J_{HH}$ = 7.7 Hz, 2H, H^b-C6').

^{13}C{^{1}H} NMR (151 MHz, $CDCl_3$, 298 K): δ [ppm] = 163.5 (C5), 131.3 (C3), 128.7 (C4), 128.2 (C1), 127.9 (C2), 83.4 (d, $^1J_{CF}$ = 175.4 Hz, C8), 78.0 (d, $^2J_{CF}$ = 18.6 Hz, C7), 56.0 (d, $^3J_{CF}$ = 5.9 Hz, C6).

^{19}F{^{1}H} NMR (564 MHz, $CDCl_3$, 298 K): δ [ppm] = -229.65 (s, 1F, F-C8), -229.66 (s, 1F, F-C8').

^{19}F NMR (564 MHz, $CDCl_3$, 298 K): δ [ppm] = -229.65 (tdd, $^2J_{FH}$ = 47.2 Hz, $^3J_{FH}$ = 20.0 Hz, $^4J_{FH}$ = 1.5 Hz, 1F, F-C8), -229.66 (tdd, $^2J_{FH}$ = 47.1 Hz, $^3J_{FH}$ = 20.0 Hz, $^4J_{FH}$ = 1.5 Hz, 1F, F-C8').

ESI-MS: (*m/z*) required: $[C_{14}H_{14}N_2O_2F)H]^+$ = 281.1096, (*m/z*) found: $[C_{14}H_{14}N_2O_2F)H]^+$ = 281.1101.

Benzyl 3-(5-(fluoromethyl)-4,5-dihydrooxazol-2-yl)propanoate (2q)

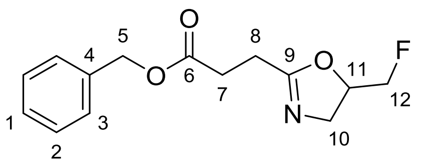

Prepared according to the general procedure from benzyl 4-(allylamino)-4-oxobutanoate (**1q**) (49 mg, 200 µmol) over 26 h. The product was purified by column chromatography (cyclohexane : ethyl acetate, 1:1) and obtained as a colourless oil (23 mg, 87 µmol, 44%).

R*f*: 0.26 (cyclohexane : ethyl acetate, 1:2).

FT-IR ($\tilde{\nu}$ = cm^{-1}): 2959 (w), 2923 (w), 2854 (w), 1733 (m), 1674 (m), 1498 (w), 1456 (w), 1381 (w), 1354 (w), 1212 (m), 1154 (s), 1083 (m), 1004 (s), 909 (m), 852 (m), 791 (s), 736 (s).

^{1}H NMR (400 MHz, $CDCl_3$, 298 K): δ [ppm] = 7.38–7.29 (m, 5H, H-C1, H-C2, H-C3), 5.14 (s, 2H, H-C5), 4.70 (ddddd $^3J_{HF}$ = 19.9 Hz, $^3J_{HH}$ =10.3, 7.5, 5.5, 3.0 Hz, 1H, H-C11), 4.44 (ddd, $^2J_{HF}$ =47.2 Hz, $^2J_{HH}$ = 10.4 Hz, $^3J_{HH}$ = 3.0 Hz, 1H, H^a-C12), 4.35 (ddd, $^2J_{HF}$ =47.2 Hz, ^2J_H = 10.4 Hz, $^3J_{HH}$ = 5.5 Hz, 1H, H^b-C12), 3.87 (ddq, $^2J_{HH}$ = 14.7 Hz, $^3J_{HH}$ = 10.2 Hz, $^4J_{HF}$ = 1.5 Hz, 1H, H^a-C10), 3.60 (ddt, $^2J_{HH}$ = 14.7 Hz, $^3J_{HH}$ = 7.3 Hz, $^4J_{HF}$ = 1.5 Hz, 1H, H^b-C10), 2.77–2.70 (m, 2H, H-C8), 2.66–2.60 (m, 2H, H-C7).

$^{13}C\{^1H\}$ NMR (101 MHz, $CDCl_3$, 298 K): δ [ppm] = 172.1 (C6), 166.6 (C9), 136.0 (C4), 128.7–128.4 (C1, C2, C3), 84.3 (d, $^1J_{CF}$ = 174.9 Hz, C12), 77.5 (d, $^2J_{CF}$ = 32.8 Hz, C11), 66.6 (C5), 55.3 (d, $^3J_{CF}$ = 5.8 Hz, C10), 30.4 (C8), 23.3 (C7).

^{19}F NMR (282 MHz, $CDCl_3$, 298 K): δ [ppm] = -229.4 (tdd, $^2J_{HF}$ = 47.0 Hz, $^3J_{HF}$ = 19.8 Hz, $^4J_{HF}$ = 1.5 Hz, F-C9).

ESI-MS: (*m/z*) requires: $[(C_{14}H_{16}FNO_3)H]^+$ = 266.1187, (*m/z*) found: $[(C_{14}H_{16}FNO_3)H]^+$ = 266.1192.

(2*S*)-2-(Benzyloxy)-*N*-(3-fluoro-2-hydroxypropyl)-3-phenylpropanamide (3)

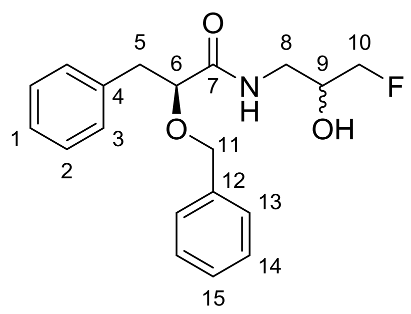

Prepared according to the general procedure from (*S*)-*N*-allyl-2-(benzyloxy)-3-phenylpropanamide (**1r**) (59 mg, 200 µmol) over 32 h. The analysis of the crude product by ^{19}F NMR gave >95% yield and a diastereoisomeric ratio of 1:1. Due to hydrolysis of the oxazoline product **2r** on silica, for isolation the crude product was dissolved in an aqueous solution of HCl (0.5 mL, 1 M) and stirred at ambient temperature for 14 h. The mixture was subsequently poured into an aqueous saturated solution of $NaHCO_3$ (10 mL) and was extracted with ethyl acetate (3 × 5 mL). The combined organic phases were dried over $MgSO_4$ and the solvent was removed in vacuo. The residue was purified by column chromatography (cyclohexane : ethyl acetate, 2:1) and the product (40 mg, 121 µmol, 61%) was obtained as a colourless solid. The diastereoisomeric ratio was determined by ^{19}F NMR to be 1:1. Where possible the diastereoisomers are marked as follows: **3** and **3’**.

R_f: 0.21 (cyclohexane : ethyl acetate, 1:5).

M.p.: 74 °C.

FT-IR ($\tilde{\nu}$ = cm^{-1}): 3305 (w), 3063 (w), 3033 (w), 2924 (w), 1635 (m), 1537 (s), 1496 (m), 1455 (m), 1434 (w), 1371 (w), 1333 (w), 1283 (w), 1257 (m), 1209 (w), 1123 (m), 1091 (s), 1082 (s), 998 (s), 952 (m), 910 (w), 889 (w), 841 (w), 746 (s), 695 (s), 616 (s).

^{1}H NMR (600 MHz, $CDCl_3$, 298 K): δ [ppm] = 7.36–7.28 (m, 10H, H-C^{Ar}), 7.27–7.20 (m, 10H, H-C^{Ar}), 6.89–6.81 (m, 2H, NH), 4.51 (d, $^4J_{HH}$ = 2.2 Hz, 2H, H-C11), 4.48 (d, $^4J_{HH}$ = 2.6 Hz, 2H, H-C11'), 4.31–4.12 (m, 4H, H-C10), 4.18–4.12 (m, 2H, H-C6), 3.87–3.74 (m, 2H, H-C9), 3.49 (dddd, $^2J_{HH}$ = 14.2 Hz, $^3J_{HH}$ = 6.7, 3.4 Hz, $^4J_{HF}$ = 1.2 Hz, 1H, H^a-C8), 3.40 (dddd, $^2J_{HH}$ = 14.3 Hz, $^3J_{HH}$ = 6.3, 3.7 Hz, $^4J_{HF}$ = 1.3 Hz, 1H, H^a-C8'), 3.33–3.27 (m, 1H, H^b-C8), 3.25–3.16 (m, 2H, H^a-C5), 3.19–3.12 (m, 1H, H^b-C8'), 3.03 (br-s, 1H, OH), 3.02–2.95 (m, 2H, H^b-C5).

^{13}C{^{1}H} NMR (151 MHz, $CDCl_3$, 298 K): δ [ppm] = 173.7 (C7), 173.5 (C7'), 137.0 (C^{Ar}), 136.97 (C^{Ar}), 136.96 (C^{Ar}), 130.1 (C^{Ar}), 129.9 (C^{Ar}), 128.8 (C^{Ar}), 128.7 (C^{Ar}), 128.41 (C^{Ar}), 128.4 (C^{Ar}), 128.35 (C^{Ar}), 128.1 (C^{Ar}), 128.07 (C^{Ar}), 126.9 (C^{Ar}), 126.88 (C^{Ar}), 84.3 (d, $^1J_{CF}$ = 169.5 Hz, C10'), 84.2 (d, $^1J_{CF}$ = 169.7 Hz, C10'), 80.7 (C6), 80.5 (C6'), 73.2 (C11), 73.1 (C11'), 69.9 (d, $^2J_{CF}$ = 20.3 Hz, C9), 69.7 (d, $^2J_{CF}$ = 20.4 Hz, C9'), 41.8 (d, $^3J_{CF}$ = 6.4 Hz, C8), 41.76 (d, $^3J_{CF}$ = 6.6 Hz, C8'), 38.9 (C5), 38.7 (C5').

^{19}F{^{1}H} NMR (564 MHz, $CDCl_3$, 298 K): δ [ppm] = -230.6 (s, 1F, F-C10), -231.0 (s, 1F, F-C10').

^{19}F NMR (564 MHz, $CDCl_3$, 298 K): δ [ppm] = -230.6 (tdt, $^2J_{FH}$ = 47.0 Hz, $^3J_{FH}$ = 15.9 Hz, $^4J_{FH}$ = 1.0 Hz, 1F, F-C10), -231.0 (tdt, $^2J_{FH}$ = 47.0 Hz, $^3J_{FH}$ = 16.5 Hz, $^4J_{FH}$ = 0.9 Hz, 1F, F-C10').

ESI-MS: (*m/z*) required: $[C_{19}H_{22}NO3F)Na]^+$ = 354.1476, (*m/z*) found: $[C_{19}H_{22}NO3F)Na]^+$ = 354.1480.

(4*S*,5*R*)-4-Benzyl-5-(fluoromethyl)-2-phenyl-4,5-dihydrooxazole (2s)

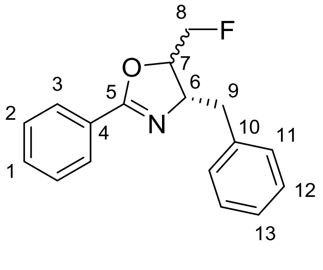

Prepared according to the general procedure from (S)-*N*-(1-phenylbut-3-en-2yl)benzamide (**1s**) (50 mg, 200 µmol) over 72 h at 40 °C. The product was purified by column chromatography (cyclohexane : ethyl acetate, 7:1) and obtained as a yellow oil (35 mg, 130 µmol, 65%). The diastereomeric ratio was determined from the crude ^{19}F{^{1}H} NMR spectrum to be > 95:5.

R_f: 0.19 (cyclohexane : ethyl acetate, 7:1).

ORD (CH_2Cl_2, c 1.00): $[\boldsymbol{\alpha}]_{\boldsymbol{D}}^{\boldsymbol{25}}$ = -13.4°.

FT-IR ($\widetilde{\nu}$ = cm^{-1}): 3028 (w), 2928 (w), 1723 (w), 1651 (s), 1603 (w), 1580 (w), 1494 (m), 1451 (m), 1333 (w), 1316 (w), 1271 (w), 1177 (w), 1083 (m), 1062 (m), 1048 (m), 1023 (s), 982 (m), 908 (m), 884 (m), 856 (w), 779 (m), 757 (w), 730 (s), 691 (s).

^{1}H NMR (600 MHz, $CDCl_3$, 298 K): δ [ppm] = 8.00–8.96 (m, 2H, H-C3), 7.52–7.48 (m, 1H, H-C1), 7.45–7.40 (m, 2H, H-C2), 7.35–7.31 (m, 2H, H-C12), 7.28–7.23 (m, 3H, H-C11, H-C13), 4.53 (dddd, $^3J_{HF}$ = 20.2 Hz, $^3J_{HH}$ = 7.1, 5.8, 2.7 Hz, 1H, H-C7), 4.32 (ddd, $^3J_{HH}$ = 9.2, 7.1, 5.3 Hz, 1H, H-C6), 4.26 (ddd, $^2J_{HF}$ = 47.2 Hz, $^2J_{HH}$ = 10.5 Hz, $^3J_{HH}$ = 2.7 Hz, 1H, H^a-C8), 4.16 (ddd, $^2J_{HF}$ = 47.2 Hz, $^2J_{HH}$ = 10.5 Hz, $^3J_{HH}$ = 5.8 Hz, 1H, H^b-C8), 3.35 (dd, $^2J_{HH}$ = 13.6 Hz, $^3J_{HH}$ = 5.3 Hz, 1H, H^a-C9), 2.75 (dd, $^2J_{HH}$ = 13.6 Hz, $^3J_{HH}$ = 9.2 Hz, 1H, H^b-C9).

^{13}C{^{1}H} NMR (151 MHz, $CDCl_3$, 298 K): δ [ppm] = 163.5 (C5), 137.1 (C10), 131.7 (C1), 129.4 (C11), 128.9 (C12), 128.50 (C2), 128.49 (C3), 127.5 (C4), 127.0 (C13), 83.6 (d, $^1J_{CF}$ = 175.4 Hz, C8), 82.3 (d, $^2J_{CF}$ = 18.5 Hz, C7), 68.9 (d, $^3J_{CF}$ = 5.0 Hz, C6), 41.7 (C9).

^{19}F{^{1}H} NMR (564 MHz, $CDCl_3$, 298 K): δ [ppm] = -227.9 (s, F-C8).

^{19}F NMR (564 MHz, $CDCl_3$, 298 K): δ [ppm] = -227.9 (td, $^2J_{FH}$ = 47.2 Hz, $^3J_{FH}$ = 20.2 Hz, F-C8).

ESI-MS: (*m/z*) required: $[C_{17}H_{16}NOF)H]^+$ = 270.1289, (*m/z*) found: $[C_{17}H_{16}NOF)H]^+$ = 270.1292.

Selected NMR spectra

N-Allyl-4-(trifluoromethyl)benzamide (1d)

^{1}H NMR (600 MHz, $CDCl_3$, 298 K)

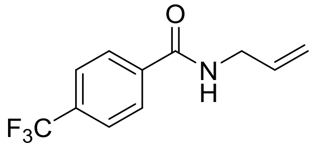

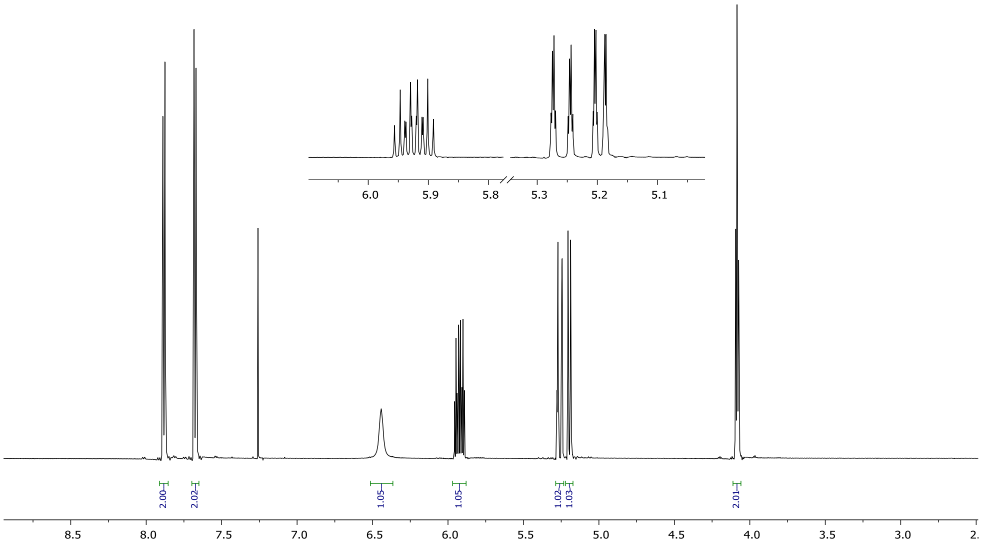

^{13}C{^{1}H} NMR (151 MHz, $CDCl_3$, 298 K)

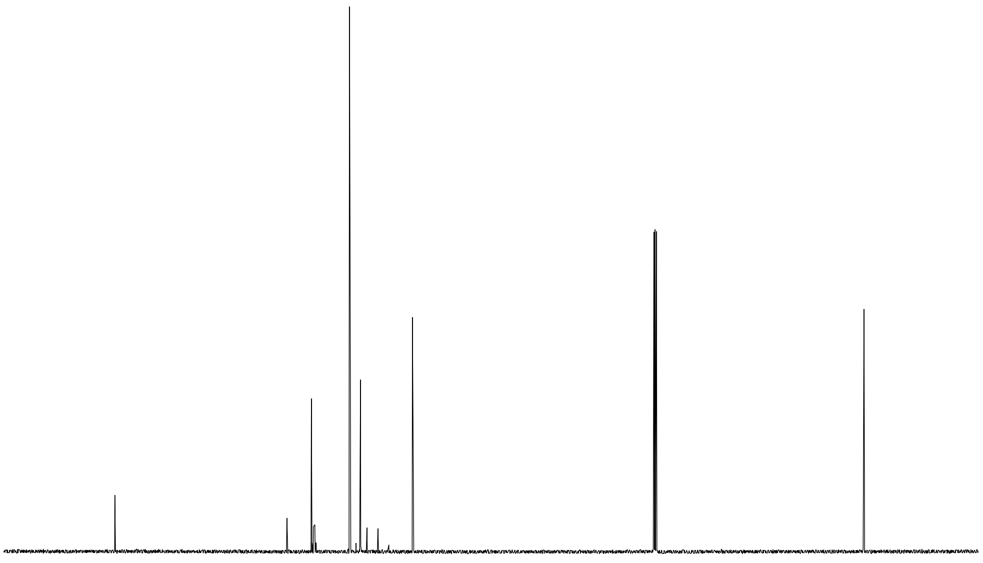

^{19}F{^{1}H} NMR (564 MHz, $CDCl_3$, 298 K)

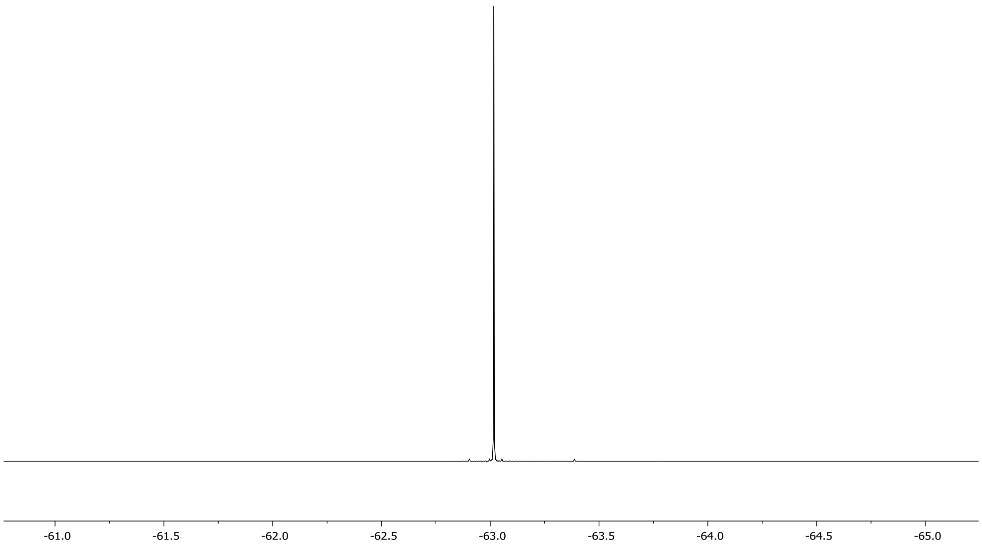

2-Acetamido-*N*-allylbenzamide (1g)

^{1}H NMR (400 MHz, $CDCl_3$, 298 K)

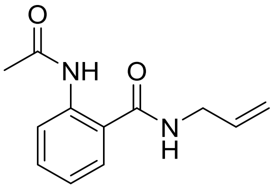

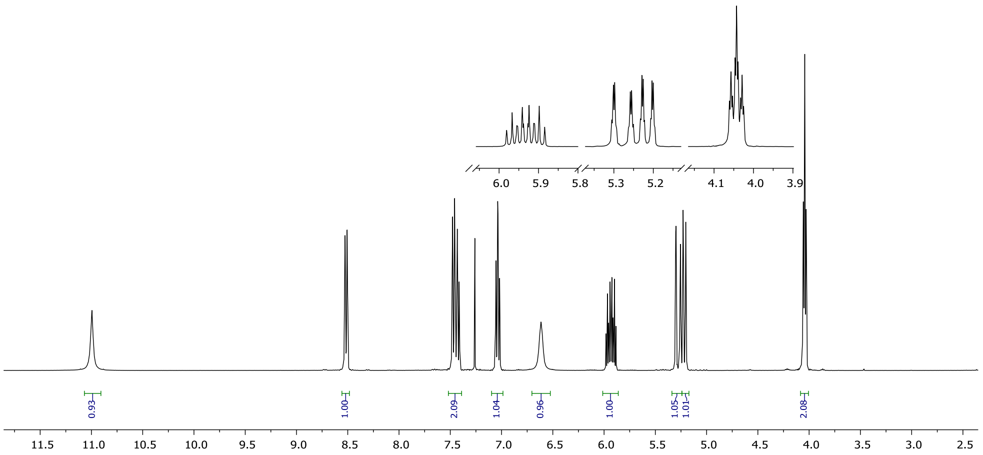

^{13}C{^{1}H} NMR (101 MHz, $CDCl_3$, 298 K)

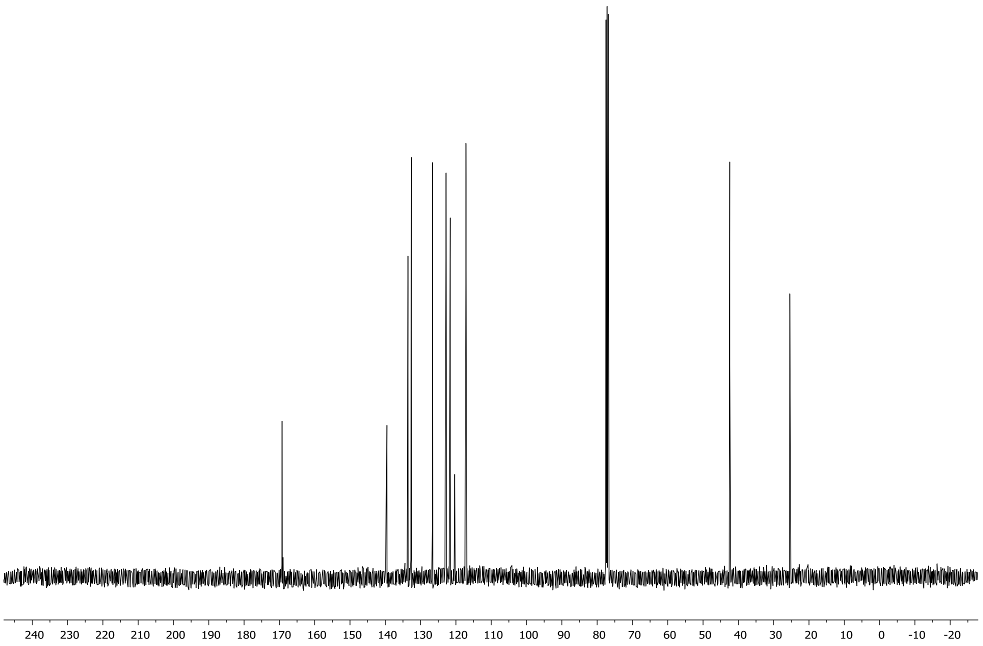

N-Allyl-2-hydroxy-5-nitrobenzamide (1i)

^{1}H NMR (600 MHz, $CDCl_3$, 298 K)

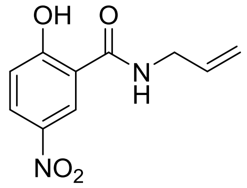

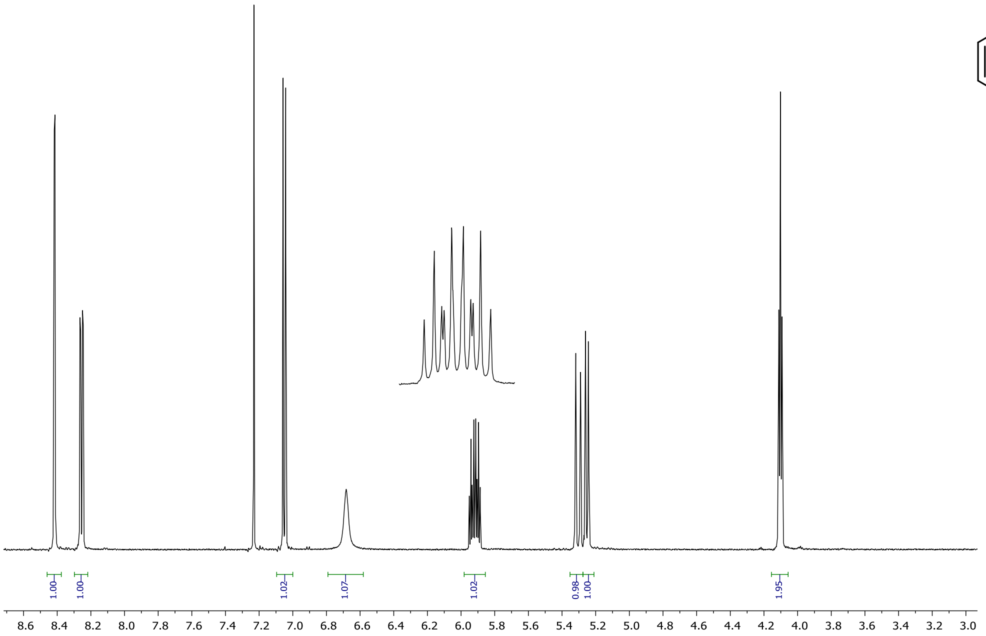

^{13}C{^{1}H} NMR (151 MHz, $CDCl_3$, 298 K)

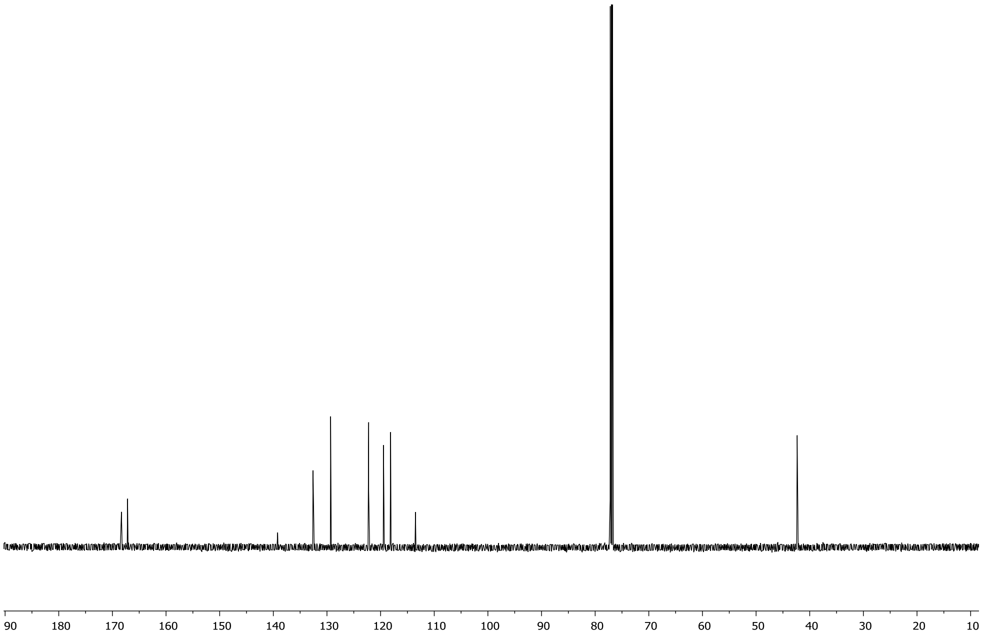

***N*-Allyl-2,3,4,5,6-pentafluorobenzamide (1j)**

^{1}H NMR (400 MHz, $CDCl_3$, 298 K)

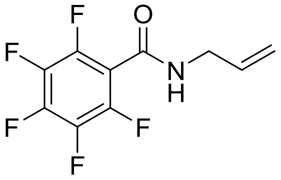

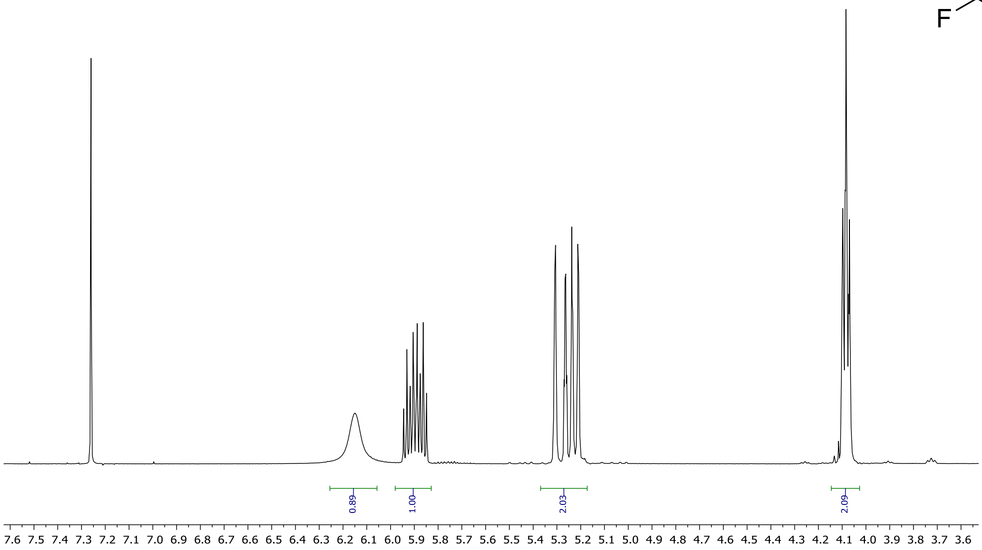

^{13}C{^{1}H} NMR (101 MHz, $CDCl_3$, 298 K)

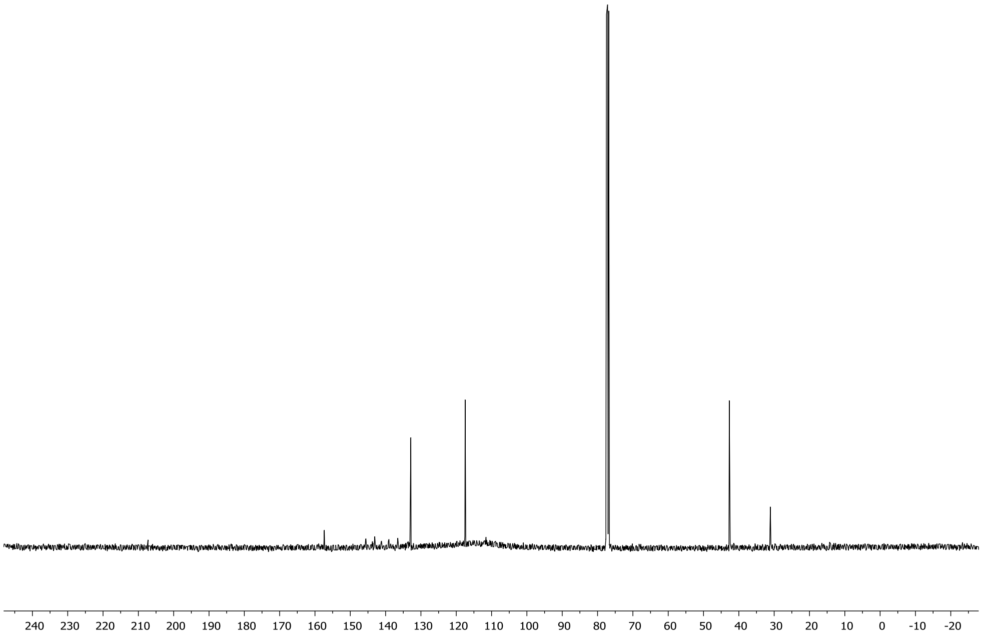

N-Allyl-9-oxo-9*H*-fluorene-4-carboxamide (1m)

^{1}H NMR (400 MHz, $CDCl_3$, 298 K)

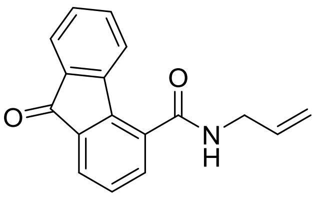

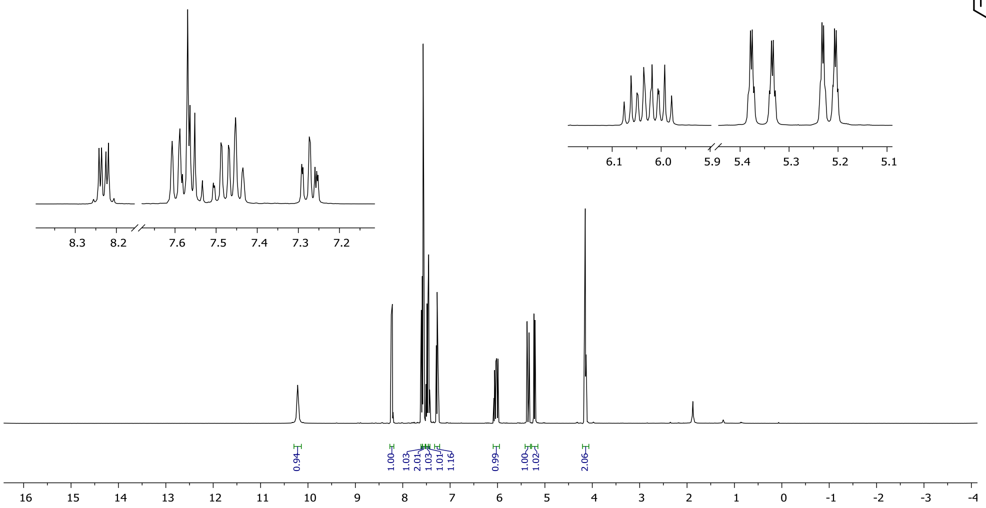

^{13}C{^{1}H} NMR (101 MHz, $CDCl_3$, 298 K)

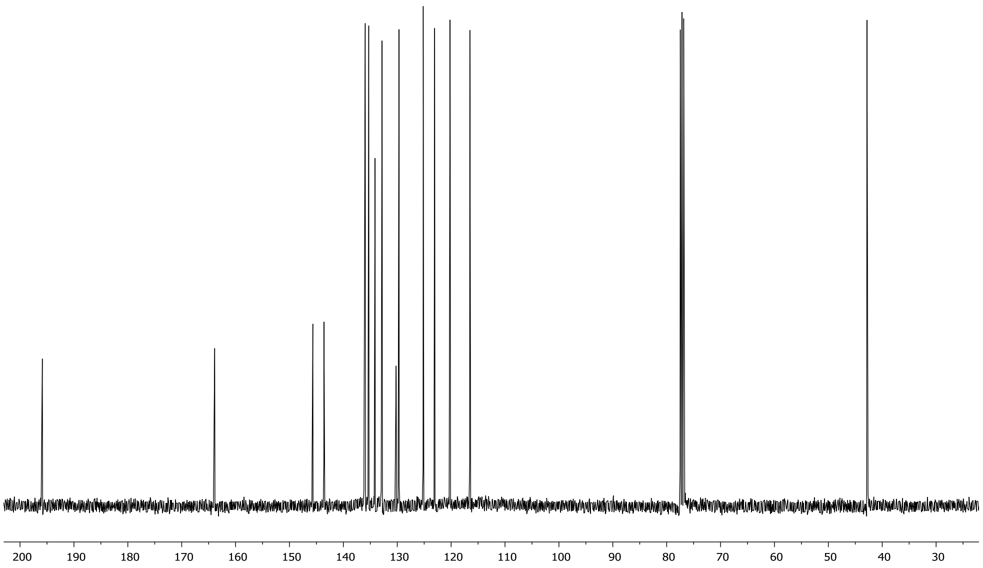

Benzyl 4-(allylamino)-4-oxobutanoate (1q)

^{1}H NMR (600 MHz, $CDCl_3$, 298 K)

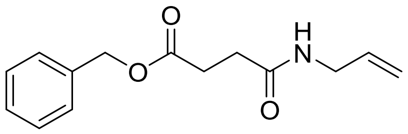

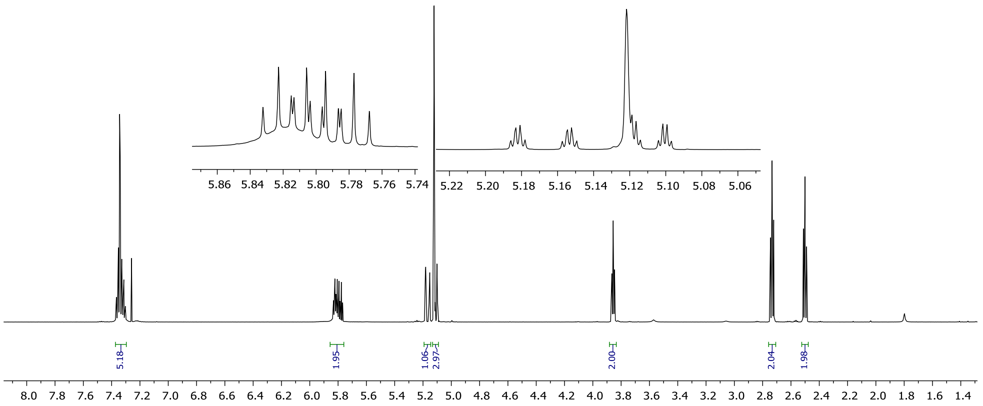

^{13}C{^{1}H} NMR (151 MHz, $CDCl_3$, 298 K)

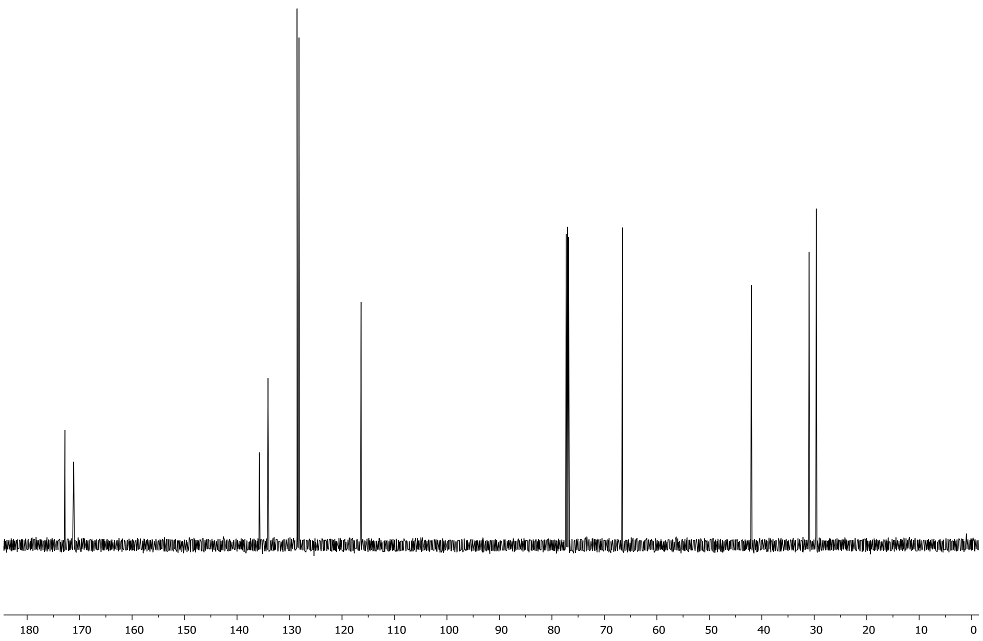

^{19}**F NMR** (282 MHz, $CDCl_3$, 298 K)

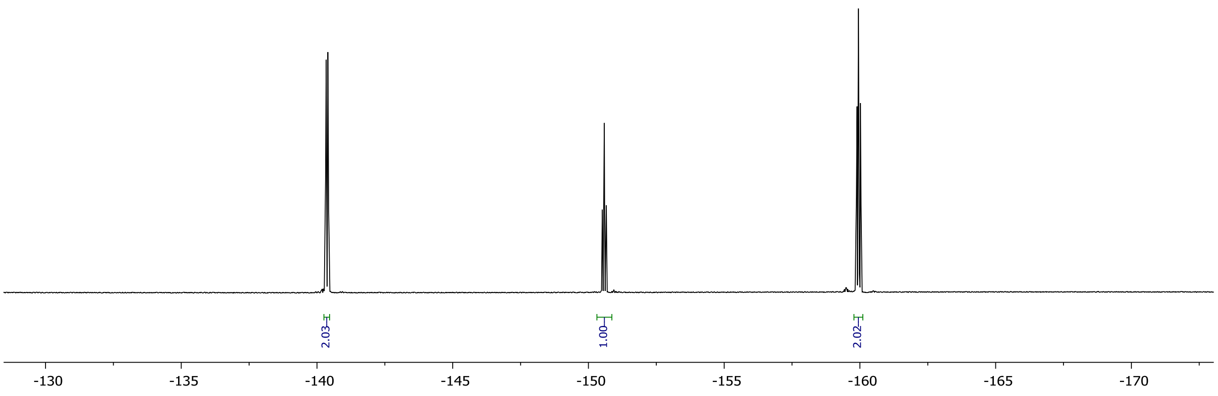

(*S*)-*N*-Allyl-2-hydroxy-3-phenylpropanamide (S4)

^{1}H NMR (400 MHz, $CDCl_3$, 298 K)

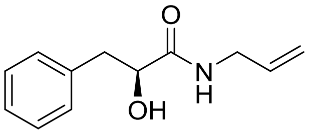

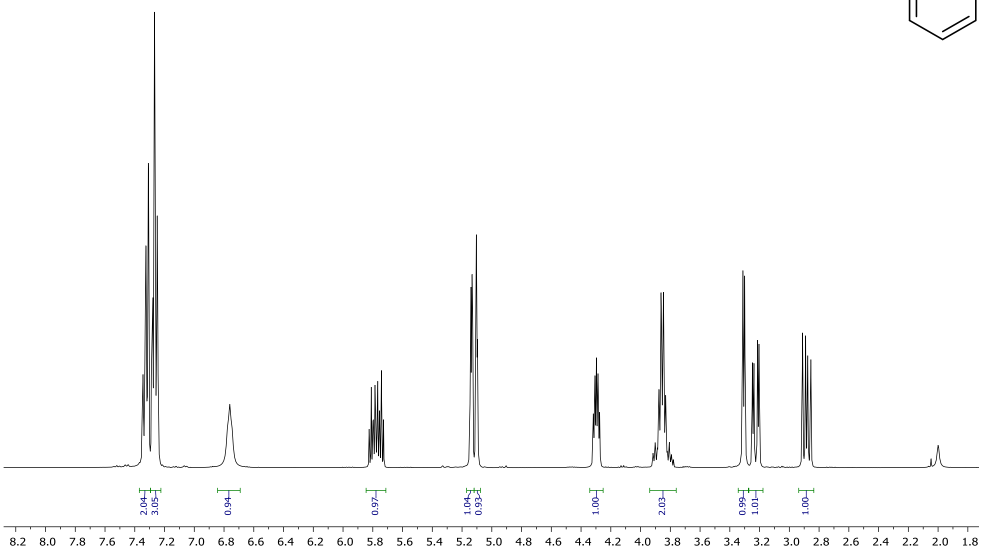

^{13}C{^{1}H} NMR (101 MHz, $CDCl_3$, 298 K)

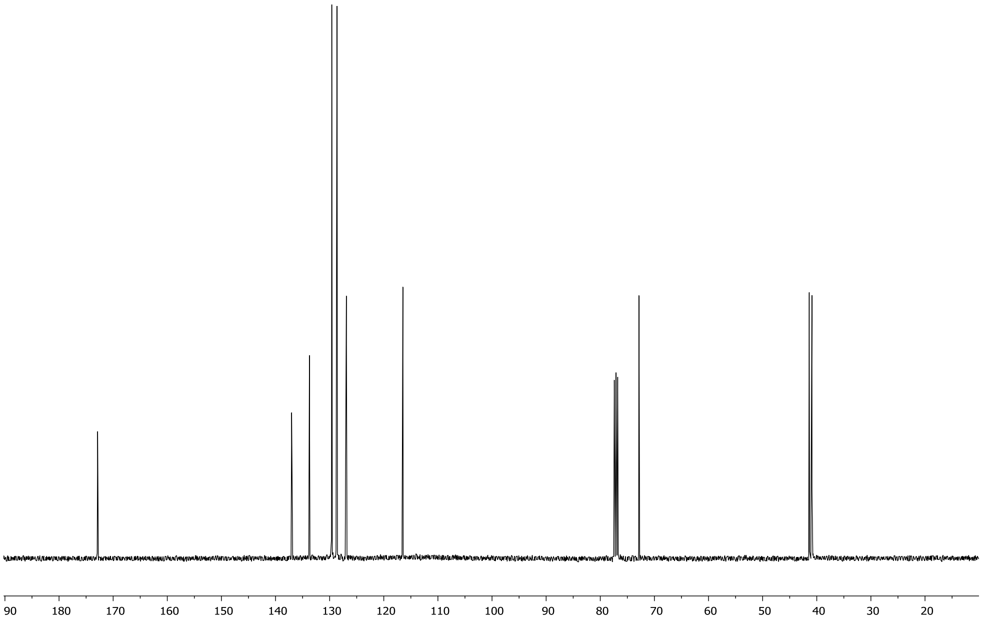

(*S*)-*N*-Allyl-2-(benzyloxy)-3-phenylpropanamide (1r)

^{1}H NMR (400 MHz, $CDCl_3$, 298 K)

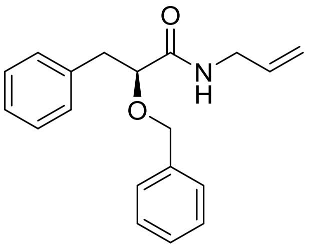

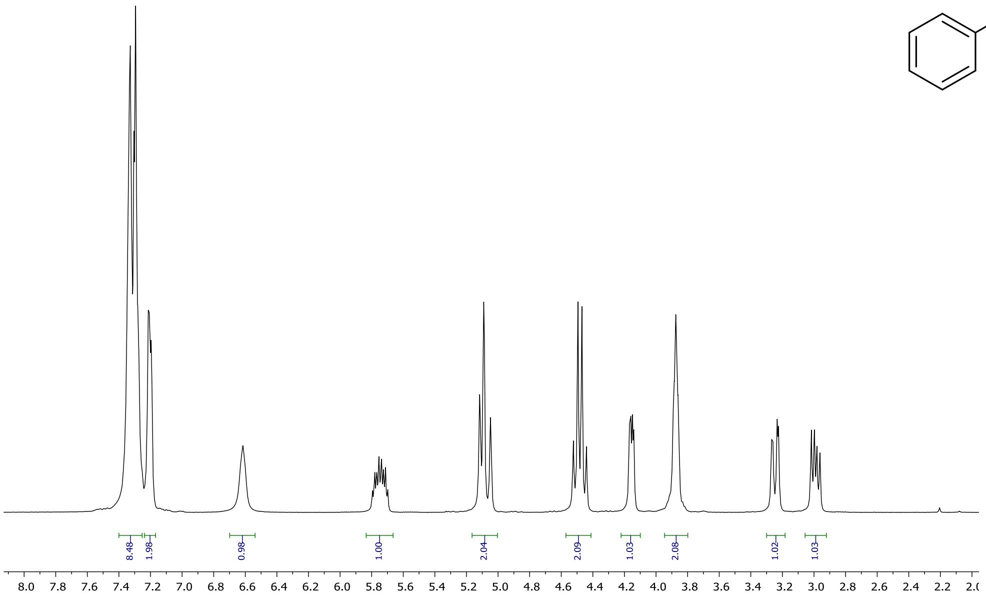

^{13}C{^{1}H} NMR (101 MHz, $CDCl_3$, 298 K)

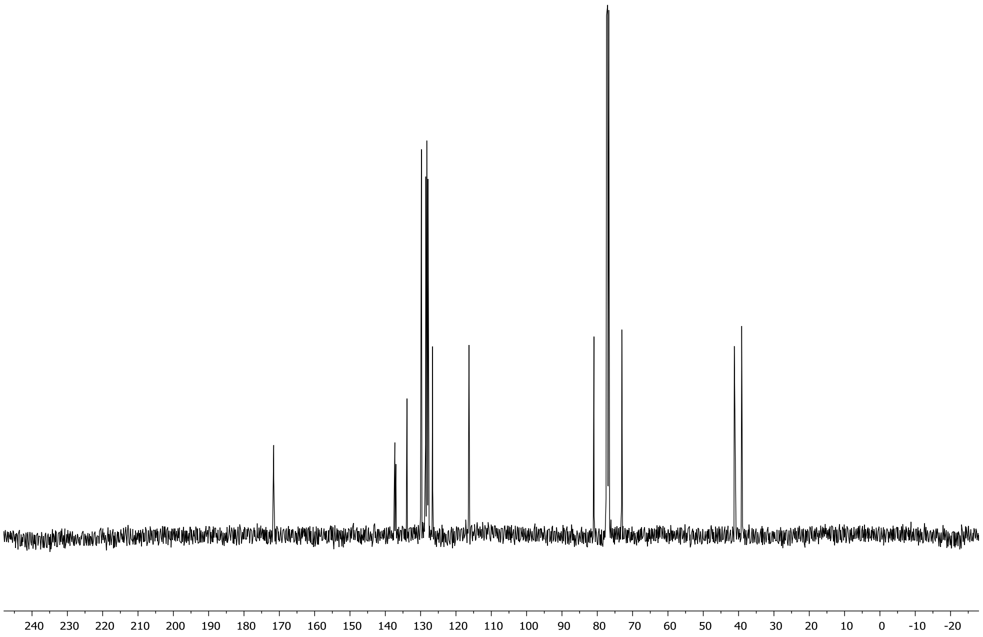

(*S*)-*N*-(1-Phenylbut-3-en-2-yl)benzamide (1s)

^{1}H NMR (600 MHz, $CDCl_3$, 298 K)

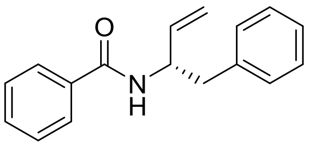

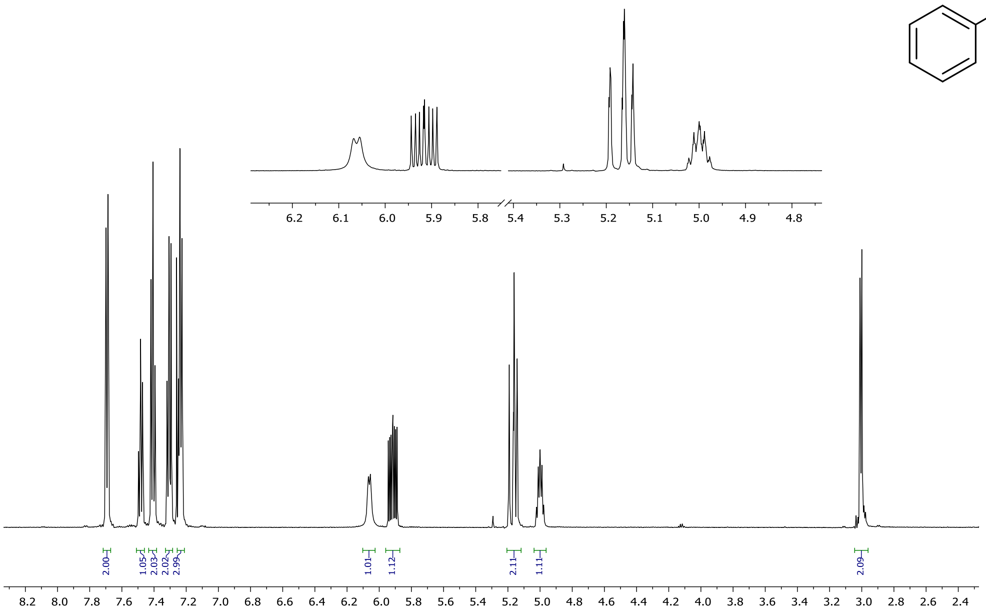

^{13}C{^{1}H} NMR (151 MHz, $CDCl_3$, 298 K)

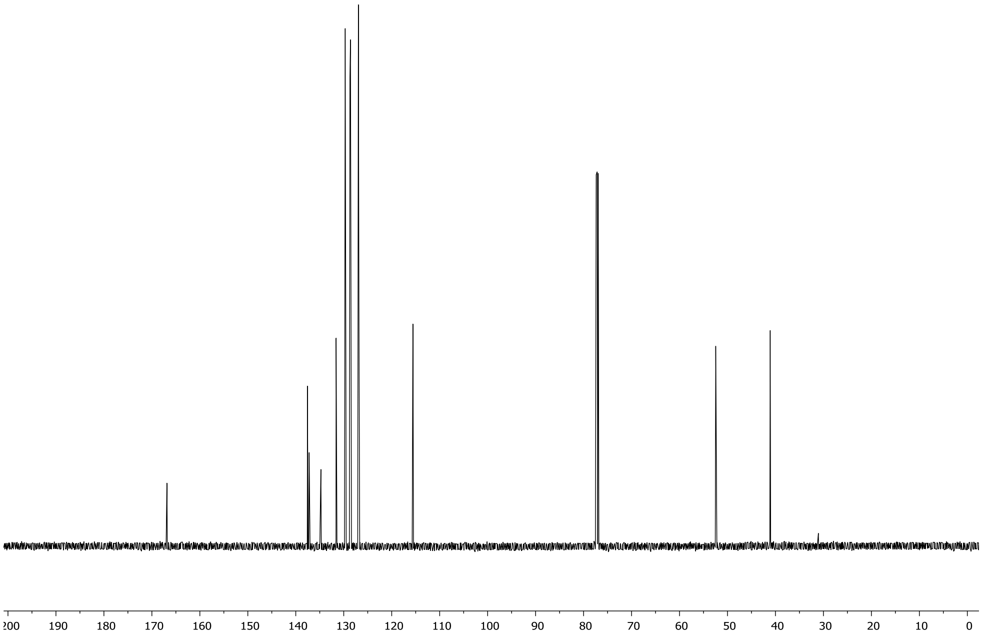

5-(Fluoromethyl)-2-phenyl-4,5-dihydrooxazole (2a)

^{1}H NMR (400 MHz, $CDCl_3$, 298 K)

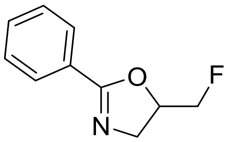

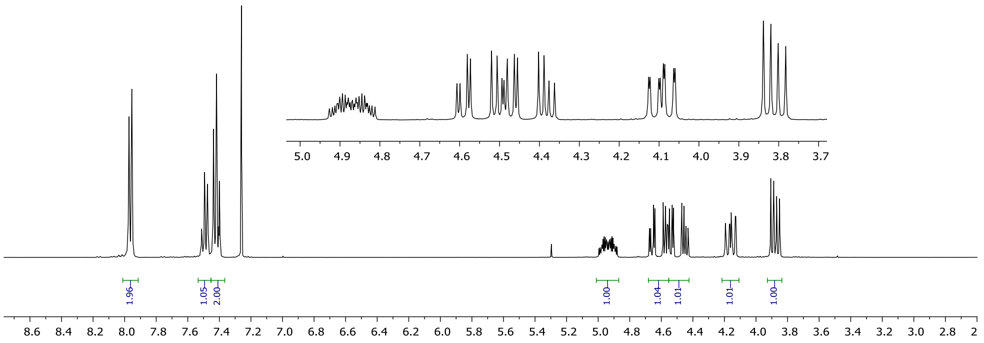

^{13}C{^{1}H} NMR (101 MHz, $CDCl_3$, 298 K)

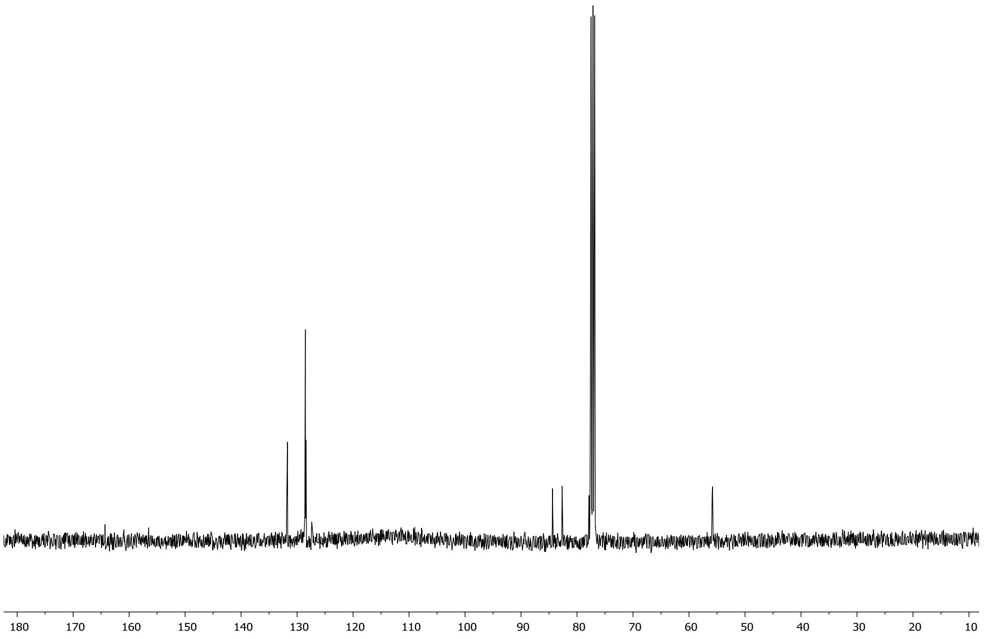

^{19}F NMR (282 MHz, $CDCl_3$, 298 K)

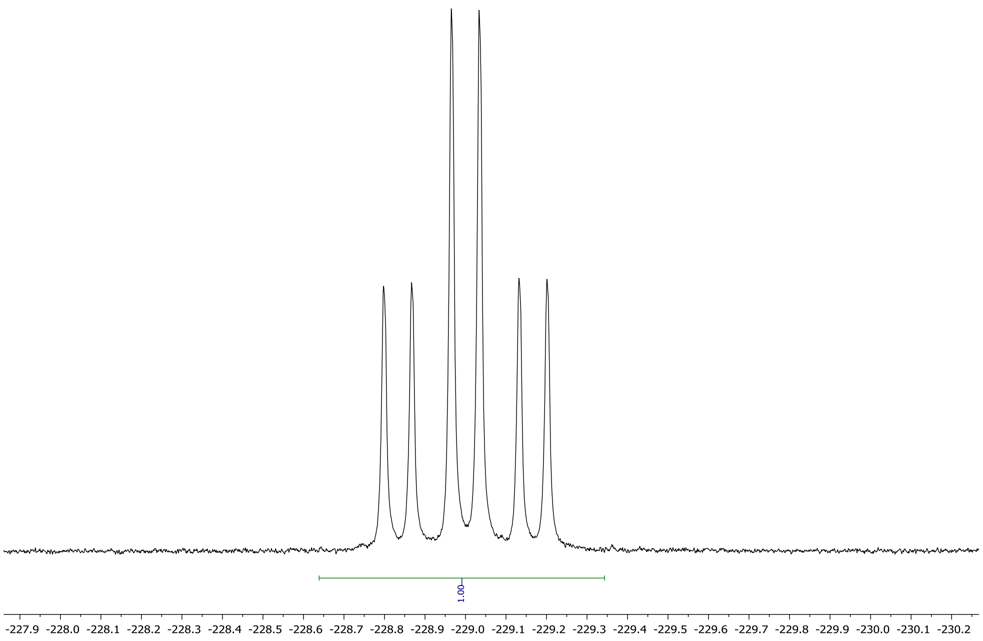

5-(Fluoromethyl)-2-(4-methoxyphenyl)-4,5-dihydrooxazole (2b)

^{1}H NMR (400 MHz, $CDCl_3$, 298 K)

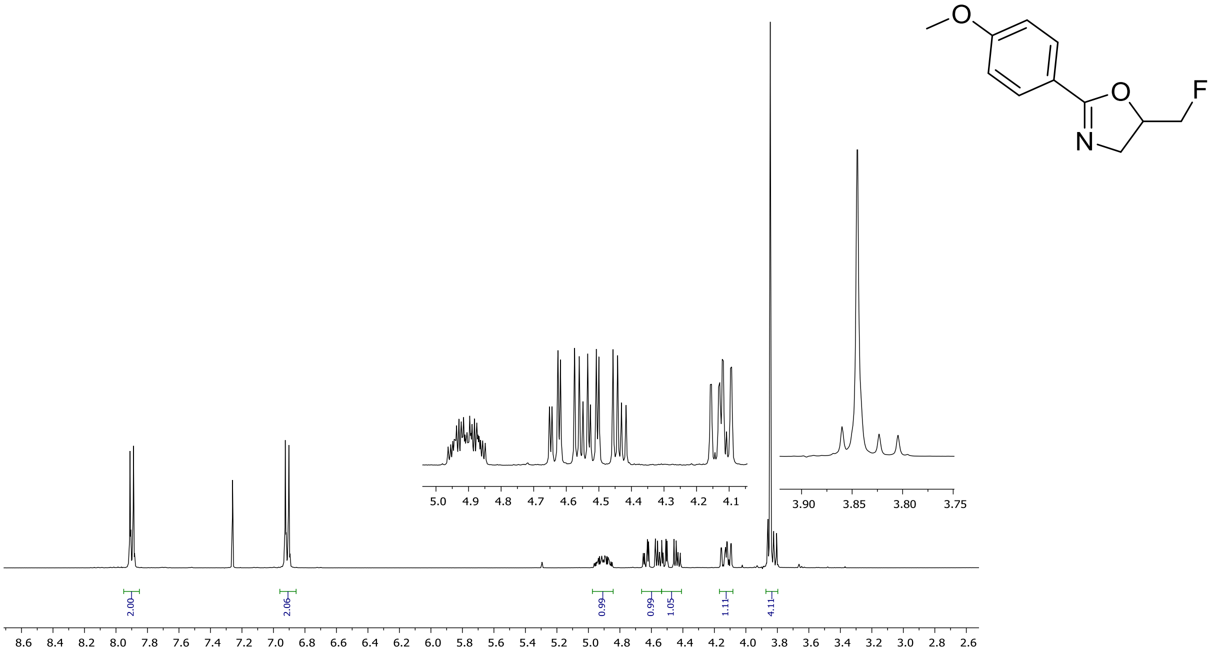

^{13}C{^{1}H} NMR (101 MHz, $CDCl_3$, 298 K)

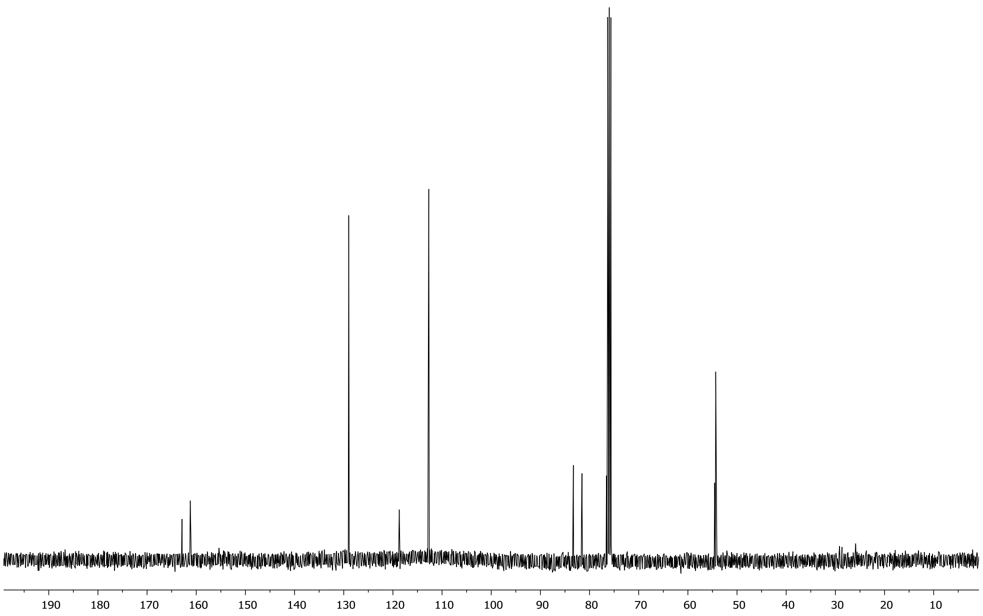

^{19}F NMR (282 MHz, $CDCl_3$, 298 K)

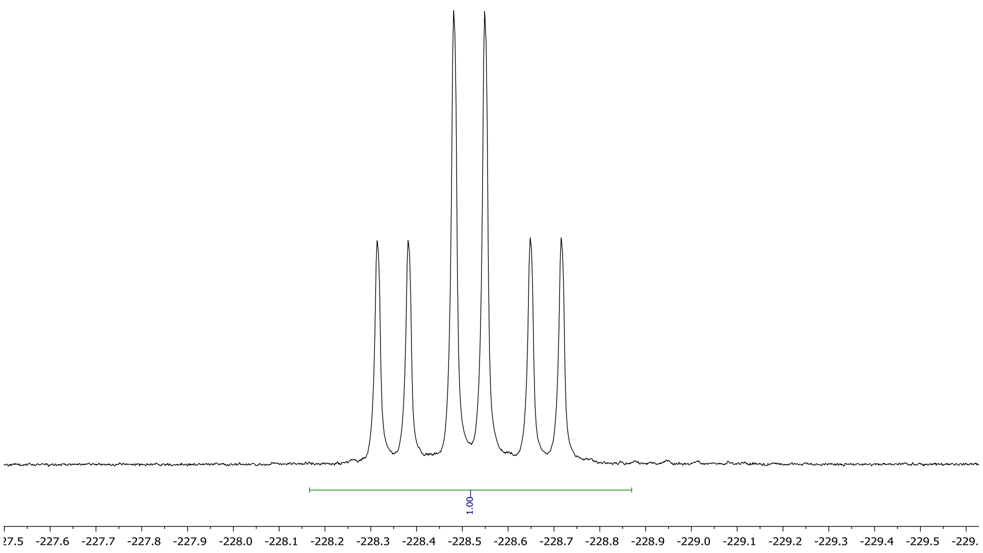

5-(Fluoromethyl)-2-(4-nitrophenyl)-4,5-dihydrooxazole (2c)

^{1}H NMR (400 MHz, $CDCl_3$, 298 K)

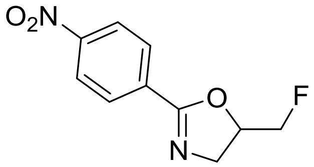

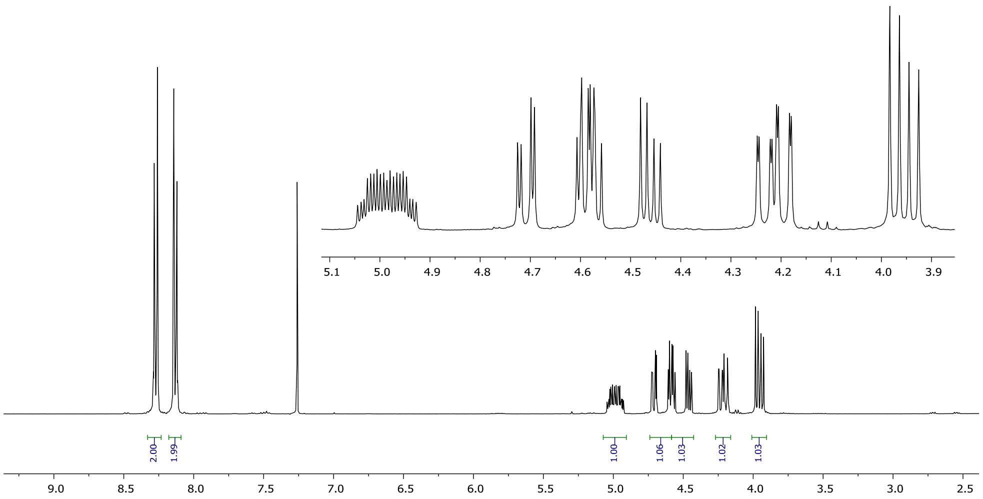

^{13}C{^{1}H} NMR (101 MHz, $CDCl_3$, 298 K)

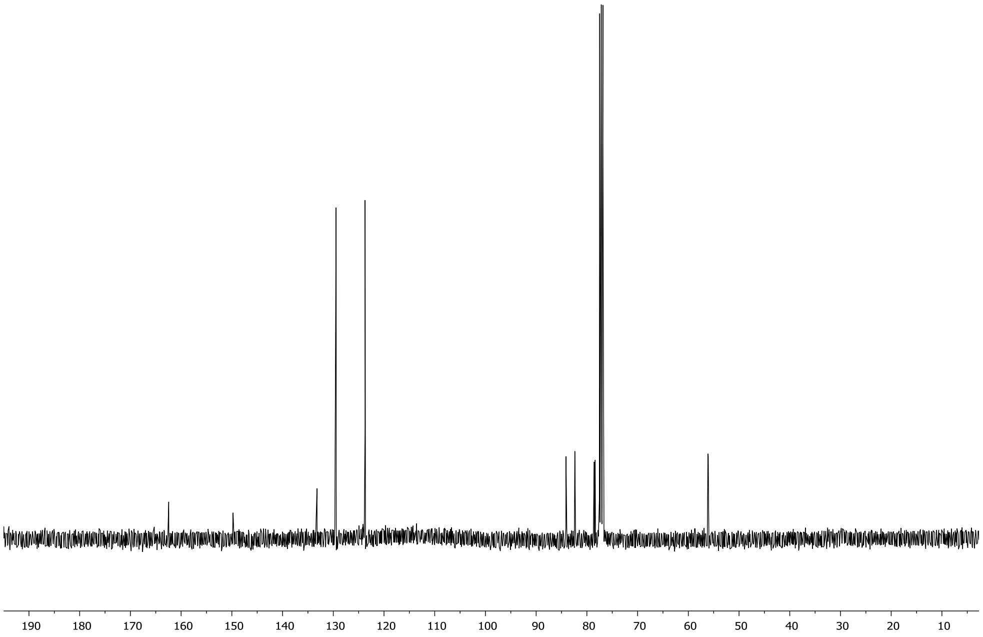

^{19}F NMR (282 MHz, $CDCl_3$, 298 K)

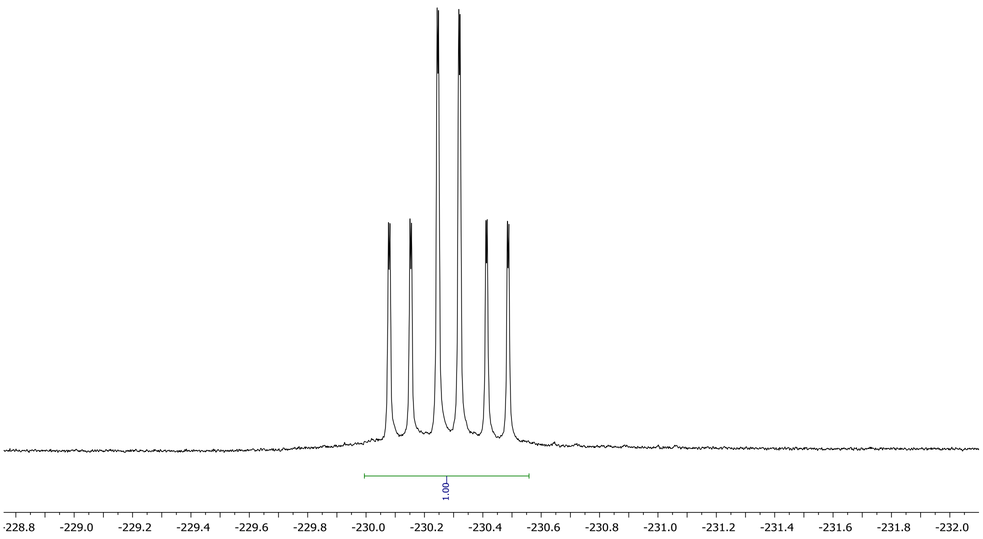

5-(Fluoromethyl)-2-(4-(trifluoromethyl)phenyl)-4,5-dihydrooxazole (2d)

^{1}H NMR (600 MHz, $CDCl_3$, 298 K)

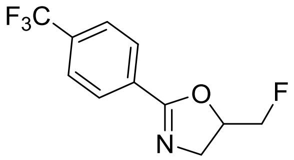

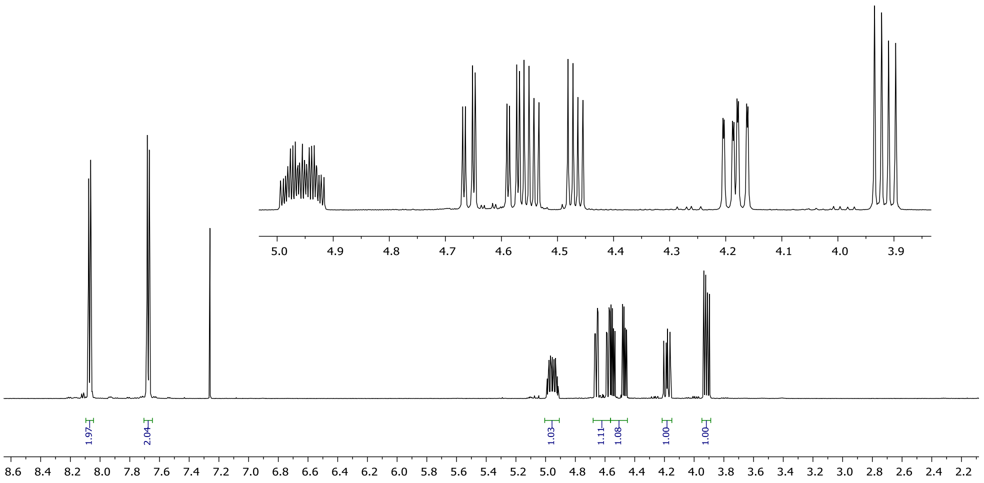

^{13}C{^{1}H} NMR (151 MHz, $CDCl_3$, 298 K)

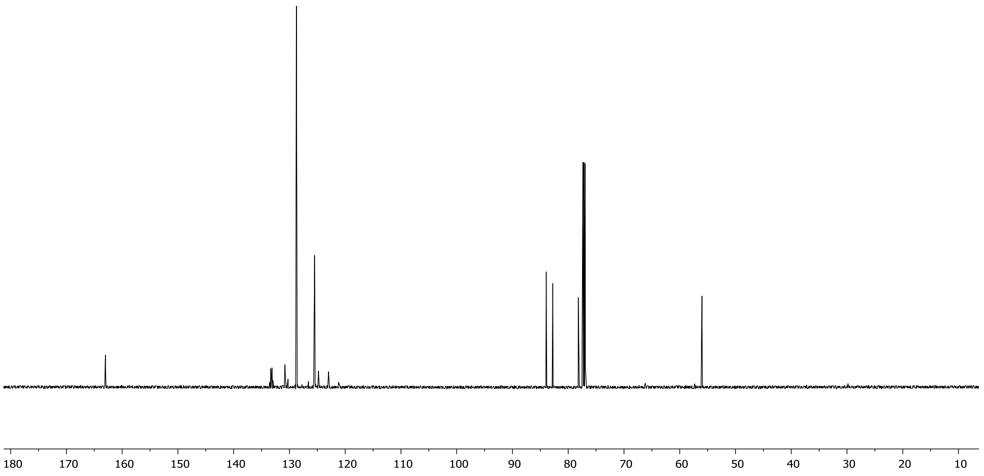

^{19}F NMR (564 MHz, $CDCl_3$, 298 K)

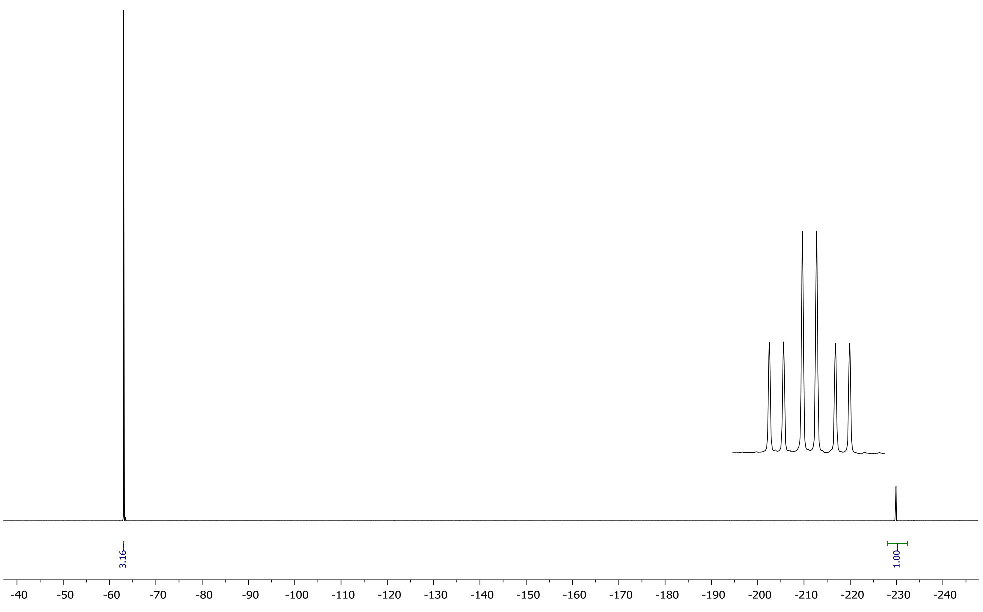

4-(5-(Fluoromethyl)-4,5-dihydrooxazol-2-yl)benzaldehyde (2e)

^{1}H NMR (600 MHz, $CDCl_3$, 298 K)

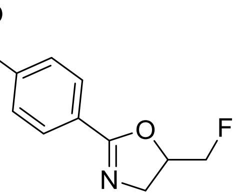

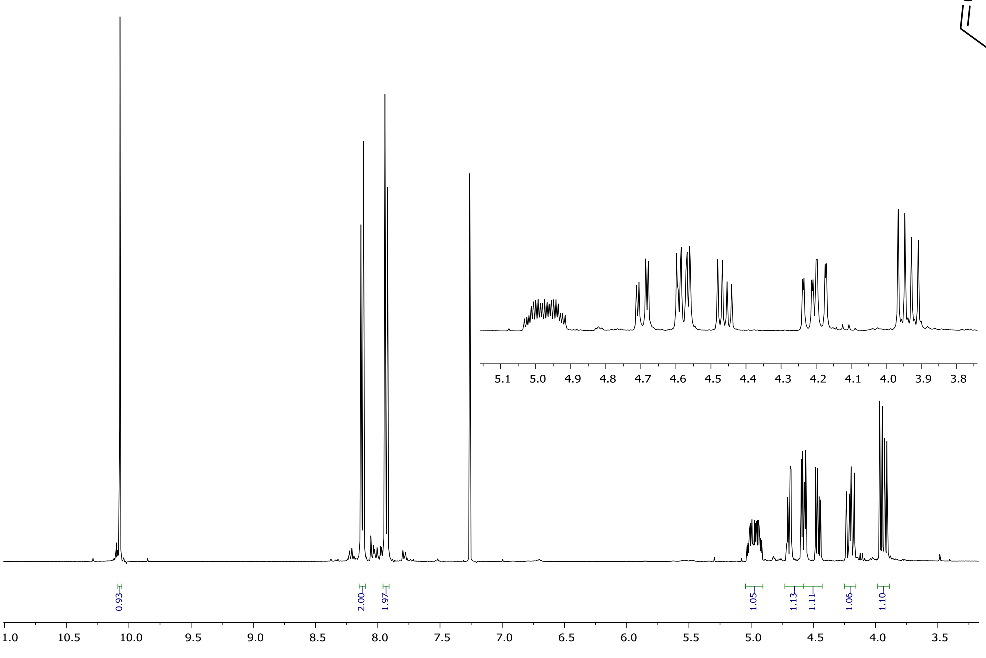

^{13}C{^{1}H} NMR (151 MHz, $CDCl_3$, 298 K)

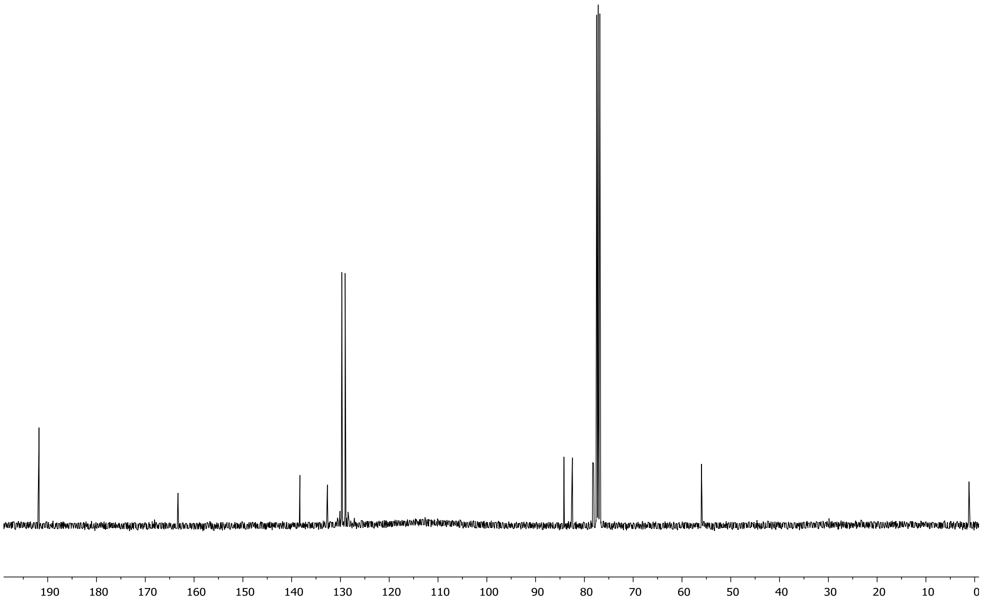

^{19}F NMR (564 MHz, $CDCl_3$, 298 K)

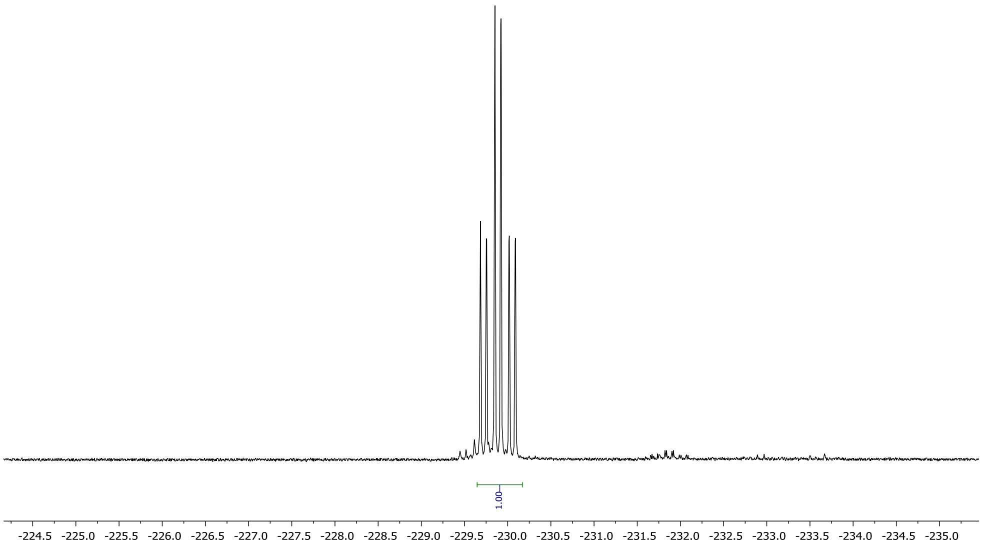

2-(2-Bromophenyl)-5-(fluoromethyl)-4,5-dihydrooxazole (2f)

^{1}H NMR (600 MHz, $CDCl_3$, 298 K)

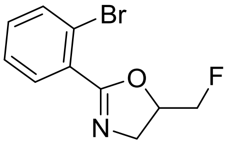

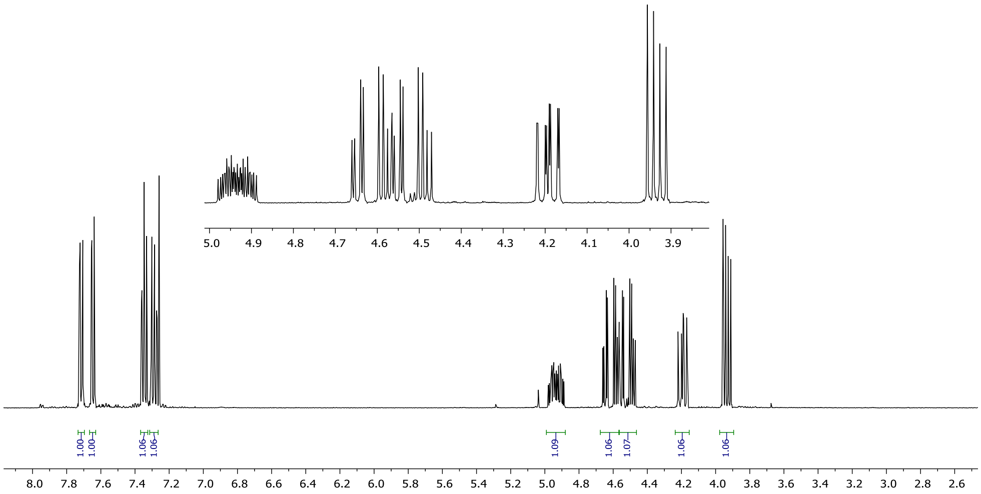

^{13}C{^{1}H} NMR (151 MHz, $CDCl_3$, 298 K)

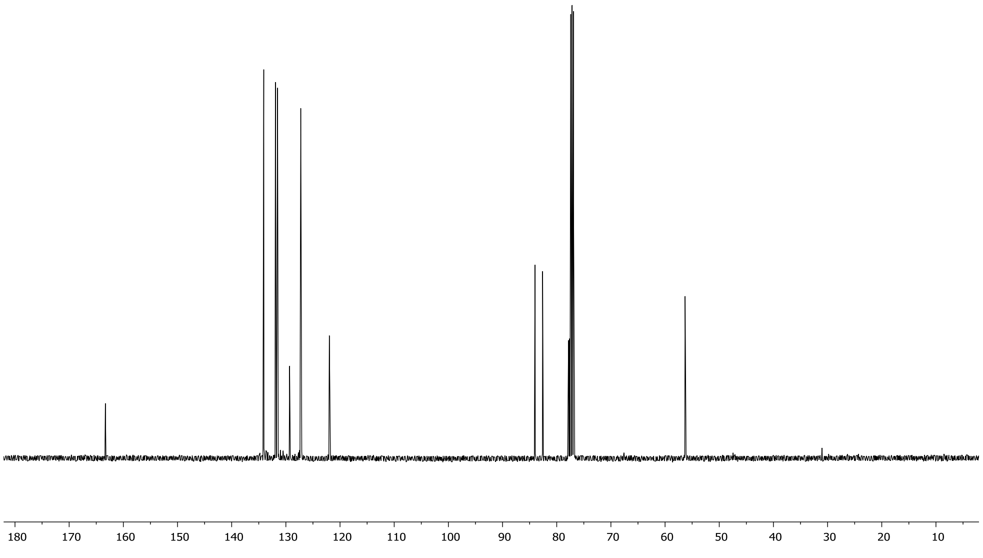

^{19}F NMR (564 MHz, $CDCl_3$, 298 K)

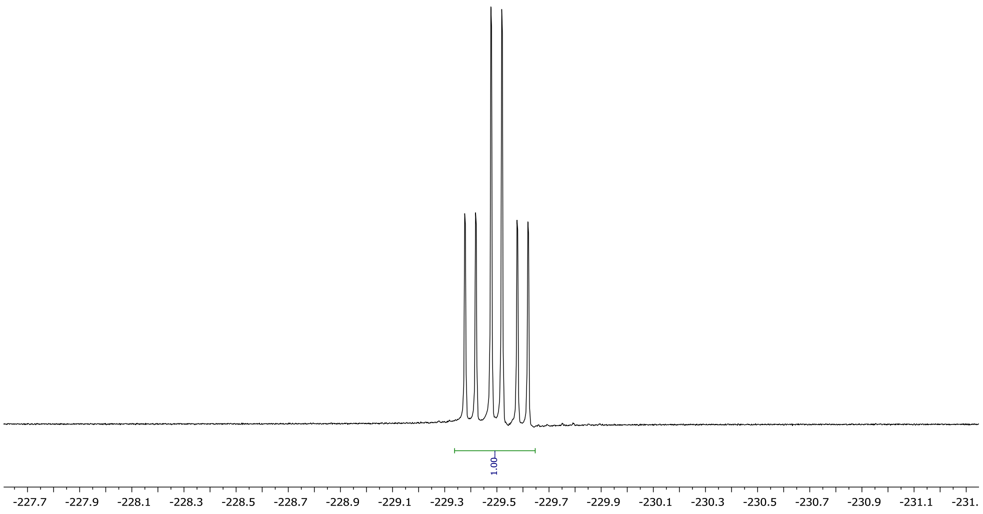

N-(2-(5-(Fluoromethyl)-4,5-dihydrooxazol-2-yl)phenyl)acetamide (2g)

^{1}H NMR (600 MHz, $CDCl_3$, 298 K)

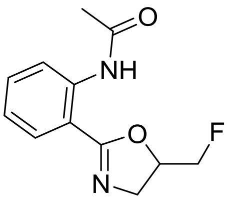

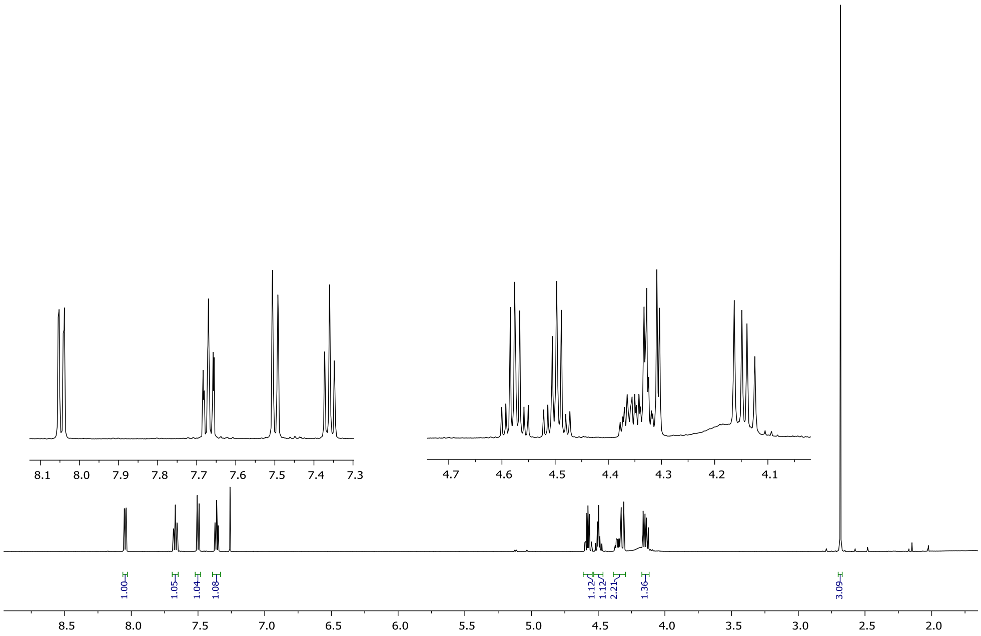

^{13}C{^{1}H} NMR (151 MHz, $CDCl_3$, 298 K)

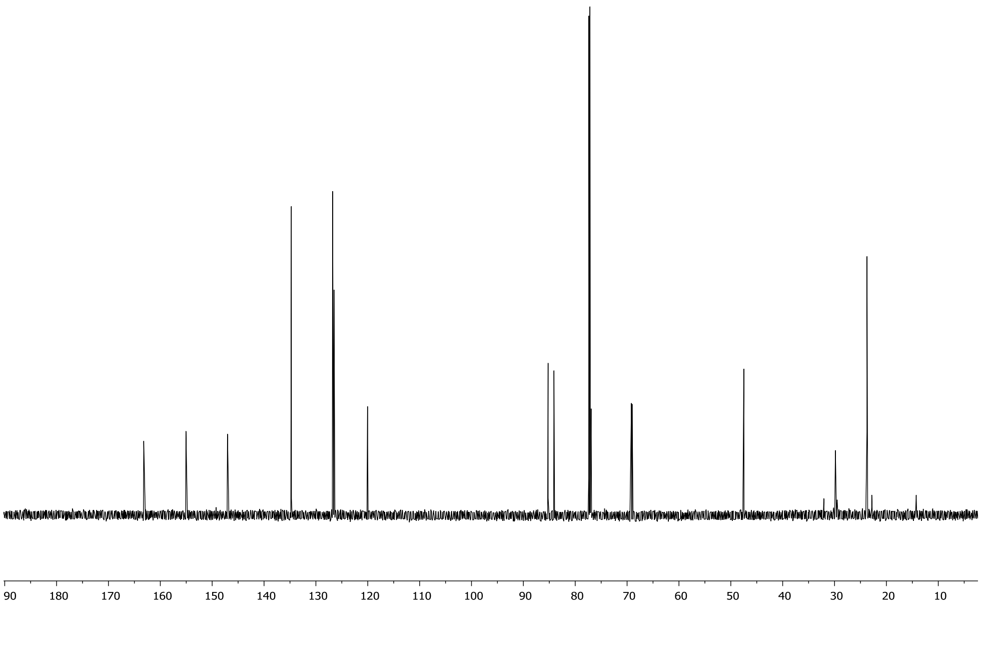

^{19}F NMR (564 MHz, $CDCl_3$, 298 K)

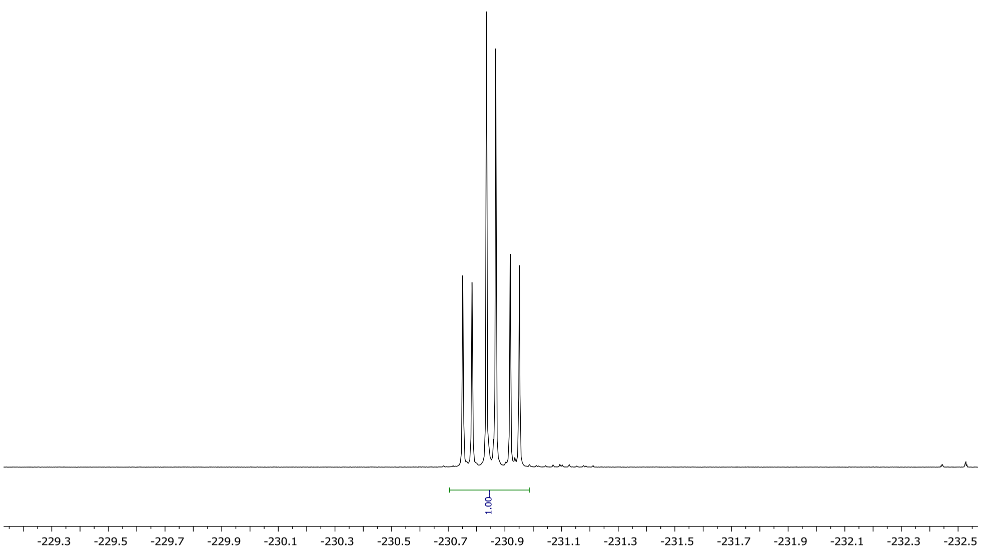

2-(5-Bromo-2-chlorophenyl)-5-(fluoromethyl)-4,5-dihydrooxazole (2h)

^{1}H NMR (600 MHz, $CDCl_3$, 298 K)

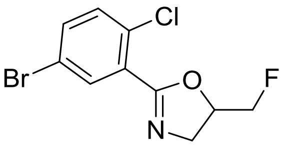

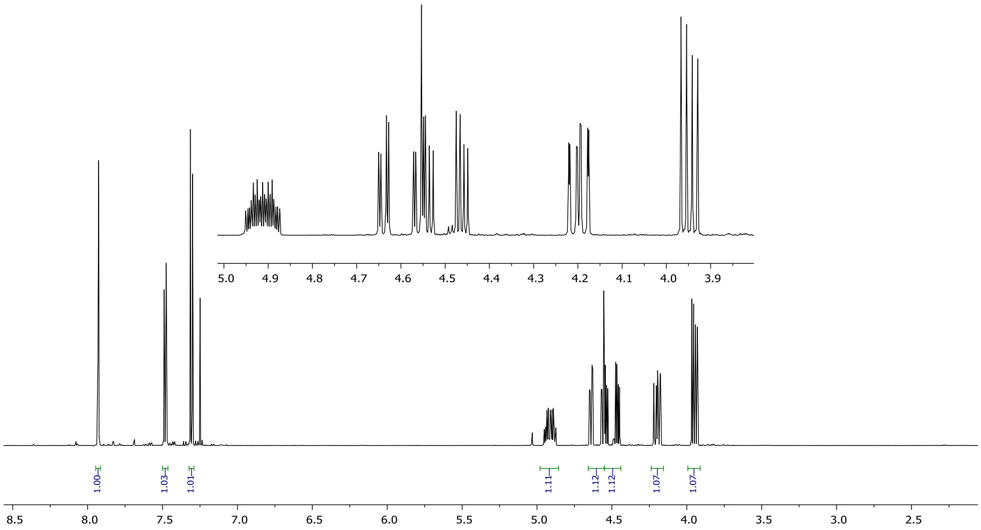

^{13}C{^{1}H} NMR (151 MHz, $CDCl_3$, 298 K)

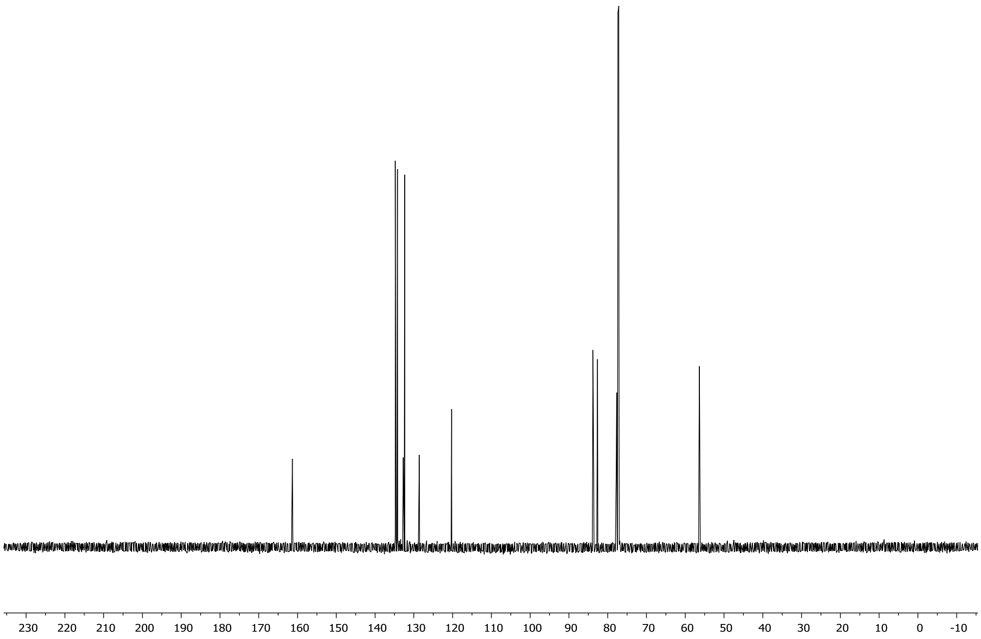

19F NMR (282 MHz, $CDCl_3$, 298 K)

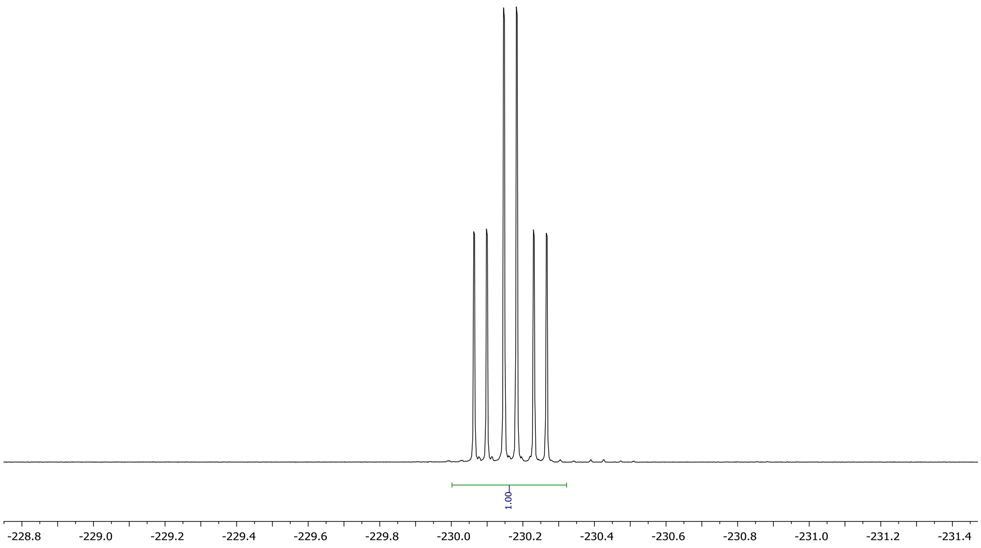

2-(5-(Fluoromethyl)-4,5-dihydrooxazol-2-yl)-4-nitrophenol (2i)

^{1}H NMR (600 MHz, $CDCl_3$, 298 K)

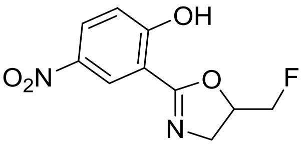

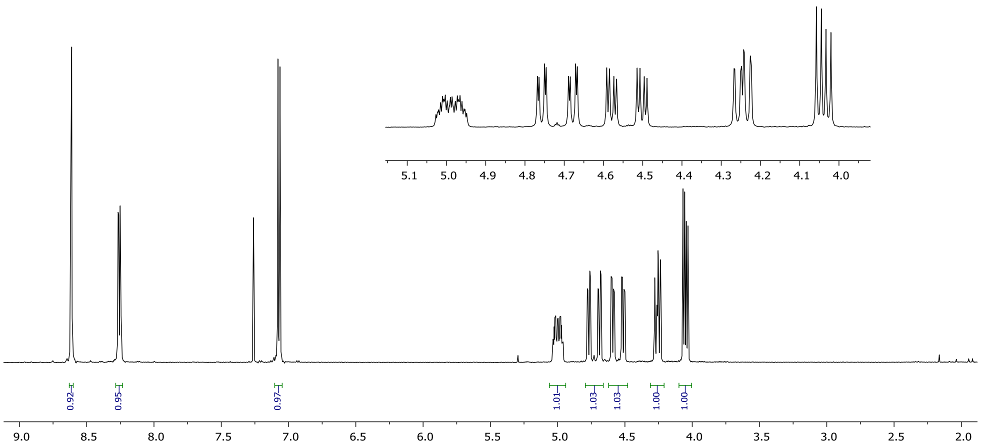

^{13}C{^{1}H} NMR (151 MHz, $CDCl_3$, 298 K)

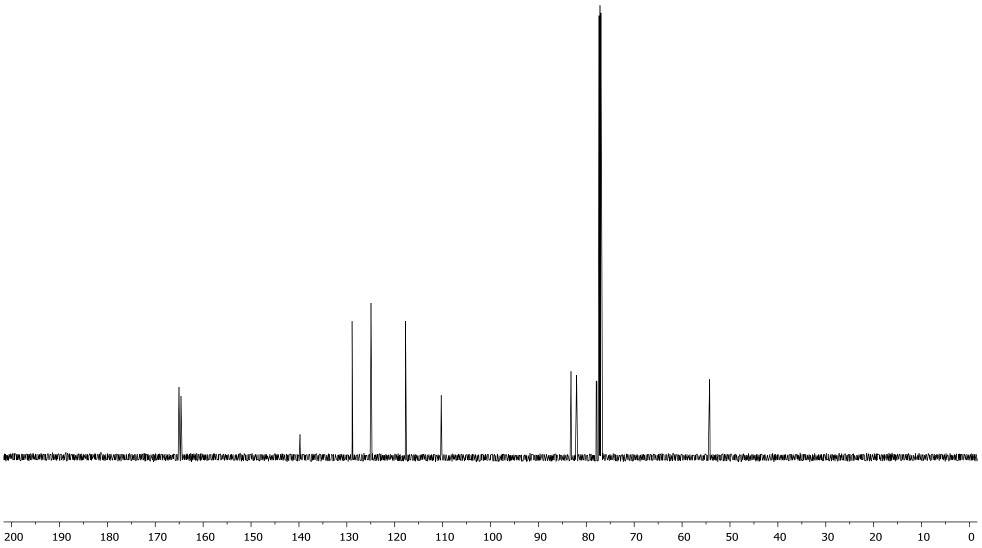

^{19}F NMR (282 MHz, $CDCl_3$, 298 K)

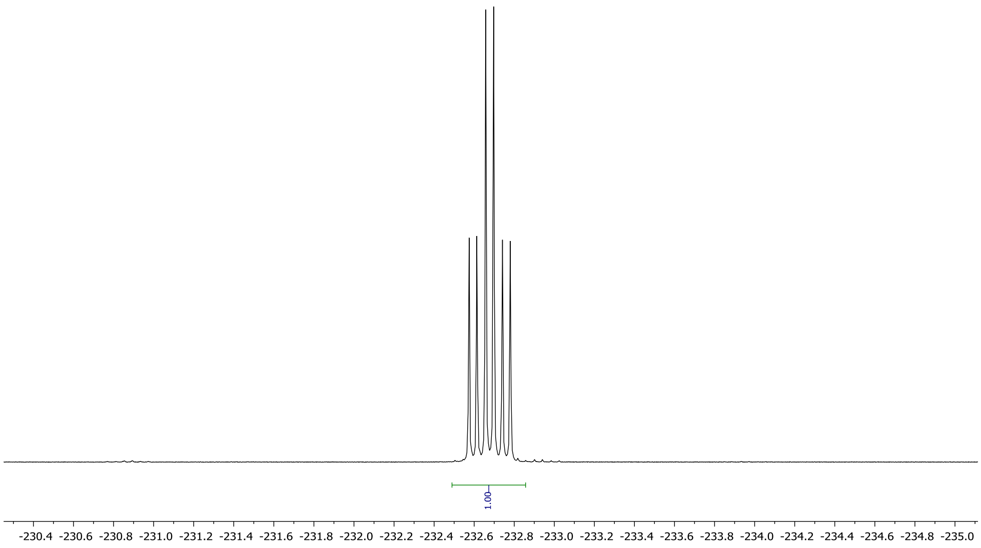

5-(Fluoromethyl)-2-(perfluorophenyl)-4,5-dihydrooxazole (2j)

^{1}H NMR (400 MHz, $CDCl_3$, 298 K)

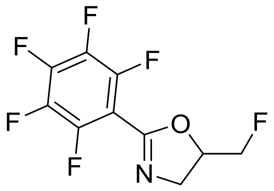

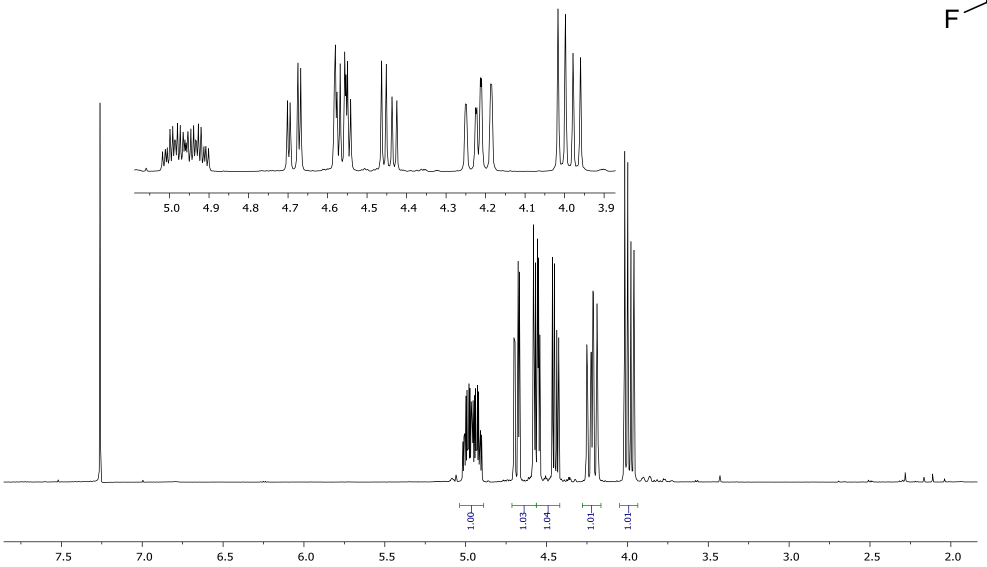

$^{13}C\{^{1}H\}$ NMR (101 MHz, $CDCl_3$, 298 K)

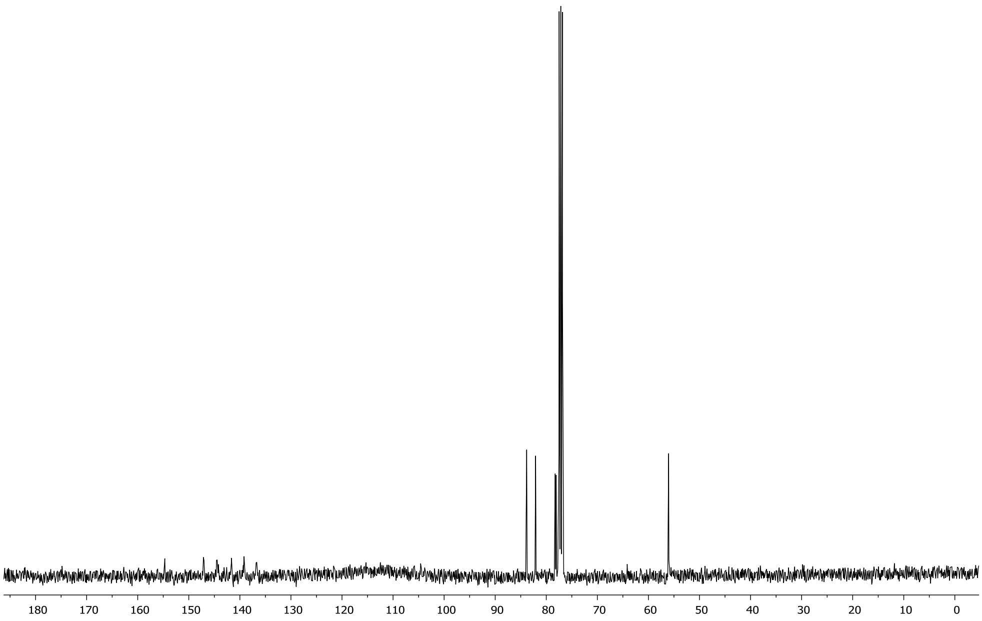

^{19}F NMR (282 MHz, $CDCl_3$, 298 K)

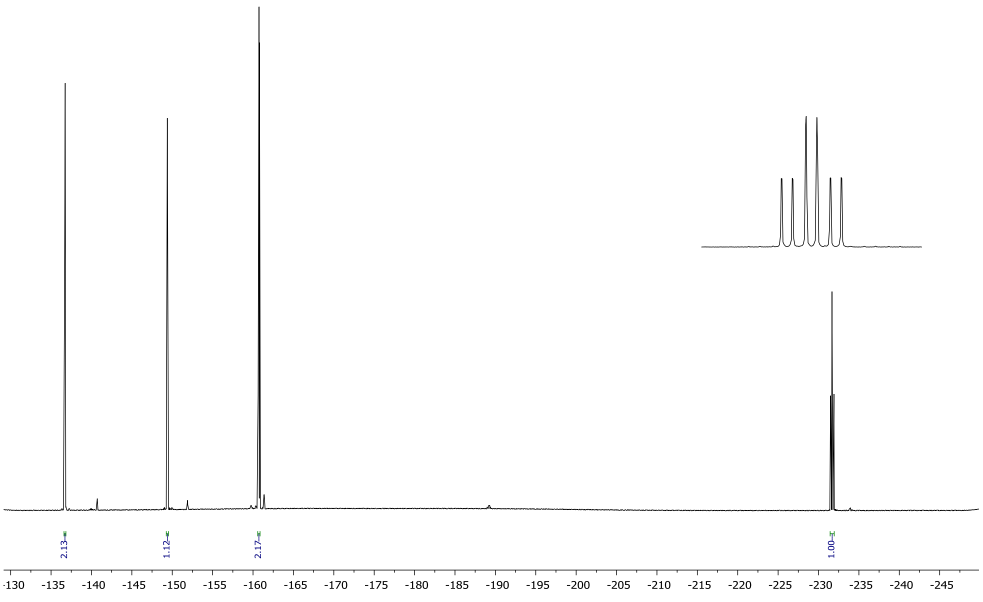

6-(Fluoromethyl)-2-phenyl-5,6-dihydro-4H-1,3-oxazine (2k)

^{1}H NMR (400 MHz, $CDCl_3$, 298 K)

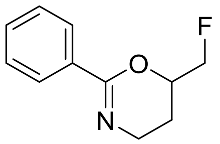

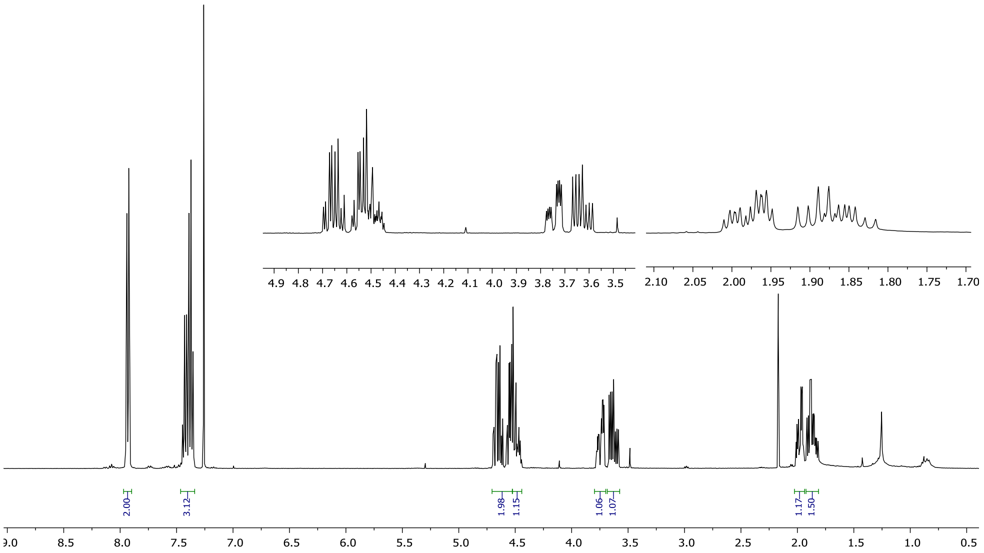

^{13}C{^{1}H} NMR (101 MHz, $CDCl_3$, 298 K)

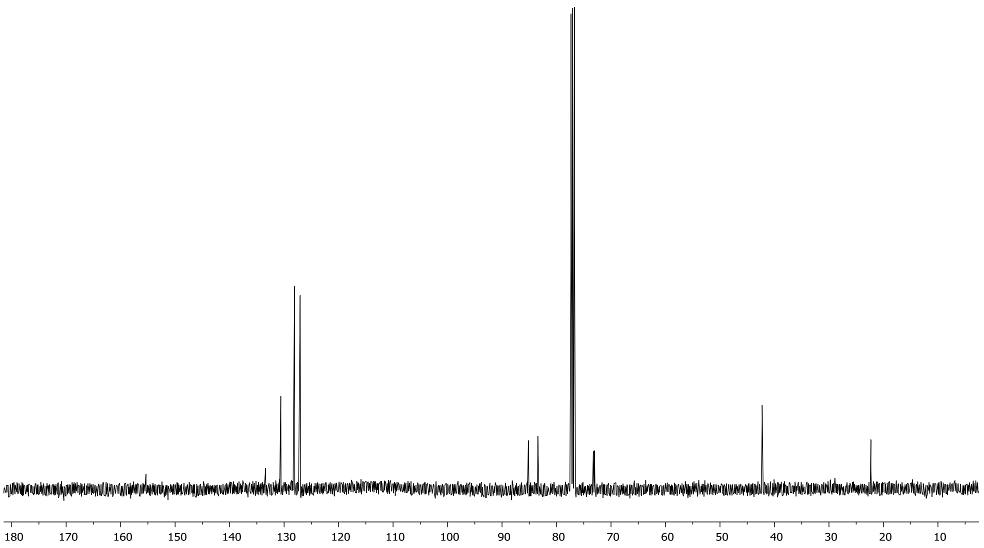

^{19}F NMR (282 MHz, $CDCl_3$, 298 K)

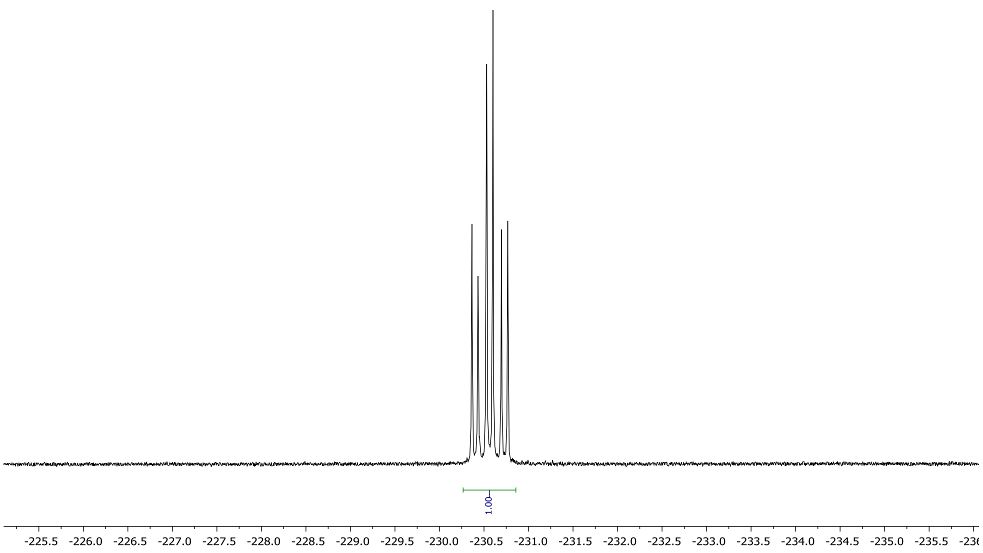

4-(5-(Fluoromethyl)-4,5-dihydrooxazol-2-yl)-9*H*-fluoren-9-one (2m)

^{1}H NMR (600 MHz, $CDCl_3$, 298 K)

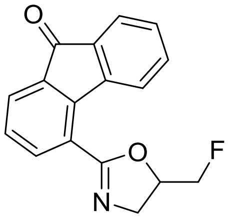

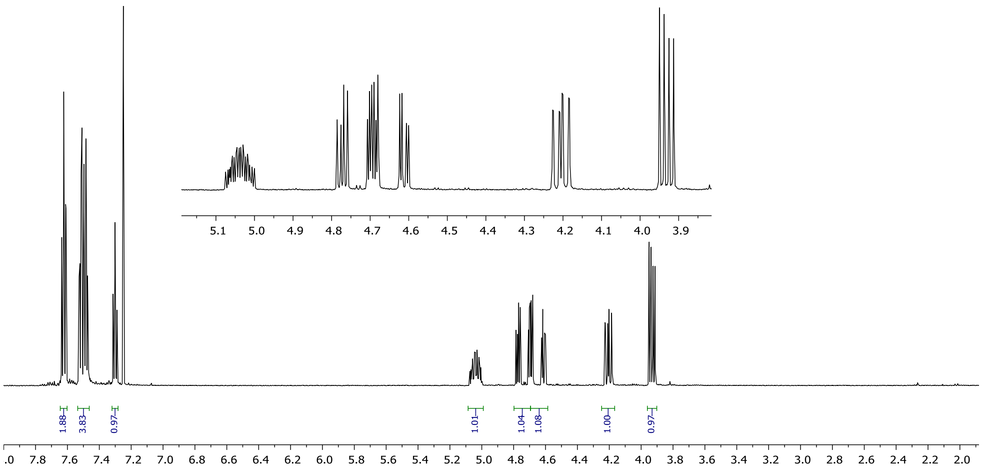

^{13}C{^{1}H} NMR (151 MHz, $CDCl_3$, 298 K)

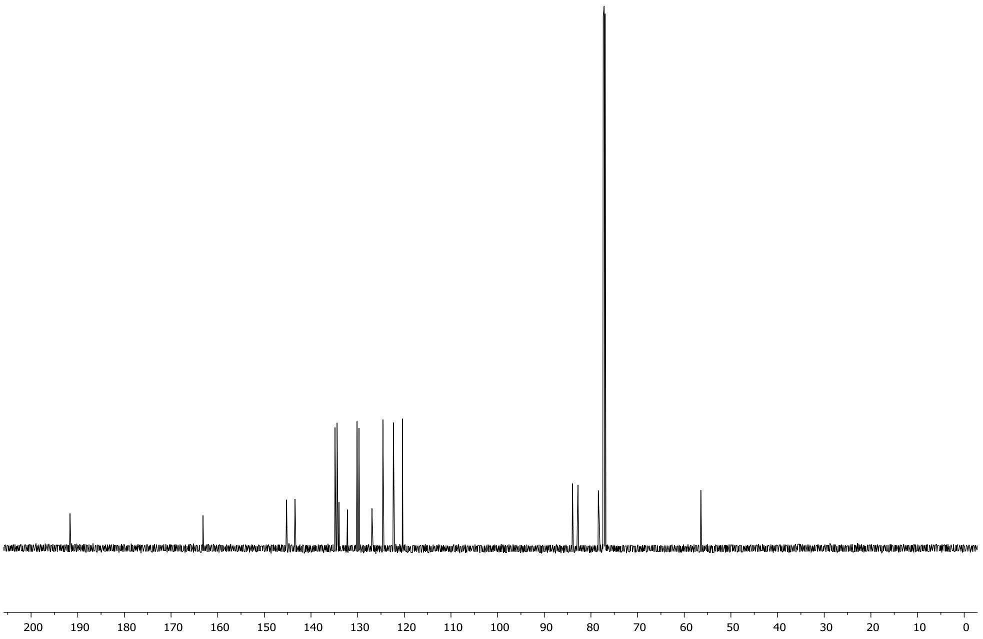

^{19}F NMR (282 MHz, $CDCl_3$, 298 K)

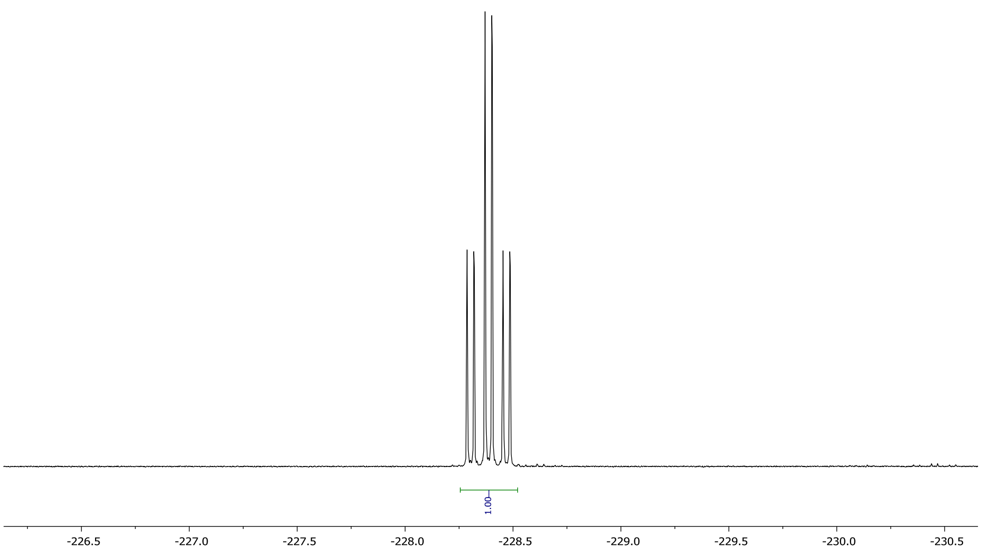

5-(Fluoromethyl)-2-(furan-2-yl)-4,5-dihydrooxazole (2n)

^{1}H NMR (600 MHz, $CDCl_3$, 298 K)

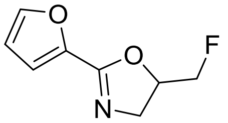

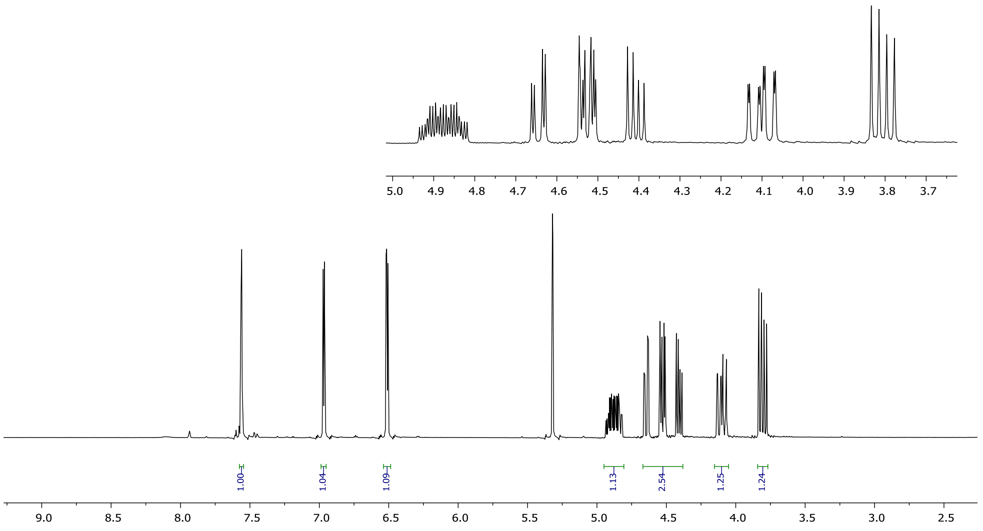

^{13}C{^{1}H} NMR (151 MHz, $CDCl_3$, 298 K)

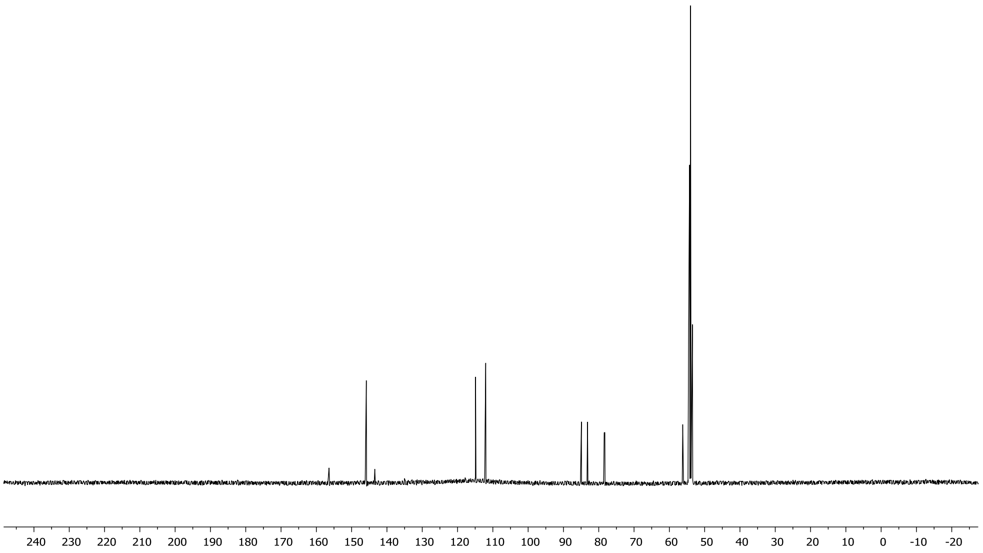

^{19}**F NMR** (282 MHz, $CDCl_3$, 298 K)

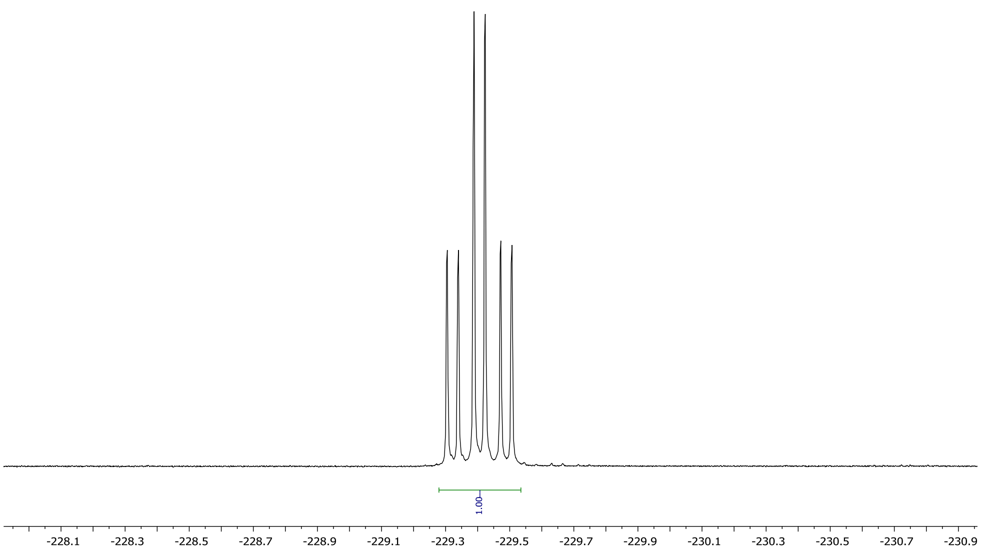

1,3-Bis(5-(fluoromethyl)-4,5-dihydrooxazol-2-yl)benzene (2p)

^{1}H NMR (600 MHz, $CDCl_3$, 298 K)

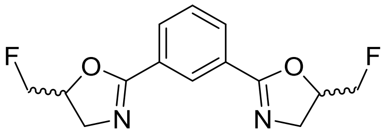

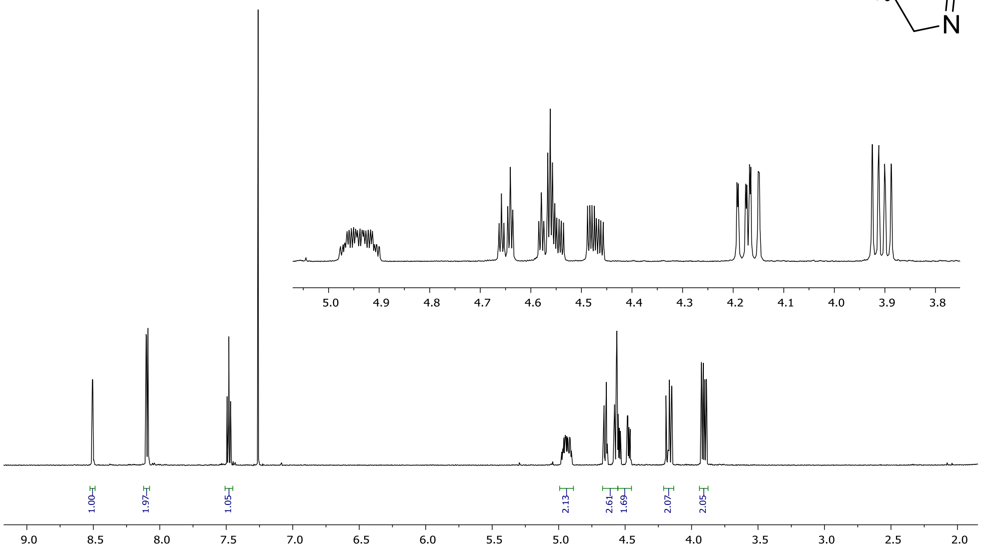

^{13}C{^{1}H} NMR (151 MHz, $CDCl_3$, 298 K)

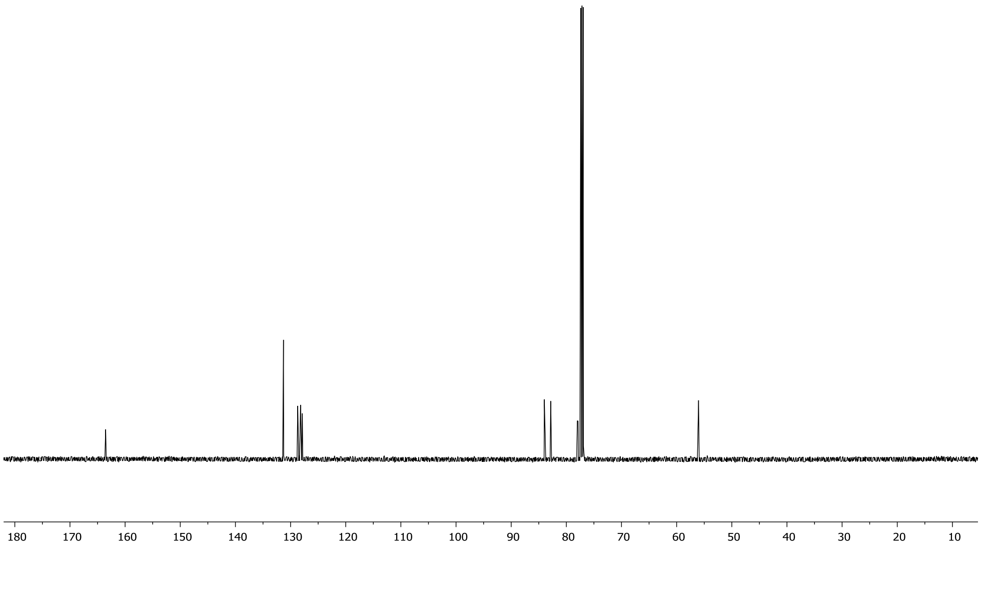

^{19}F NMR (564 MHz, $CDCl_3$, 298 K)

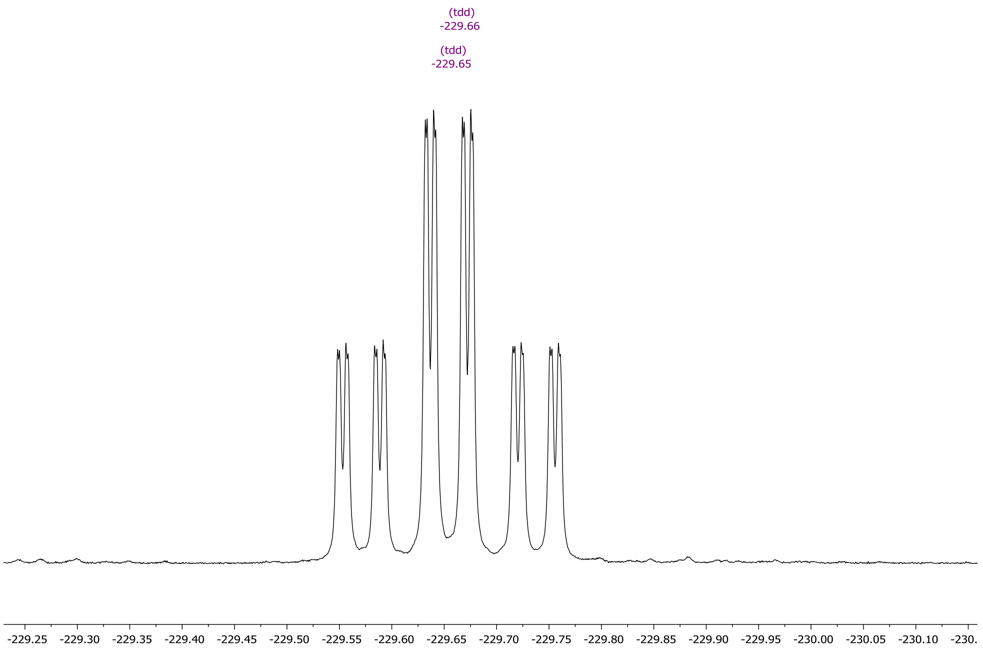

^{19}F{^{1}H} NMR (564 MHz, $CDCl_3$, 298 K)

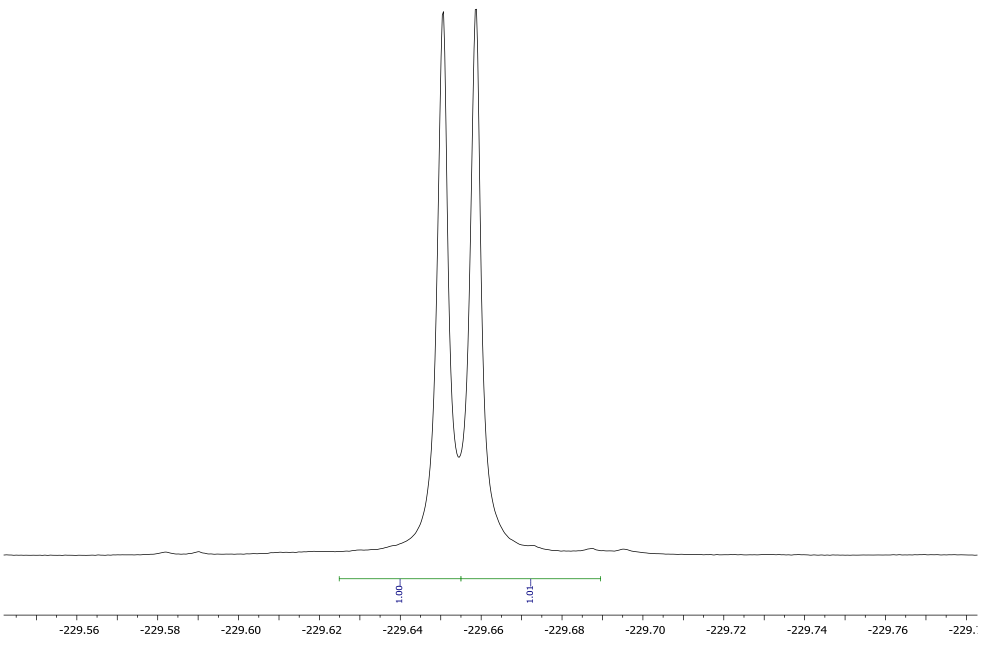

Benzyl 3-(5-(fluoromethyl)-4,5-dihydrooxazol-2-yl)propanoate (2q)

^{1}H NMR (400 MHz, $CDCl_{3,}$ 298 K)

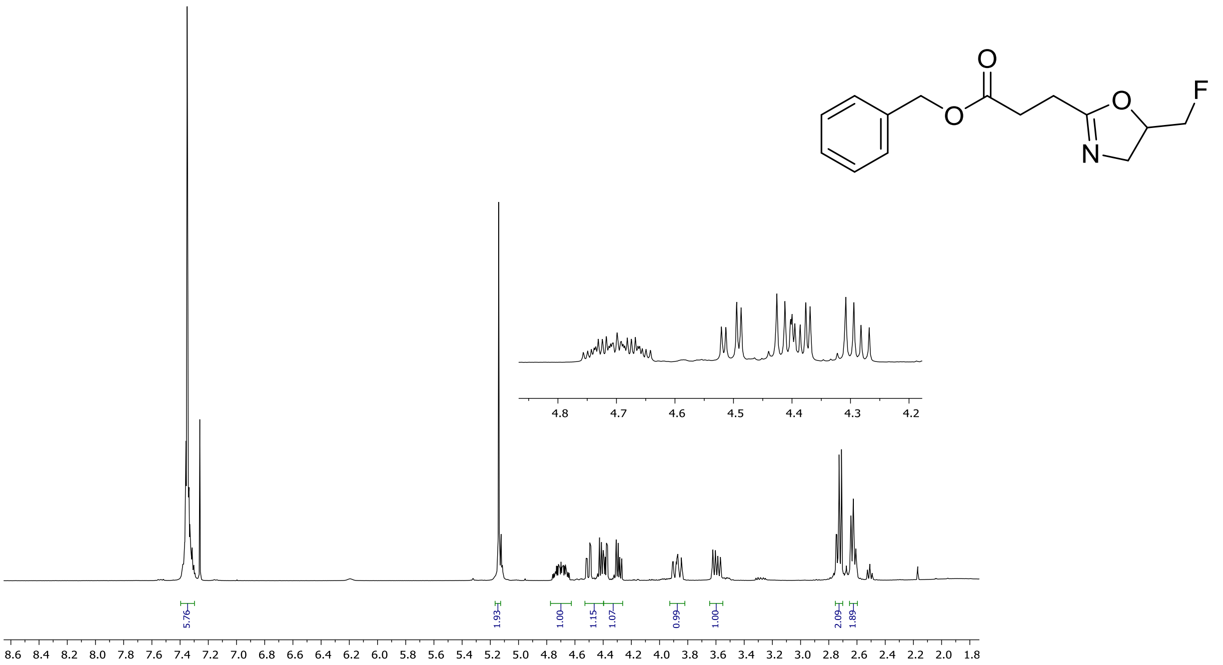

^{13}C{^{1}H} NMR (101 MHz, $CDCl_{3,}$ 298 K)

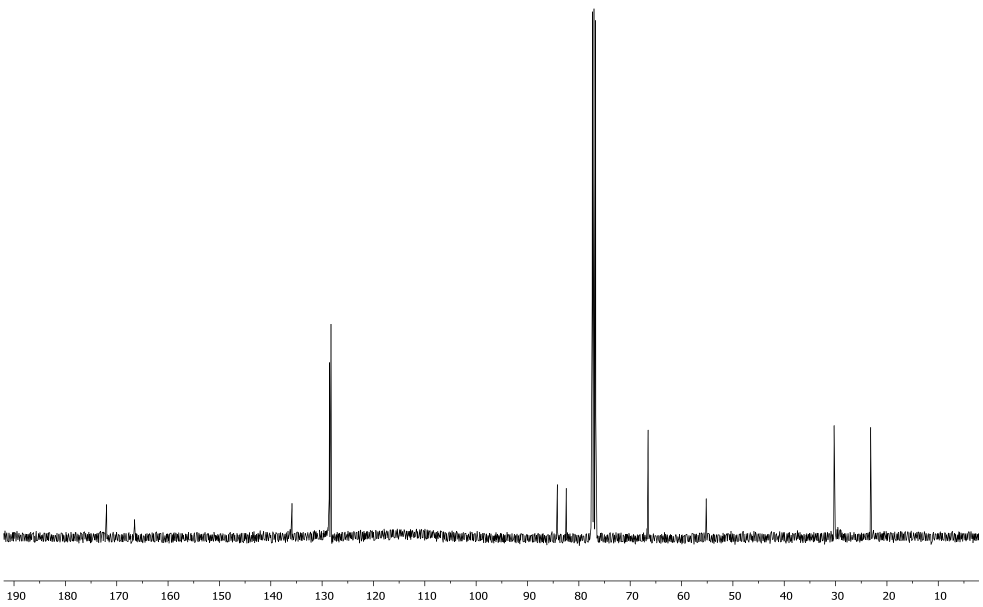

^{19}F NMR (282 MHz, $CDCl_3$, 298 K) (Traces of hydrolysis product <5%)

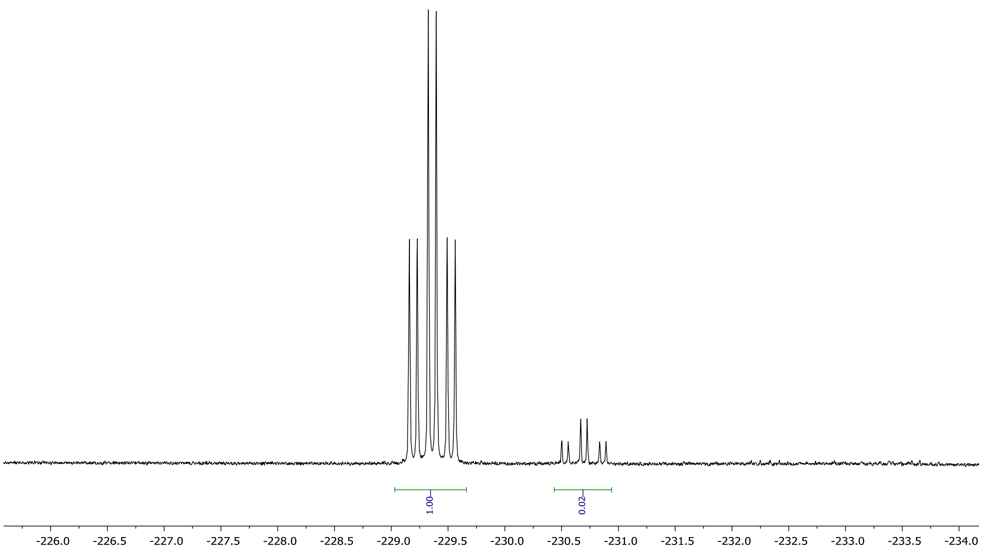

(2*S*)-2-(Benzyloxy)-*N*-(3-fluoro-2-hydroxypropyl)-3-phenylpropanamide (3)

^{1}H NMR (600 MHz, $CDCl_3$, 298 K)

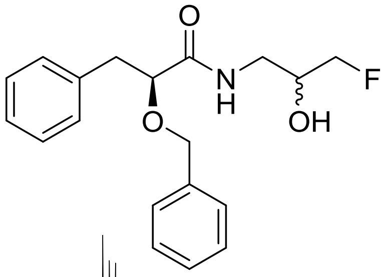

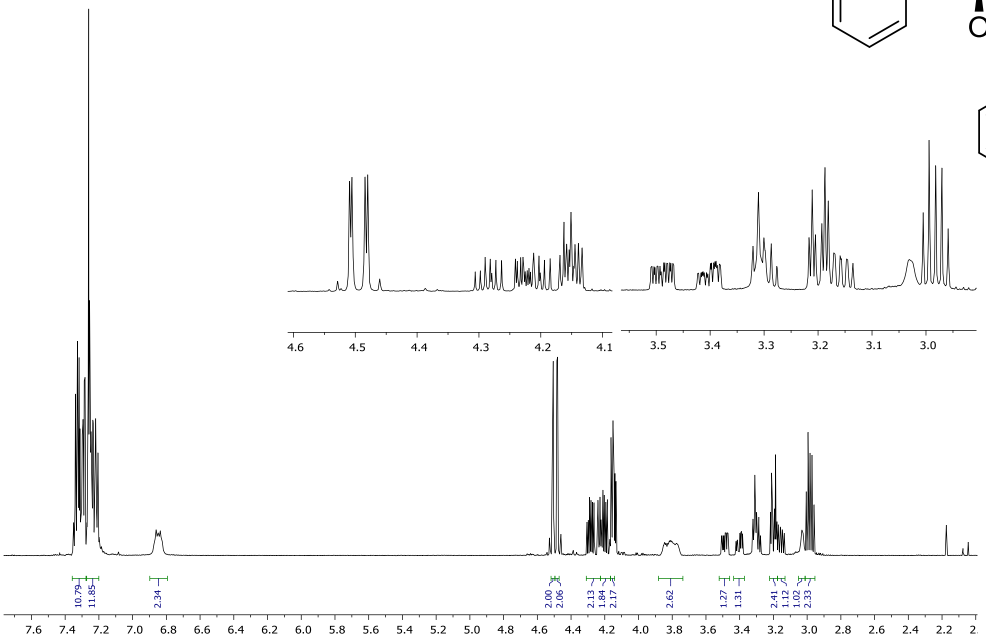

^{13}C{^{1}H} NMR (151 MHz, $CDCl_3$, 298 K)

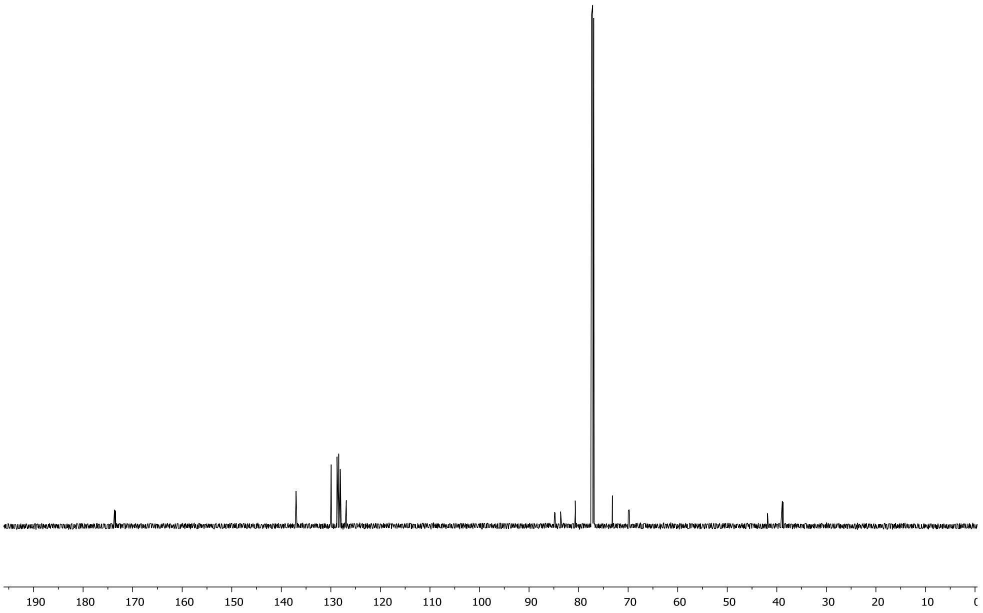

^{19}F NMR (564 MHz, $CDCl_3$, 298 K)

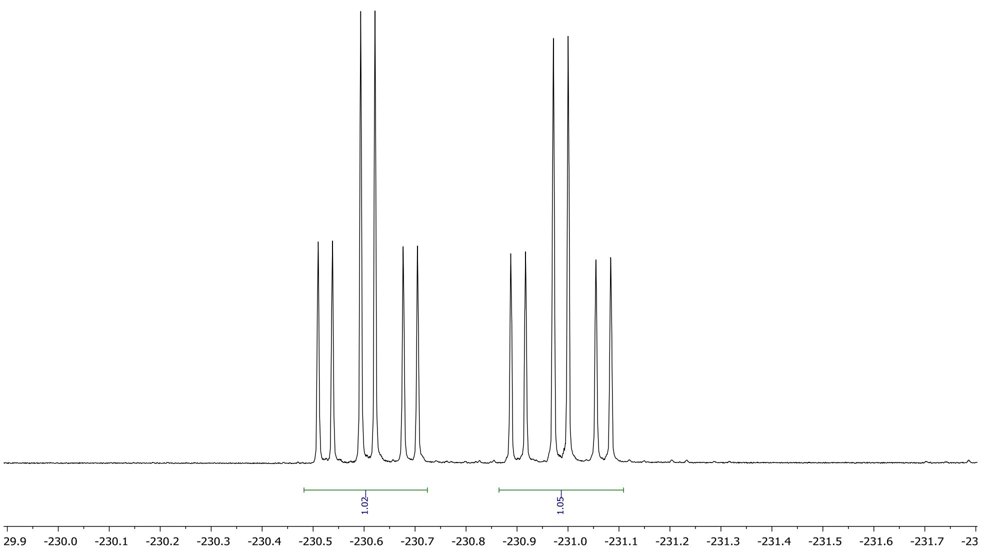

(4*S*,5*R*)-4-Benzyl-5-(fluoromethyl)-2-phenyl-4,5-dihydrooxazole (2s)

^{1}H NMR (600 MHz, $CDCl_3$, 298 K)

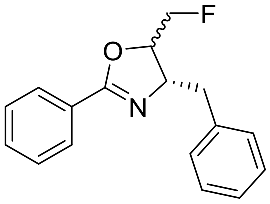

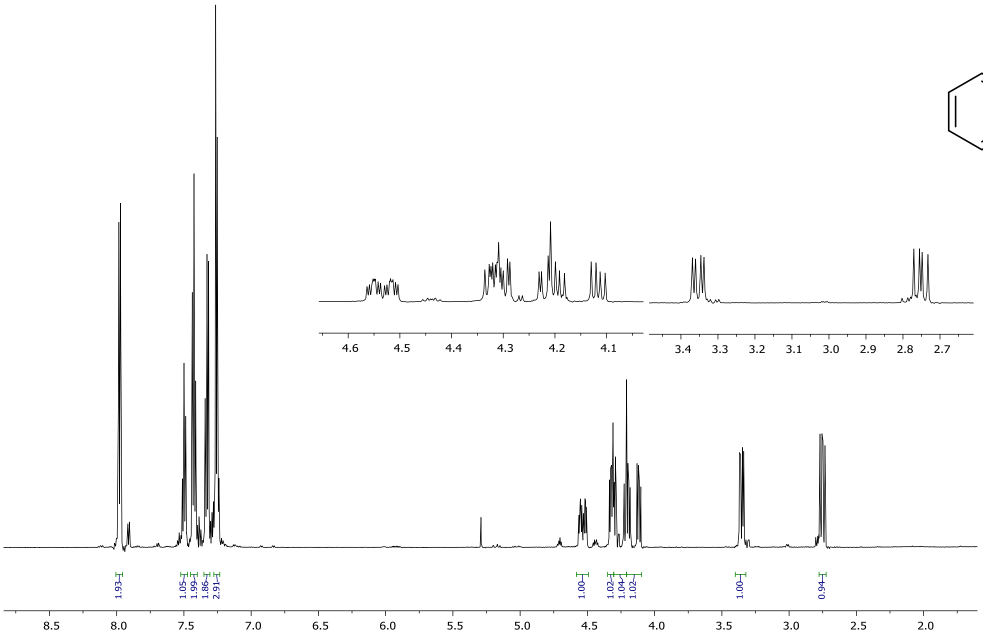

^{13}C{^{1}H} NMR (151 MHz, $CDCl_3$, 298 K)

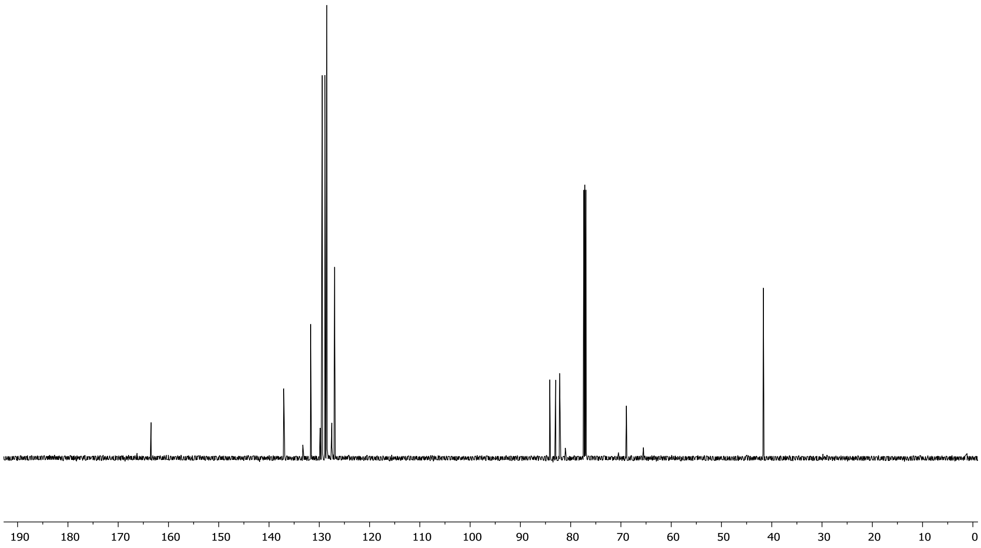

^{19}F NMR (564 MHz, $CDCl_3$, 298 K)

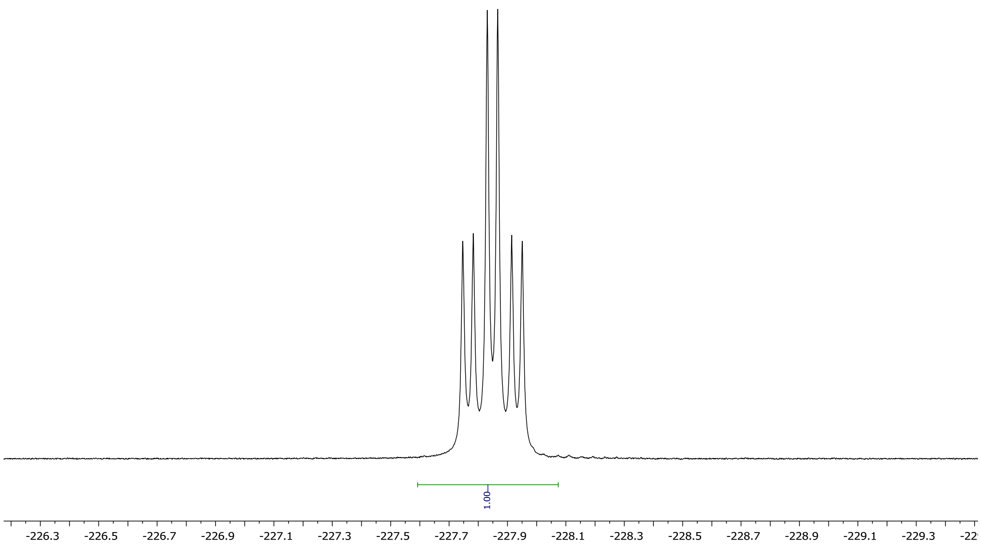

^{19}F{^{1}H} NMR (564 MHz, $CDCl_3$, 298 K)

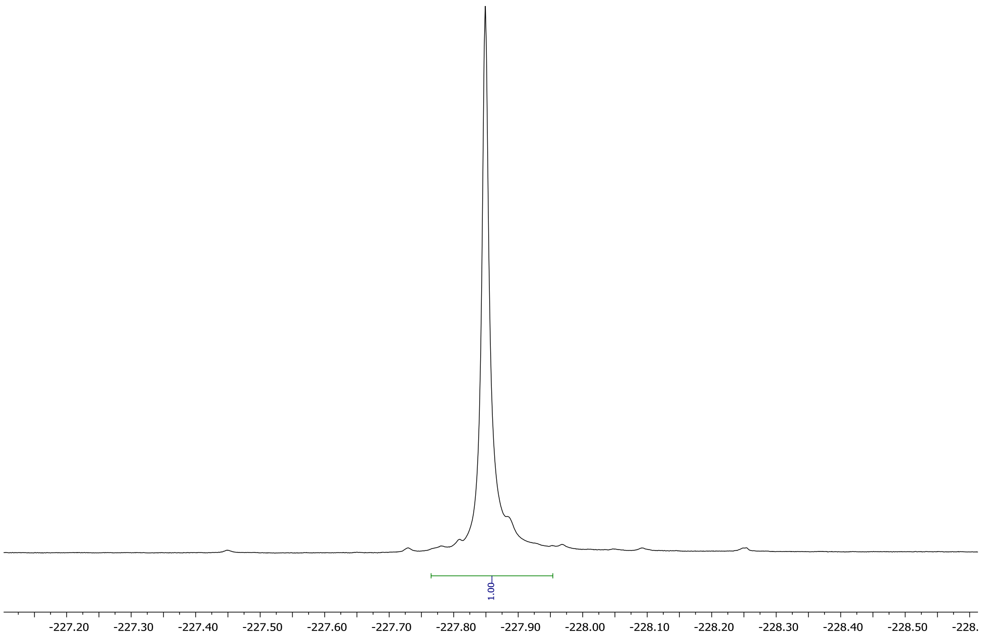

References

[1] *APEX3* (**2016**), *SAINT* (**2015**) and *SADABS* (**2014**), Bruker AXS Inc., Madison, Wisconsin, USA.

[2] *SHELX* software: Sheldrick, G. M. *Acta Cryst.* **2015**, *A71*, 3–8.

[3] P. Prediger, L. F. Barbosa, Y. Génisson, C. R. D. Correia, *J. Org. Chem.* **2011**, *76*, 7737–7749.

[4] P. Baburajan, K. P. Elango, *Tetrahedron Lett.* **2014**, *55*, 1006–1010.

[5] X.-Q. Hao, L.-J. Chen, B. Ren, L.-Y. Li, X.-Y. Yang, J.-F. Gong, J.-L. Niu, M.-P. Song, *Org. Lett.* **2014**, *16*, 1104–1107.

[6] B. Nammalwar, N. P. Muddala, F. M. Watts, R. A. Bunce, *Tetrahedron* **2015**, *71*, 9101–9111.

[7] S. Korsager, R. H. Taaning, A. T. Lindhardt, T. Skrydstrup, *J. Org. Chem.* **2013**, *78*, 6112–6120.

[8] M. Hellal, G. D. Cuny, *Tetrahedron Lett.* **2011**, *52,* 5508–5511.

[9] R. Adepu, B. Prasad, M. A. Ashfaq, N. Z. Ehtesham, M. Pal, *RSC Adv.* **2014**, *4*, 49324–49328.

[10] F. K. I. Chio, S. J. J. Guesné, L. Hassall, T. McGuire, A. P. Dobbs, *J. Org. Chem.* **2015**, *80*, 9868–9880.

[11] E. M. Stang, C. M. White, *J. Am. Chem. Soc.* **2011**, *133*, 14892–14895.

[12] J. Zheng, Z. Deng, Y. Zhang, S. Cui, *Adv. Synth. Catal.* **2016**, *358*, 746–751.

[13] A. Fürstner, T. Gastner, H. Weintritt, *J. Org. Chem.* **1999**, *64*, 2361–2366.

[14] R. Parella, S. A. Babu, *J. Org. Chem.* **2017**, *82*, 6550–6567.

[15] J. L. Sessler, D. An, W.-S. Cho, V. Lynch, M. Marquez, *Chem. Eur. J.* **2005**, *11*, 2001–2011.

[16] M. Sani, P. Lazzari, M. Folini, M. Spiga, V. Zuco, M. De Cesare, I. Manca, S. Dall'Angelo, M. Frigerio, I. Usai, A. Testa, N. Zaffaroni, M. Zanda, *Chem. Eur. J.* **2017**, *23*, 5842–5850.

[17] Y.-C. Wu, J. Zhu, *J. Org. Chem.* **2008**, *73*, 9522–9524.

[18] R. Colombo, Z. Wang, J. Han, R. Balachandran, H. N. Daghestani, D. P. Camarco, A. Vogt, B. W. Day, D. Mendel, P. Wipf, *J. Org. Chem.* **2016**, *81*, 10302–10320.

[19] J. R. Luly, J. F. Dellaria, J. J. Plattner, J. L. Soderquist, N. Yi, *J. Org. Chem*. **1987**, *52*, 1487–1492.
